# What does team science look like across the CTSA consortium? A qualitative analysis of the Great CTSA Team Science Contest submissions

**DOI:** 10.1017/cts.2021.812

**Published:** 2021-07-12

**Authors:** Clara M. Pelfrey, Ann S. Goldman, Deborah J. DiazGranados

**Affiliations:** 1 Clinical and Translational Science Collaborative (CTSC), Center for Clinical Investigation, Case Western Reserve University, Cleveland, OH, USA; 2 George Washington University Milken Institute School of Public Health, Department of Epidemiology, Clinical and Translational Science Institute at Children's National (CTSI-CN), Washington, DC, USA; 3 School of Medicine, Department of Medicine, Wright Center for Clinical and Translational Research, Virginia Commonwealth University, Richmond, VA, USA

**Keywords:** Team science, multidisciplinary science, translational science, CTSA, Qualitative data analysis

## Abstract

**Introduction:**

The Great CTSA Team Science Contest (GTSC) was developed to discover how Clinical and Translational Science Award (CTSA) hubs promote and support team science [1]. The purpose of this study was a secondary qualitative analysis of the GTSC submissions to better understand the diversity of team science initiatives across the CTSA consortium.

**Methods:**

Secondary qualitative analysis of the GTSC data addressed the following research questions, which defined the top-level coding: (1) What CTSA component sponsored it? (2) Who was the team doing the work? (3) Who were the intended beneficiaries? (4) What was the intended outcome? (5) What strategies did they use? (6) What translational science (TS) stage was addressed? (7) How do they align with the NCATS team science strategic goals? (8) How do the CTSA’s team science efforts align with the National Academies Research Council (NRC) recommendations for enhancing the effectiveness of team science?

**Results:**

The GTSC received 170 submissions from 45 unique CTSA hubs. Qualitative analysis revealed a great variety of team science strategies for virtually all team science stakeholders. In addition to strategies to promote team science, results show successful examples that focus on outcomes and illustrate ways of measuring success.

**Conclusions:**

The GTSC shows that the CTSA consortium is involved in an extremely diverse array of team science activities, which align well with both the NRC recommendations for enhancing the effectiveness of team science and the NCATS strategic goals for team science. Future research should evaluate the efficacy of team science strategies.

## Introduction

The scope of team science strategies across the Clinical and Translational Science Award (CTSA) consortium is extremely diverse, but they have not been fully characterized or evaluated for effectiveness. Many CTSA hubs are engaged in promoting team science, however, there is no single definition of what constitutes “team science.” The increasing complexity of Clinical and Translational Science (CTS) problems has prompted large investments in team science initiatives [2–9]. The National Center for Advancing Translational Science (NCATS) supporting the CTSA program has made team science one of its strategic goals with four objectives: (1) Engage patients, community members, and nonprofit organizations in the translational process; (2) Share resources through collaborative research with other NIH institutes and centers; (3) Form innovative collaborations with multidisciplinary scientists at other institutions; and (4) Develop new collaborative structures with the private sector [10].

The complexities of science along with rapid technological advances have created the necessity for scientific collaborations between researchers with complementary expertise. The demand for collaborations has outpaced the development of institutional support, policies, funding opportunities, and team science culture. These gaps gave rise to the Science of Team Science (SciTS), a field of study designed to address the value of team science and develop strategies for facilitating, engaging in, leading, and supporting scientific teams [11]. The SciTS literature contains five key subjects: the value of team science, formation of teams, how team composition affects team performance, qualities of effective teams, and how institutions affect teams [12]. The Great CTSA Team Science Contest (GTSC) data address all these major subjects while providing valuable examples of successful team science across the CTSA consortium.

The GTSC was developed in the NCATS Workgroup on Institutional Readiness for Team Science to collect stories describing how CTSA hubs promote and support team science [1]. The contest invited CTSA hubs to submit important, novel, or impactful ways that they advance team science (see Appendix 1 for the GTSC Instructions and Qualtrics Survey). Here, we conducted a secondary qualitative analysis of the GTSC dataset using a set of research questions to guide the analysis: (1) What CTSA component sponsored the team science activity? (2) Who was the team conducting the team science work? (3) Who were the intended beneficiaries? (4) What was the intended outcome? (5) What method did they use? (6) What translational science (TS) stage was addressed? (7) How do they align with the NCATS team science strategic goals? (8) How do the CTSA’s team science efforts align with the National Academies Research Council (NRC) recommendations for enhancing the effectiveness of team science? Thus, the purpose of this study was to conduct a qualitative analysis of how hubs were engaging in team science and to demonstrate the great breadth of consortium team science activities. In addition, this analysis sought to classify the submissions into meaningful categories as a resource for hubs interested in expanding team science initiatives. This study aims to advance our understanding of the diversity of CTSA team science initiatives including the intended recipients, strategies used, intended outcomes, and metrics for measuring success. Since the GTSC asked for strategies for enhancing team science, the results are discussed in the context of the how well they address the National Research Council’s recommendations for enhancing the effectiveness of team science [13].

## Methods

### GTSC Contest

This paper represents a secondary analysis of an existing data source: The GTSC dataset. The research questions addressed here were developed *after* completion of the contest to mine the contest results for meaningful information about team science. Thus, several submissions do not specify sufficient data to answer the research questions and are classified as “Not explicitly specified.” The GTSC was developed in 2018 by the NCATS Workgroup on Institutional Readiness for Team Science to collect stories describing the ways hubs were promoting and supporting team science across the CTSA consortium. The original contest purpose, rules, and winners are listed on the CLIC website [1]. The contest was designed as a low-burden method to elicit information about the numerous team science strategies within the consortium. A more detailed description of the contest features, the criteria for judging, the results, and the lessons learned can be found in the companion paper [14]. The full-text GTSC submissions are reproduced with submission ID numbers and CTSA hub names in Appendix 2 [15].

### Coding Structure and Analysis

The major research questions were used to organize the top-level coding categories, and inductive processes were used to develop the subcategories, such as the types of team science interventions or the beneficiary of the intervention. Subcategories were verified with more coding examples and similar subcategories were combined (e.g. trainees and students). The goal was to develop a meaningful framework to understand what strategies are being used to advance team science across the CTSA consortium. The qualitative data analysis coding structure with definitions is listed in Appendix 3. All 170 submissions were coded independently by the 3 authors. Where there was disagreement, the final coding assignment was made where two out of the three coders agreed. Many submissions could be coded to more than one subcategory, which more accurately represents the overlapping multidisciplinary nature of team science. When this occurred, the primary team science strategy was used to categorize the submission.

### Research Questions

The research questions were developed using two main criteria: (1) What CTSA hubs would find useful in terms of methods/strategies, intended beneficiaries, intended outcomes, and metrics for measuring effectiveness and (2) To answer the question: How well does team science across the CTSA consortium, as found in the GTSC dataset, address the NRC recommendations for enhancing the effectiveness of team science and the NCATS strategic goals?

This analysis addressed the following research questions: (1) What CTSA component sponsored the team science activity? (2) Who was the team conducting the team science work? (3) Who were the intended beneficiaries? (4) What was the intended outcome? (5) What team science strategy did they use? (6) What TS stage was addressed? (7) How do the findings align with the NCATS team science strategic goals? (8) How do the CTSA’s team science efforts align with the NRC recommendations for enhancing the effectiveness of team science? The analysis also examined both the short-term outcomes for the participants (e.g. researchers, trainees, students, community members) as well as the mid- and long-term outcomes to assess team science success.

## Results

### Q1. What CTSA Component Sponsored the Team Science Activity? and Q2. Who was the team conducting the team science work?

The first two research questions focused on who conducted the work. First, each submission was coded by labeling which CTSA component conducted the work using the components from the 2018 CTSA Funding Opportunity Announcement [16] (Table 1). Second, each submission was coded to identify the team who was conducting the work (Table 2). For the first research question, it was predicted that the Community and Collaboration core would be the primary sponsors of the work submitted via the GTSC. This was true for 43 submissions (Table 1). Some submissions highlighted community engagement studios or team science training led by the Community and Collaboration core. On the other hand, the Network Capacity core was explicitly mentioned only once (e.g. Trial Innovation Network (TIN)). The second research question proved more difficult to code, as the team conducting the work was not always explicitly stated. The category with the most team science submissions was multidisciplinary teams. Several submissions highlighted collaborations between persons evaluating the team science work and the CTSA team science experts. While 58 of the submissions were coded as being conducted by multidisciplinary teams, 71 submissions did not clearly specify who conducted the work (Table 2). These results show that the CTSA infrastructure is now an integral part of many institutions and therefore team science is not done by any single group or core.

**Table 1.** Which Clinical and Translational Science Award (CTSA) component or core sponsored the team science intervention?

CTSA component or core^*^	Number of cases	Cases coded – by submission ID number
Community and Collaboration	43	9, 16, 18, 19, 25, 26, 29, 33, 35, 39, 42, 46, 49, 50, 54, 65, 66, 67, 73, 82, 83, 84, 85, 90, 95, 96, 111, 113, 114, 115, 118, 123, 133, 141, 142, 147, 149, 155, 157, 159, 163, 166, 170
Hub Research Capacity	8	25, 37, 41, 79, 82, 83, 106, 149
Informatics	6	10, 28, 38, 65, 118, 133
Network Capacity	1	156
Research Methods	5	10, 63, 108, 127, 153
Translational Endeavors	33	1, 3, 5, 7, 15, 20, 23, 27, 39, 44, 47, 49, 64, 71, 77, 89, 113, 114, 118, 124, 127, 133, 135, 137, 141, 149, 151, 152, 154, 159, 163, 164, 169
CTSA component was not explicitly specified within the submission	107	2, 4, 6, 7, 8, 11, 12, 13, 14, 15, 17, 18, 19, 20, 21, 22, 23, 24, 27, 30, 31, 32, 34, 36, 38, 39, 40, 41, 42, 43, 44, 45, 48, 51, 52, 53, 55, 56, 57, 58, 59, 60, 61, 62, 64, 68, 69, 70, 72, 74, 75, 76, 78, 80, 81, 86, 87, 88, 91, 92, 93, 94, 97, 98, 99, 100, 101, 102, 103, 104, 105, 107, 109, 110, 112, 116, 117, 119, 120, 121, 122, 125, 126, 128, 129, 130, 131, 132, 134, 136, 138, 139, 140, 143, 144, 145, 146, 148, 150, 158, 160, 161, 162, 165, 167, 168, 170

* The 2018 Funding Opportunity Announcement PAR-18-464 for the CTSA program includes the following components: Network Capacity, Hub Research Capacity, Community/Collaboration, Translational Endeavors, Research Methods, Informatics [16].

**Table 2.** Who was the team conducting the team science work?

Team	Number of cases	Cases coded – by submission ID number
Community	10	16, 18, 25, 57, 67, 73, 96, 142, 166, 167
CTSA core	53	1, 3, 8, 10, 15, 20, 22, 23, 27, 28, 33, 35, 37, 38, 43, 44, 46, 47, 49, 54, 55, 56, 63, 67, 71, 78, 79, 82, 85, 104, 105, 107, 111, 113, 114, 116, 122, 123, 125, 128, 129, 135, 136, 140, 141, 147, 149, 151, 152, 153, 154, 155, 156
KL2	4	16, 89, 90, 159
Multi-CTSA hubs	9	19, 21, 38, 48, 75, 77, 105, 106, 169
Multidisciplinary teams	58	2, 4, 7, 9, 11, 12, 15, 17, 18, 19, 20, 25, 28, 29, 30, 33, 35, 39, 40, 41, 42, 43, 44, 48, 49, 52, 54, 62, 65, 69, 70, 72, 73, 76, 79, 90, 91, 96, 106, 115, 126, 132, 133, 137, 141, 142, 143, 150, 157, 158, 162, 163, 164, 165, 166, 168, 169, 170
Team was not explicitly specified within the submission	71	5, 6, 13, 14, 17, 22, 24, 26, 27, 31, 32, 34, 36, 37, 39, 45, 46, 48, 50, 51, 53, 58, 59, 60, 61, 64, 66, 68, 70, 74, 80, 81, 83, 84, 86, 87, 88, 92, 93, 94, 95, 97, 98, 99, 100, 101, 102, 103, 106, 107, 108, 109, 110, 112, 116, 117, 118, 119, 120, 122, 124, 130, 131, 138, 140, 145, 146, 154, 156, 157, 160

CTSA, Clinical and Translational Science Award.

### Q3. Who Were the Intended Beneficiaries?

The intended beneficiaries for the submissions included the following groups (case numbers in parentheses): team scientists (119), the community (21), patients (6), and students (24) (Table 3). Team scientists included creating/fostering multidisciplinary teams, team science training programs, establishing funding mechanisms, research on team science, training research staff, community-based projects, promotion and tenure (P&T), and multi-CTSA hub initiatives. The next largest category was the community in interventions such as joint university–community research projects and improving clinical and public healthcare systems. Patients directly benefitted when they were included in the design of research studies which focused on clinical trials and improving patient services in healthcare settings. Undergraduate and graduate students included medical students, masters, and PhD students in public health and health professions, as well as engineering and law. Postgraduate students included KL2 and TL1 scholars who participated in training courses and exercises to improve team function.

**Table 3.** Who is the intended beneficiary of the intervention?

Beneficiary	Number of cases	Cases coded – by submission ID number
**Team scientists**	119	
• Foster multidisciplinary teams	58	9, 12, 13, 19, 21, 23, 25, 26, 27, 28, 29, 30, 32, 33, 34, 35, 36, 38, 39, 41, 43, 47, 48, 51, 53, 64, 65, 66, 67, 74, 75, 76, 79, 86, 89, 90, 95, 98, 99, 102, 103, 107, 110, 114, 115, 118, 119, 122, 125, 143, 144, 147, 160, 161, 162, 165, 168, 170
• Team science training	24	6, 8, 45, 50, 55, 56, 71, 78, 84, 85, 87, 88, 97, 93, 111, 112, 123, 124, 127, 129, 134, 135, 136, 137
• Funding for researchers including in the community	11	20, 31, 62, 91, 92, 94, 100, 116, 117, 128, 148
• Research on team science	11	2, 3, 7, 22, 63, 109, 130, 131, 132, 163, 169
• Training for program managers, staff, coordinators, community members	6	1, 37, 70, 81, 82, 83
• Community-based projects	4	18, 44, 141, 157
• Promotion and tenure and multi-CTSA hub initiatives	5	15, 46, 106, 108, 146
**Community**	21	11, 14, 54, 57, 60, 61, 69, 72, 73, 80, 96, 101, 104, 120, 126, 139, 142, 158, 164, 166, 167
**Patients included in study design**	6	42, 77, 133, 149, 150, 156
**Students**		
• Undergraduate and graduate (engineering, health care/sciences)	16	5, 10, 16, 17, 24, 40, 49, 52, 58, 59, 68, 138, 140, 145, 151, 153
• KL2/TL1 scholars	8	4, 105, 113, 121, 152, 154, 155, 159

CTSA, Clinical and Translational Science Award.

### Q4. What was the Intended Outcome?

The intended outcome of the GTSC submissions fell into four major subcategories, as shown in Table 4: A. Team science skills and process; B. Getting the community involved in research; C. Other team science activities not otherwise specified; D. Examples of multidisciplinary collaborative teams (not shown).

**Table 4.** What was the intended outcome of the team science submission?

Intended outcome	Number of cases	Cases coded – by submission ID number^*^
**A. Team science skills and process**		
1. Training in team science competencies	24	55, 56, 71, 76, 78, 81, 85, 87, 92, 93, 111, 112, 123, 131, 129, 130, 134, 135, 136, 138, 147, 152, 155, 163
2. Training in communication skills	8	2, 45, 50, 84, 88, 98, 109, 151
3. Providing pilot funding or specific awards to promote existing team science	4	26, 95, 104, 159
4. Team science competency training of students: undergraduates, graduates, and postdoctoral students	7	5, 17, 24, 69, 140, 145, 154
5. Developing a team charter or engaging in team building methods	5	13, 22, 23, 36, 124
6. Leadership or mentorship training	5	3, 4, 97, 113, 121
7. Training in technology ventures or entrepreneurship	3	49, 68, 148
**B. Getting the community involved in translational research**		
1. Partnering with community members and involving the community in research studies in community settings	26	8, 14, 16, 18, 20, 33, 44, 54, 57, 60–61, 64, 67, 73, 74, 82, 96, 115, 120, 128, 142, 149, 157, 158, 166, 167
2. Providing targeted funding opportunities that specifically address community health needs	3	133, 141, 164
**C. Other team science activities**		
1. Fostering *new* multidisciplinary collaboration (e.g. retreats, new pilot grants, hackathons)	33	9, 15, 19, 27, 30, 31, 32, 34, 35, 47, 48, 58, 66, 70, 75, 79, 86, 89, 90, 91, 94, 100, 102, 105, 114, 116, 119, 122, 143, 156, 160, 161, 168
2. Development of new tools in informatics (e.g. for collaborator discovery, funding discovery, team function)	6	41, 53, 107, 117, 118, 144
3. Workforce development	6	1, 11, 37, 52, 83, 162
4. Expanding translational science (TS) capacity	10	6, 10, 43, 77, 80, 106, 110, 127, 137, 153
5. Promotion and tenure (P&T) for team science	3	46, 108, 146

* Examples of specific team science stories did not have a specific strategy with an intended outcome, and thus are not shown in Tables 4 and 5.

#### A. Team science skills and process

The subcategory of team science skills and process was very broad. The primary goal or intended outcomes included the following areas from most to least (number of submissions in parentheses): 1. Training in team science competencies (24); 2. Training in communication skills (9); 3. Providing pilot funding or specific awards to promote existing team science (4); 4. Training undergraduates, graduates, and postdoctoral students in team science (7); 5. Developing a team charter or engaging in team building (5); 6. Leadership or mentorship training (5); and 7. Training in technology ventures or entrepreneurship (3). Each area is described below with specific examples.

**Training in team science competencies** covered 24 evidence-based competencies involving the 7 dimensions of leadership, communication styles and adjusting for different styles, and workshops on leading multidisciplinary teams. For some hubs, the outcome was better team science leadership, whereas others were developing every aspect of researchers’ teams. Some innovative programs were designed to promote team science leadership skills in underrepresented postdoctoral fellows. Importantly, team science competencies were not just for leaders, but some focused on training the research staff to help the entire team function more efficiently.

**Training in communication** was prominently represented and took many forms, such as workshops designed to promote smoother team functioning or end-of-meeting debriefs to clarify the meeting outcomes and next steps. A more complex workshop involved faculty and community members together focusing on public understanding of science and dissemination of results. Communication training included community members, researchers, trainees/students, and clinicians.

**Providing pilot funding and special awards** for promising teams is a frequently used strategy to promote team science. Sometimes a new funding mechanism incentivized the formation of a multidisciplinary team with researchers or community members with common interests. To promote community research on real-world problems, some pilot programs created community member review committees for input into pilot funding decisions. To promote entrepreneurs’ ideas getting into the real world, experts in dissemination and implementation collaborated on business/marketing plans to move the ideas forward as commercial ventures.

**Training undergraduate, graduate, and postdoctoral students** improves the team science pipeline. Several student training programs taught team science competencies, methods, team building, entrepreneurial skills, real-world observations, and analysis of teams. Student training often encompassed longer-term sessions with a week-long residential boot camp or a semester-long course. Several student-focused trainings put students onto existing collaborative teams where they engaged in real-world problem-solving.

**Team building and developing team charters** are important aspects of the team science process which detail many aspects of team function including direction, roles, and how team members should interact. Team charters were regularly mentioned by participants as one of the most useful outcomes of training. Other team building outcomes included improved distance collaboration and building multidisciplinary teams following facilitated brainstorming sessions. Some team building provided a facilitated residential event to create research proposals to address a grand challenge.

**Leadership or mentorship training** was one component of multifaceted programs that also addressed innovation, adaptation, project management, and communication. Training varied from 2-hour workshops to 12-month programs and several leadership programs are actively evaluating their outcomes.

**Developing technology or entrepreneurial skills** focused on developing technological solutions for specific unmet healthcare needs. Others supported increasing awareness of entrepreneurial activity and the potential to commercialize ideas, reinforcing the practice of team science for health science innovation.

#### B. Getting the community involved in research

**A key component to getting the community involved in** TS was engaging community members in determining the specific needs of the community. There were two subcategories of team science submissions that involved the community: (1) Partnering with community members by making them part of a community advisory board, a stakeholder panel, or providing direct advice to researchers on the conception, design, and execution of the study in community settings (26); (2) Providing targeted funding opportunities that address community health needs and incentivize researchers to include community members on the team (3).

#### C. Other team science activities

**Fostering new multidisciplinary collaborations,** an outcome in 33 submissions, included bringing together researchers from different disciplines to brainstorm solutions to a specific challenge. Strategies for creating collaborations included: (1) Hosting one-time events focused on a particular health problem to foster collaboration between multidisciplinary researchers; (2) Matchmaking among researchers from different disciplines using social network analysis, speed networking or, for example, paring physicians who needed medical devices with design engineers; (3) Creating ongoing formal research networks; (4) Providing unique grant mechanisms to foster multidisciplinary research and incentivize new collaborations; (5) Connecting CTSA structures, such as the TIN, with community organizations; (6) Creating formal coursework to train researchers how to create and maintain multidisciplinary collaborations; (7) Creating programs to train researchers to be entrepreneurs.

**Multidisciplinary development of new informatics or biostatistical tools** was a unique type of submission (6). These were collaborations between bioinformatics researchers, computational biologists, or biostatisticians who were developing devices or apps to solve specific problems. They included: (1) Medical apps to manage chronic conditions; (2) Informatics solutions for data discovery such as finding collaborators, biomarkers or developing algorithms; (3) Developing repositories to link data to patients’ electronic health records; (4) Medical device development or improvement (5) Using videogames to train healthcare students; (6) Programs to train informatics students or emergency healthcare professions to work in multidisciplinary teams.

**Workforce development with research personnel** included training health equity researchers, health system leaders, and community health workers in programs that integrate transdisciplinary research, community activation, education, and policy translation (6). Other programs trained research coordinators or managers. Collaborative educational programs enhanced the skills of healthcare students to interact with a community partner on a healthcare challenge.

**Expanding capacity to conduct TS** consisted of developing new systems or relationships to increase TS (10). Examples include new partnerships with scientific journals, developing new MS programs in data science across multiple departments using a shared governance model, and connecting regional health systems to the TIN to facilitate community-based trials.

**Changing promotion and tenure (P&T) policies for team science** was in only three submissions. One story helped chairs understand what it takes for researchers to get P&T as a team scientist. Another submission involved extensive studies of the faculty, chairs, leadership, and the institutional climate to understand the attitudes and barriers to changing team science P&T policies. Finally, a team science symposium was held on ways to advance team science.

#### D. Examples of multidisciplinary team science

There were many submissions that described a specific successful example of multidisciplinary collaborative teams doing disease-specific or wellness interventions. These covered areas of wellness/prevention, chronic disease, cardiovascular disease, mental/behavioral health, addiction, genetics, infectious disease, reducing health disparities, cancer, and others. These generally did not detail a specific intended outcome and are not described in greater detail. If an example described a team science intervention with an intended outcome, then it was moved to the relevant intended outcome category.

**Evaluation of team science strategies** can reveal more effective methods for producing productive teams. Table 5 lists the evaluation metrics divided into short-, mid-, and long-term outcomes from the team science interventions. Short-term outcomes measured immediate changes in knowledge, attitudes, or beliefs (e.g. higher confidence, improved satisfaction). The midterm outcomes documented changes in behavior (e.g. writing publications and grants, applying for patents). Long-term impacts showed changes in conditions (e.g. better health of patients, program was expanded beyond the hub, increased efficiency). Among efforts to encourage team approaches, some leadership assessment programs developed evidence-based competencies or behavioral exemplars, others developed specific evaluative criteria, and some developed programs to educate health system leaders as well as community health workers. Using evaluation of factors such as target customers, demand, and competition, one group developed a private sector tool that accurately predicts the commercial viability of ventures. A few programs randomized participants to control or experimental groups for the intervention, however, no GTSC submissions reported teams’ outcomes over time.

**Table 5.** Evaluation metrics: short-, mid-, and long-term outcomes from team science interventions

Team science evaluation outcomes	#	Cases coded^**^
**Short-term outcomes (changes in knowledge, attitudes, beliefs)**		
Retrospective-pre/post-survey self-assessment: significant improvements in communication, leadership, empowerment and motivation, coaching, and community	2	81, 145
Improved satisfaction of team participants/staff/patients	2	76, 85
Improved feelings of psychological safety	1	85
Trainees recognize advantages of engaging in interdisciplinary interactions	1	154
Higher confidence in conducting clinical research	1	154
**Midterm outcomes (changes in behavior)**		
Improved team function (e.g. mutual respect, adopt a team charter, better teamwork)	3	76, 135, 145
Publish scientific papers	9	7, 9, 49, 94, 120, 142, 144, 157, 158
Patent applications, invention disclosures	3	49, 91, 100
Develop a registry	4	43, 53, 58, 120
Develop educational tools/modules	4	80, 120, 127, 153
Develop apps/digital tools for health management	2	86, 133
Create start-up company	3	49, 68, 100
Develop a medical device	3	49, 143, 161
**Long-term outcomes (changes in conditions; impacts)**		
Policy changes (e.g. promotion and tenure policies; public health policies)	5	9, 14, 46, 54, 60, 61
Expanding translational science capacity (e.g. clinical trials in infants, focus groups for community input, workforce development)	4	14, 44, 128, 166
Program was expanded beyond one hub	5	3, 53, 94, 106, 115
Teams receive extramural grants or state/industry funding	13	9, 19, 33, 44, 89, 94, 100, 104, 114, 141, 144, 161, 166
Research awards and honors	1	144
Better health, screening, and referral, access; lower death rate; shorter length of stay (e.g. for patients, especially for underserved populations)	9	54, 118, 137, 141, 145, 157, 158, 164, 167
Increased efficiency (saved money, saved time, more efficient workflow)	3	106, 118, 157
Establish a new educational organization for collaboration	2	6, 58
New intramural grant program established	2	58, 119
Commercialize a product	1	148

** (Table 5) Submissions may have more than one evaluation outcome so cases may be listed more than once.

### Q5. What Team Science Method did they use?

There were two types of submissions: (1) Team science research, interventions, or resources (133) and (2) Unique examples of successful teams (37). The first category revealed several types of team science interventions including team science training programs (61), the development of new teams (43), community collaboration with teams (20), and other team science methods that did not fit into the previous categories (9). Table 6 shows a breakdown of the methods used.

**Table 6.** What team science method did they use?

Method	Number of cases	Cases coded – by submission ID number
**Team science research, intervention, or resource**	133	
1. Training program for team science	61	1, 3, 4, 5, 7, 8, 10, 11, 13, 17, 22, 23, 24, 37, 45, 46, 49, 50, 55, 56, 63, 68, 71, 76, 78, 81, 83, 84, 85, 87, 88, 92, 93, 94, 97, 98, 106, 109, 111, 112, 113, 121, 123, 124, 127, 129, 130, 131, 132, 134, 135, 136, 137, 138, 140, 145, 151, 152, 153, 154, 155
2. Developing new teams	43	12, 15, 19, 20, 26, 27, 30, 32, 31, 35, 36, 43, 47, 57, 58, 66, 75, 79, 86, 89, 91, 95, 99, 102, 104, 105, 107, 110, 114, 116, 117, 119, 122, 125, 133, 141, 147, 148, 159, 160, 161, 163, 169
3. Community collaboration with teams	20	14, 16, 18, 25, 33, 52, 54, 64, 67, 73, 74, 80, 82, 100, 128, 142, 149, 157, 162, 166
4. Team science intervention that is NOT a training program, an effort to develop new teams, or community collaboration with teams	9	2, 6, 21, 34, 53, 103, 108, 118, 146
**Example of team science** ^*^	37	9, 28, 29, 38, 39, 40, 41, 42, 44, 48, 51, 59, 60, 61, 62, 65, 69, 70, 72, 77, 90, 96, 101, 115, 120, 126, 139, 143, 144, 150, 156, 158, 164, 165, 167, 168, 170

* These are all specific examples of team science. If they included a methodological *strategy for enhancing team science*, then they are categorized according to that strategy in the section “Team science research, intervention or resource,” and do not appear under examples.

**Team science research, intervention, or resource** covered team science training and research activities, including leadership training for K-scholars and trainees, symposia to discuss best practices, and collaborative research. To foster new teams, submitters organized symposia and workshops around specific topics of interest to bring researchers together to develop ideas for new proposals. Funding opportunities developed for multidisciplinary teams took the form of awards honoring specific scientists and competitive award offerings.

**Unique examples of team science** were stories of a specific successful research team. They were coded as an “example” if they did not describe using a strategy to promote team science.

### Q6. What Translational Science Stage was Addressed?

**Fig. 1.**
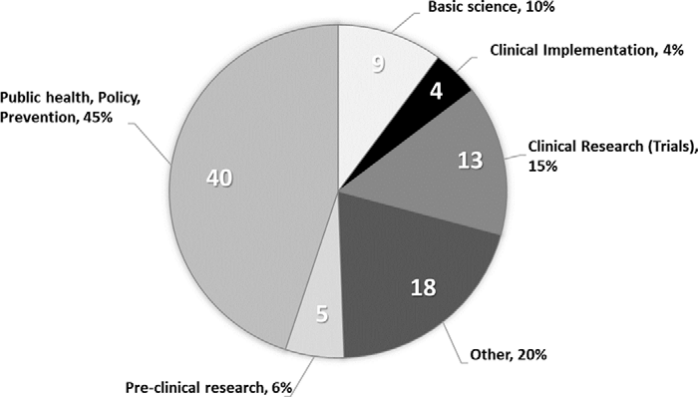
Almost half of the Great Clinical and Translational Science Award (CTSA) Team Science Contest (GTSC) submissions involved in research are in public health, policy, and prevention. This figure shows the number and percent of GTSC submissions by the translational science phase (submissions about the Science of Team Science [SciTS] are not included, *n* = 97). Number of submissions is in white. Percent of submissions are in black numbers next to the label. Some submissions involved in research were found in more than 1 TS phase: 24 submissions spanned more than 1 TS phase and 5 spanned 3 different TS phases.

The submissions were categorized by assigning them to a specific TS phase to determine if most of the team science is addressing research occurring early in the TS spectrum or later in the applying discoveries to public health [17]. A breakdown of contest submissions by TS phase is shown in Fig. 1. All the TS phases were represented in the submissions. The largest phase of TS was public health, policy, and prevention (40). Within that category, public health-related submissions made up 33 cases, followed by prevention studies (12) and finally policy studies (3). There was significant overlap, and so many submissions were a combination of categories (e.g. public health and prevention). The clinical trial phase of the TS spectrum (13) involved multidisciplinary teams studying across the lifespan and several examples show trials in the clinic as well as community-based trials. Development of extensive research networks was critical to the feasibility of many of the trials as was vital assistance from recruitment specialists. Eighteen cases did not clearly fit into the NCATS-defined phases, consisting of things such as developing a training program with shared governance or development of informatics tools to promote collaboration, enhance education, or provide valuable data repositories. Over half of the GTSC submissions did not fall explicitly on the TS spectrum, but were involved in the science of team science (SciTS, 97 cases, not shown).

### Q7. How are Hubs Using Team Science to Address the NCATS Strategic Objectives?

Supporting the CTSA program, NCATS has made team science a strategic goal with four objectives: (1) Engage patients, community members, and nonprofit organizations in the translational process; (2) Share resources through collaborative research with other NIH Institutes and Centers; (3) Form innovative collaborations with multidisciplinary scientists at other institutions; (4) Develop new collaborative structures with the private sector. Table 7 shows how the GTSC is addressing the four NCATS team science objectives. Multidisciplinary team science strategies that directly involved patients included funding multidisciplinary research for developing translational diagnostic, treatment, and disease self-management approaches. Other initiatives linked primary care service providers and researchers for clinical trials and improved healthcare. Team Science strategies involving communities included a variety of research and implementation projects: researchers and community members in joint initiatives to reduce health disparities, reduce high blood pressure, opioid abuse, improve oral health among migrants, combat cancer and rare genetic disorders. Submissions described partnerships that addressed healthy eating and obesity. In communities and state governments, academics and communities joined in programs to reduce risk factors of infant mortality, identify a rare genetic variant in newborns, promote positive parenting, and screen and identify victims of child abuse. Other collaborative partnerships focused on health promotion and wellness programs for older adults, including those in low-income housing.

**Table 7.** How are hubs using team science to address the National Center for Advancing Translational Science (NCATS) strategic objectives?

Team science objectives	Number of cases	Cases coded – by submission ID number^*^
1. Engage patients, community members, and nonprofit organizations in the translational process	45	
• Patients	11	18, 39, 42, 44, 48, 77, 133, 150, 156, 157, 165
• Community members	26	11, 14, 18, 25, 47, 54, 57, 60, 61, 64, 67, 69, 72, 73, 80, 96, 101, 104, 119, 126, 139, 142, 158, 164, 166, 167
• Nonprofit organizations	8	6, 18, 33, 54, 60, 61, 96, 126
2. Share resources through collaborative research with other NIH institutes and centers	7	9, 29, 41, 44, 52, 53, 165
3. Form innovative collaborations with multidisciplinary scientists at other institutions	21	12, 19, 21, 27, 30, 38, 48, 69, 70, 75, 97, 99, 103, 105, 106, 119, 134, 139, 163, 165, 169
4. Develop new collaborative structures with the private sector	11	18, 27, 68, 100, 103, 119, 126, 148, 161, 168

* Some submissions fulfill multiple NCATS strategic objectives for team science.

We examined the data to understand how they aligned with the second objective of NCATS, that is, sharing resources and expertise across the federal government through collaborative research with other NIH institutes and Centers. Six cases demonstrated collaboration with other NIH Institutes and Centers such as the National Heart, Lung, and Blood Institute (NHLBI), National Institute of Arthritis and Musculoskeletal and Skin Diseases (NIAMS), National Endowment for the Arts (NEA), or the National Library of Medicine (NLM). These were multidisciplinary collaborations conducting clinical trials for heart disease and testing a solution for sepsis and acute respiratory distress syndrome. The cases also represented the creation of a multicenter community-based trial in pediatrics, work integrating biomedical datasets in an effort to accelerate translational research, providing a mechanism for investigators to share data and training to improve perspective-taking skills in trainees.

Regarding the NCATS third objective of multi-institutional collaborations, several multidisciplinary submissions involved multistate and multi-CTSA hub consortia around pressing health problems like the opioid crisis, obesity, and biomarkers for autism. Several consortia formed to transform clinical research with efforts such as improving the e-consent process, expanding research into primary care practices, streamlining study start-up, and helping regional healthcare systems use the TIN. Other multi-hub efforts sought to provide a grand challenge and develop teams over a week of intensive training or to network KL2 and TL1 trainees from across multiple hubs.

Relating to the final NCATS team science objective of developing collaborative structures with the private sector, most of the submissions that involved the private sector concerned helping programs to scale inventions to help develop commercial health products or public health interventions. Other private sector collaborations involved getting industry members to join partnerships around major health challenges (e.g. opioid crisis, cancer) or getting input into potential start-up companies.

## Discussion

The value of this qualitative analysis of the GTSC dataset is the examination of the breadth of team science across the consortium. What the individual submissions lacked in detail, they made up for in the breadth of interventions, the diversity of beneficiaries, and the broad multidisciplinary involvement.

Two kinds of submissions dominated the GTSC: strategies for promoting team science, but many lacked evidence of efficacy versus examples of team science that often lacked clear team science strategies, but provided examples of successful outcomes. The outcomes included short-term evaluation metrics such as increased leadership self-efficacy, greater confidence in conducting clinical research, and greater understanding of the value of multidisciplinary research. This led to midterm behavior changes by teams such as increased efficiency, writing grants, publications, and patent applications, and developing novel apps/digital tools. Finally, several long-term outcomes were attributed to the team science strategies, including improving health access for underserved populations, expanding capacity for special populations in clinical trials, policy changes, and expanding programs to other hubs.

Team science represents the ability for teams to effectively collaborate but also, the ability to integrate knowledge from diverse perspectives. Successfully involving the community in research is one transdisciplinary challenge in team science. To partner effectively with community members involves engaging in research that is both meaningful to the community and focused on community priorities. Many GTSC submissions describe such collaborations including community interventions and improvements in public health. Several submissions were focused on joint university–community research programs to improve public- and clinical/mental-health. Other team science initiatives provided training to team scientists and community members, involving them in creating funding mechanisms for TS, reviewing project proposals, and providing venues and activities for scientists and community members to meet and develop project ideas.

Integrating knowledge from individuals who have different perspectives is another vital aspect of engaging in successful transdisciplinary team science which involves fostering knowledge sharing and optimizing the use of appropriate collaboration technologies [13]. As technology, informatics, and medicine get more advanced, more instances of knowledge integration between fields are occurring, such as pairing engineers, app developers, and medical professionals to address unfulfilled needs in medicine. Few submissions rigorously measured team science effectiveness, however, some did address the need for data access to promote knowledge integration and the need for digital tools to obtain data on the value of team science.

There is ample evidence that the CTSA consortium is addressing the team science objectives set forth by NCATS [10]. Objective two, regarding sharing resources among NIH institutes and centers, is not extensively addressed. Although some GTSC submissions mention other centers (e.g. NHLBI, FDA, NIAMS, etc.), the contest did not ask for this information specifically [10]. The other three NCATS team science objectives are well-addressed by the GTSC submissions.

The GTSC dataset provides appropriate evidence that the CTSA consortium is addressing the team science recommendations made by the National Research Council (NRC) in 2015 [13]. Here, we address the recommendations according to their intended audience. For leaders of science teams, the NRC recommends using analytic tools to guide team composition and to evaluate both professional and science leadership development for science teams [13]. Several submissions used analytic tools to guide team composition. Eight submissions were able to include evaluation results of team activities. Two submissions directly addressed the NRC’s request for translating leadership evidence by developing and assessing leadership indicators. Clear metrics for evaluating team science do exist and CTSA hubs engaged in promoting team science would be wise to carefully evaluate what works to enhance team science [13, 18–20].

Concerning geographically dispersed teams, the NRC recommends providing activities to develop shared vocabularies and work routines, train team members to use proven collaboration technologies, and develop criteria to give credit for team-based work [13]. Many of the submissions involved training in team-based competencies and developing common languages for collaboration. Many hubs developed new informatics tools to improve collaboration across geographic distances.

Regarding public and private funders, the NRC recommends reducing the barriers to team science by encouraging new collaborative models, providing informational resources, and removing disincentives to participate in team science while including collaboration plans and how they will promote knowledge integration between disciplines. Finally, they propose studying the effectiveness of team science and facilitating access to research and personnel data [13]. Many GTSC submissions described highly unique methods to promote collaboration between disparate groups of researchers. Only three submissions addressed overcoming academic disincentives to engage in team science by changing the P&T policies to allow team scientists to achieve parity with individual investigators. Some submissions addressed developing written plans for the collaboration. Team charters were regularly mentioned as one of the most useful collaboration tools.

Pertaining to researchers, the NRC recommends improved methods to match participants with project needs to enhance team effectiveness, professional and leadership development for team science, and collaboration with universities to develop new principles and remove barriers that discourage team science. Several submissions promoted matchmaking among scientists from different disciplines around research needs in basic science, healthcare, and public health. Likewise, many of the submissions involved professional development covering team science competencies. The final recommendation, to evaluate how criteria for allocating credit for team science are working was not addressed by the GTSC submissions. This may be due to the fact that although most CTSA hubs have some policies expressing the value of team science, there is significant variability across the consortium and within-institution variability as to how the policies are carried out [21]. Most hubs have not had sufficient time or resources to evaluate the effects of team science P&T policies.

In connection with universities and other scientific organizations as well as the scientific community, the NRC recommends fostering positive team processes to enhance effectiveness, create and evaluate leadership development opportunities and remove barriers that discourage young faculty who are interested in team science from joining teams. The GTSC received many examples of positive team processes from training in team competencies to fostering good communication and strong leadership. The final barrier preventing systemic adoption of team science may involve diverging from the current funding system that rewards the investigator-initiated grants to provide additional incentives for team-based science (e.g., P&T policies for team science). In summary, both the NCATS team science objectives and most of the NRC recommendations for team science are being addressed in the GTSC dataset. There is one notable exception: evaluating how criteria for allocating credit for team science are working was not addressed. This area warrants further research.

### Strengths, Limitations, and Future Directions

Strengths of this study include the robust CTSA consortium participation of 45/64 (70%) hubs and the large and extremely diverse dataset of submissions to the GTSC, which enabled the demonstration of the great heterogeneity in the consortium interventions to enhance team science. The contest provided more team science efforts than would other methods such as expert opinions, focus groups, or surveys. The resulting dataset and qualitative analysis provide hubs with a large reservoir of information so that they can focus on the team science strategies and outcomes that interest them. These strategies show the great breadth of innovation, particularly in interventions that involve the community or industry in team science. Besides many traditional team science trainings, there were several submissions that encompassed both an intervention, such as a scientific retreat, and a successful example of team science. Although the GTSC did not request examples of innovative and successful team science, several were submitted that demonstrated involvement of researchers who are not typically associated with team science.

Subdividing submissions according to the 2018 NCATS FOA categories assumed that a particular CTSA component was the driver of the team science story. This was not the case in many submissions that either did not specify what component was doing the work or involved many collaborating CTSA components and/or institutional promotion of team science. This is a positive finding which suggests team science has permeated so many aspects of the CTSA infrastructure that it has become intrinsic to the fabric of a CTSA hub and is no longer an activity performed by a single core.

For limitations, this study is a secondary analysis of an existing dataset. The GTSC dataset is not a random sample nor is it representative of all team science, and not all cases in the dataset were equally informative. The GTSC was designed to be an easy, low-effort contest so that more hubs would respond to the survey. Considering potential survey fatigue, the GTSC designers had to make a choice between getting fewer entries that were more detailed versus getting more responses that were very brief. They opted for the latter to demonstrate the breadth of team science. Additionally, some entrants submitted a specific example of a successful team rather than strategies for encouraging better team science, suggesting perhaps a lack of understanding about the purpose of the GTSC. Thus, some lack of clarity in describing interventions was due to the 150-word limit, which meant the submissions lacked significant detail on the process, the key stakeholders, or the evaluation outcomes. An additional limitation is a difficulty in teasing out distinct categories when there is significant complexity between the causal networks required to move from early TS training to measuring TS improvements to more productive research and publications to health-related benefits.

There are many challenges for defining team science outcome metrics, as well as institutional policies that could thwart well-meaning efforts including P&T criteria, indirect cost allocation policies, team science incentives, and the availability of collaborative organizational structures. It is more feasible to evaluate the knowledge, attitudes, and beliefs about team science functioning. It is much more challenging to evaluate downstream evidence that teams are functioning better or having real impacts. Only a few submissions described evaluating their team science strategies to encourage team approaches, reduce barriers to team science or improve team functioning. None of the submissions examined how credit for team contributions was allocated, which poses a significant challenge that deserves more attention in future studies. Only one submission described using control and experimental groups, emphasizing how difficult such approaches can be. Although some of the team science examples did not provide team science strategies, they did provide some more distal team science outcomes. These included changes in health policy, expanding the team science program beyond the hub, better health, screening, and access for underserved populations, and increased efficiency.

In conclusion, analysis of the GTSC dataset shows that the CTSA consortium is involved in an extremely diverse array of team science activities, which are appropriately addressing most of the team science recommendations made in 2015 by the NRC and the NCATS team science objectives. The strategies in this paper provide a resource for CTSA hubs seeking to expand their team science endeavors. To use this information, hubs would match their team science goal with our research questions. For example, if a hub wants to expand team science to include the community. Three of the research questions deal with the community as beneficiaries (Q3 and Table 3), getting the community involved in research (Q4 and Tables 4 and 5), or fostering community collaborations with teams (Q5 and Table 6). Once hubs locate examples, they can cross-reference those hubs with the list of contestants in Appendix 2 and contact those hubs to learn from their experiences to get assistance in their team science expansion. Future research should focus on evaluating the efficacy of team science strategies.

## Supplementary material

For supplementary material accompanying this paper visit https://doi.org/10.1017/cts.2021.812. click here to view supplementary material
