# Supplementary material for: What does team science look like across the CTSA consortium? A qualitative analysis of the Great CTSA Team Science Contest submissions
Source: J Clin Transl Sci. 2021 Jul 12;5(1):e154. doi: 10.1017/cts.2021.812 (PMC8411266; doi:10.1017/cts.2021.812)
Supplement: Supplementary file 1 [file S2059866121008128sup.zip › S2059866121008128sup001.pdf]

ID	Hub	Headline	Paragraph
1	DUKE UNIVERSITY	Enhancing Our Workforce Competency by Competency to Create Stronger Teams	Researchers engaged in team science include not just interdisciplinary research faculty, but also well-trained staff. Identifying and nurturing the strengths of research staff has been under-valued in clinical research. Duke's School of Medicine recognized this issue and sought to restructure and empower its research staff, so they may better serve their critical role on collaborative research teams. The Workforce Engagement and Resilience (WE-R) initiative, supported by Duke's CTSA, is a broad-reaching effort that includes a competency-based framework to encourage staff to gain competencies in key research domains. Using the framework ensures consistency across clinical research professionals and promotes career growth using objective assessments. The framework fosters strong research staff that can provide high-quality support for research teams. By raising the value of our research staff, they can better contribute their strengths to research collaborations. In addition to encouraging team science principles, the initiative helps develop and retain our workforce.
2	UNIVERSITY OF ROCHESTER	Team Science Meetings Debriefing	The Team Science function for our CTSI is one of seven functions within our Population Health Branch. Population Health is one of six, newly formed branches within our CTSI. The Population Health branch includes investigators and staff from different disciplines representing the different functions. During our recent meetings devoted to establishing branch level goals, team members within our branch agreed to reserve the last two minutes of every meeting for a debrief. Debriefs are widely used in aviation, defense, and some sectors of health care for improving team performance. Debriefs are less commonly used in team science. Our debriefs address both team process during the meeting and meeting outcomes. We discuss what we went well, where can improve, and how we might improve. Our experience suggests that debriefs not only help us to track whether we achieved meeting goals, but also to discuss the meeting process including acknowledging each other and making sure everyone has an opportunity to contribute.
3	UNIVERSITY OF TEXAS MED BR GALVESTON	First Grounded Transformational Leadership Theory for Translational Science Developed and Creates Behavioral Exemplars for Implementation	Responding to the National Research Council's (2015) team science report, team science staff at UTMB developed and implemented a contextualized leadership training program for scientific leaders. Using the Kouzes and Posner (2012) Leadership Challenge Program, a multi-year (2016-2017) leadership intervention has involved behaviorally based 360 assessments, monthly classes using a flipped classroom and constructivist design, followed by a community of practice format. Utilizing electronic and face to face mediation, a class of experienced PIs and trainees generated behavior specific exemplars of the ten leadership commitments espoused by the Leadership Challenge model. In all, thirty behavioral exemplars were reported at the 2018 Science of Team Science Conference, focusing upon the utility of using team science specific examples in developing scientific leaders. This effort has generated the first ever contextually grounded leadership theory for scientists, and is now being implemented by two other CTSAs and by the Pepper Older American Independence Centers.

ID	Hub	Headline	Paragraph
4	UNIVERSITY OF TEXAS MED BR GALVESTON	First Leadership Assessment Center for Translational Scientists Significantly Influences Career Perceptions	In 2018, team scientists, evaluators, and consultants at UTMB developed and implemented the first Leadership Assessment Center (AC) for translational science. Following the National Research Council's request for translating leadership evidence, twenty-five specific evidence-based competencies were developed involving seven dimensions of leadership (meeting management, transformational leadership, innovation, adaptation, interpersonal skills/communication, and project management). Twenty-one trainees and scholars (KL2, TL1) participated in a two-day AC involving a multi-trait multi-method design involving 360° surveys, case analyses, in-basket exercises, role plays, and team presentations. Experienced actors were used for behavioral simulations, and trained evaluators were used to score each participant, followed by group feedback and individual development planning. The results were presented at the 2018 Science of Team Science Conference, indicating that all six paired comparison (pre-post) t-test involving self-efficacy measures were significant (p < .0001), and perceptions of methodological/criterion validity, overall AC satisfaction, and anticipated impact upon participant careers were very favorable.
5	UNIVERSITY OF TEXAS MED BR GALVESTON	Teaching Team Science and Leadership within the Context of Research Design Changes Student Self - Efficacy	An eight-week translational research study design course that introduces pre-doctoral students to team science competencies in the context of developing a translational research proposal has been facilitated since 2016 by senior faculty associated with the UTMB CTSA. Student teams developed an NIH-style grant proposal on a translational research topic to practice team science competencies (e.g., advocating for multiple points of view, demonstrating group decision-making techniques, developing a team contract, managing conflict, and engaging experts from different disciplines). A leadership workshop (Journal of Clinical and Translational Sciences, 2018) was embedded in this course, and students were introduced to skills on how to recognize and manage their own leadership competencies. Using a modified Kanes-Bates (1998) leadership self-efficacy survey (pre-and post-training repeated t-test, p=0.04), students reported a significant improvement in leadership confidence (e.g., team leadership skills, taking charge, etc.) as it relates to interprofessional collaboration in a biomedical research project.
6	UNIVERSITY OF TEXAS MED BR GALVESTON	Medical School Hosts International Team Science Conference and Provides Leadership for the Team Science Field	In May 2018, the UTMB CTSA hosted the prestigious international Science of Team Science (SciTS) Conference, at which 214 attendees from more than 20 research fields attended sessions and panels involving research across many domains of team science and participated in workshops addressing team science issues. During this conference, UTMB and CTSA experts facilitated a CTSA-specific team science needs assessment and provided workshops tailored to investigators and CTSA administrators involving evidence-based best practices in leadership and team creativity. Additionally, UTMB established a formal relationship between the SciTS community and the Journal of Applied Behavioral Science, resulting in forthcoming publication of the study chosen as the conference's Best Paper, authored by a UTMB visual analytics scholar. UTMB also provided significant financial and staff support for the establishment of the International Network for the Science of Team Science (INSciTS), which will serve the team science community as a 501(c) educational organization.

ID	Hub	Headline	Paragraph
7	UNIVERSITY OF TEXAS MED BR GALVESTON	Mixed Methods Model to Evaluate Scientific Teams Identifies Most Innovative Teams	Team scientists and evaluators at UTMB developed and published (Evaluation and the Health Professions, 2014) a much-needed model to assess and evaluate Multidisciplinary Translational Teams. Using a mixed methods approach involving artifacts (applications, reports), bibliographics, meeting notes, milestone successes, team surveys, and process observations (structured observations), teams were rated by expert panels using 8 criteria involving: 1) research and scientific progress, and 2) team maturation and development. Teams were categorized as 1) early in development, 2) traditional, 3) process focused, or 4) exemplary based on their position on a 2 x 2 grid, and their developmental progress over time illustrated. Using the same 8 overall evaluative criteria, continued evaluation of the teams (Journal of Clinical and Translational Science, 2016) illustrates how team trajectories that increase productivity and innovation (publishing outside of traditional disciplinary journals) can be mapped, thus showing the importance of multiple disciplines and transformational leadership in specific.
8	UNIVERSITY OF WISCONSIN-MADISON	A model for shared decision making via team science	ICTR promotes team science through leveraging the resources of the Wisconsin Research and Education Network (WREN), a practice-based research network supported by CTSA core funding. WREN pursues pragmatic, primary care-focused research in partnership with clinics across the state. In 2015 WREN tasked academic, clinician, and community stakeholders with developing a research agenda to improve chronic disease management and treatment. One resulting directive was to increase shared decision making (SDM) implementation within primary care clinics. In response to this directive, WREN launched a project in 2017 to improve SDM for lung and breast cancer screening. A SDM training workshop for primary care providers and a streamlined clinic workflow were developed by a diverse team of interdisciplinary academicians, clinicians, and community members. Our SDM training workshop offers a model for enhancing shared decision making via diverse stakeholders involved in team science.
9	MEDICAL UNIVERSITY OF SOUTH CAROLINA	A Successful South Carolina Statewide, Cross-disciplinary Team Science Story: Implications for FDA policy development and smoking-related health disparities	Research is needed to help the Food and Drug Administration (FDA) select effective health warning labels (HWLs) for cigarettes. The South Carolina Clinical and Translational Research Institute held a scientific retreat on tobacco-related research, focusing on FDA-relevant topics. As a result, a new cross-disciplinary team of health communication specialists, clinical researchers, and epidemiologists from University of South Carolina and Medical University of South Carolina was formed and came up with the novel idea of examining how adult smokers in low- and middle-income areas of South Carolina respond to HWLs with different content, including pictures. This study found that pictorial HWLs with graphic images have the most pronounced effects on smokers, including smokers from groups that have been hard to reach. This collaborative study led to extramural grant funding (R01, $2.7 million) and has yielded 33 publications in high-impact journals and has had clear impacts on HWL policies around the world.

ID	Hub	Headline	Paragraph
10	UNIVERSITY OF KENTUCKY	A Team Approach to Developing a Data Science Graduate Degree	The University of Kentucky is a University system with the medical campus and the main university campus collocated, simplifying collaboration across the full range of University programs. The UK CCTS used this advantage to develop a campus-wide Masters in Data Science degree program by supporting a collaboration among faculty members in the departments/units of Computer Science (College of Engineering, BMI Co-I Mirek Truszczynski), Biostatistics (College of Public Health, BERD Core Director Heather Bush), and Biomedical Informatics (College of Medicine, BMI Co-director Hunter Moseley). This is the first graduate degree of this kind in Kentucky that uses SHARED governance across three Colleges to provide comprehensive training and development for a new generation of data science professionals grounded in fundamentals across three disciplines that also leverages the tremendous CCTS educational resources (seminars and courses) offered in regulatory, ethics, and responsible conduct of research, as well as biostatistics and biomedical informatics research experiences related to human health.
11	YALE UNIVERSITY	A Team-Based Model to Improve Child Abuse and Neglect Recognition in Community Emergency Departments	Emergency Department (ED) providers may fail to recognize or report child abuse and/or neglect (CAN). The majority of children in the U.S. who seek care in EDs are seen in community EDs, where abusive injuries may be missed more frequently compared to pediatric EDs. To improve recognition and reporting of CAN, Yale Assistant Professor Gunjan Tiyyagura, MD designed the Community ED CAN Program, through which ED clinicians (physicians and nurses) team up with CAN experts (pediatricians) and child protective services staff (social workers and others). The team collaborates to train designated clinician advocates. Then, on an ongoing basis, clinician advocates consult with other ED clinicians in suspected CAN cases, facilitate bi-monthly multidisciplinary case conferences, and provide case-based education to other ED staff (further expanding the team). This team-based intervention, implemented at two area hospitals, is under evaluation now.
12	UNIVERSITY OF WASHINGTON	Academic-Clinical-Community Research Partnerships: Multifaceted Approaches to Confronting the Opioid Crisis	Novel, innovative approaches to address the growing national opioid crisis are needed, and collaboration across diverse stakeholders is vital. The ITHS leveraged its extensive networks of research, clinical and community partners to brainstorm innovative ideas and facilitate new research collaborations by hosting a series of interactive in-person and virtual sessions. We held three sessions in Seattle, and a virtual session across Washington, Wyoming, Alaska, Montana and Idaho. Sessions utilized team-engaging tools to promote collaboration. 26 researchers, clinicians, staff and community representatives described their research, experience with opioid and other substance use disorders, outlined the needs of their communities, and shared new project ideas. From this effort, new research teams are developing and preparing applications. Examples of resulting collaborations include a data science project examining opioid use disorder (OUD) treatment in primary care and another tailoring an OUD treatment model to the unique conditions in rural healthcare.

ID	Hub	Headline	Paragraph
13	UNIVERSITY OF CINCINNATI	Accepting a Higher Power – Management vs Leadership	You may not be at Sunday's sermon, but listen up. Leadership and Management have specific characteristics and roles on teams (as well as organizations – aka bigger teams). Without understanding and defining both on teams, team members can become frustrated. We also don't want to put all our eggs in one basket, do we? This can lead to more of the same traditional thinking. Did we forget about multi-generational and multi-cultural expectations?! Generating a team charter to outline roles will help managers do and leaders innovate, allowing for other team members to do what they do best. University of Cincinnati Academic Health Center research stakeholders and healthcare practitioners and educators were invited and attended the Leading Effective Teams: Team Science Workshop, held in Spring 2018 by Team Science facilitator experts in their fields. Lessons learned: needs more discussion on how to be successful; and too much information for one workshop!
14	JOHNS HOPKINS UNIVERSITY	Advancing Social Justice through a Multidisciplinary /Multisector Partnership: The Johns Hopkins Center for Health Equity	The Johns Hopkins Center for Health Equity envisions a world in which all people can achieve their best health. Our approach integrates transdisciplinary research, community activation, education, and policy translation. It entails meaningful involvement from multiple stakeholders. We use data to define the problem and its causes. Then, we design, test, and implement interventions, engaging patients, clinicians, researchers, health system and community leaders, and policymakers every step of the way. Our academic-community partnership is founded on respect and collaboration. Since 2010, we established an active community advisory board, completed three studies identifying effective interventions, trained 70 health equity researchers, developed educational programs for health system leaders and community health workers, successfully advocated for legislation to reduce health disparities, and secured an additional $15M in funding. Our Center strives to make healthcare institutions more equitable, communities more engaged, and health policies and practices more effective at eliminating health disparities.
15	WAKE FOREST UNIVERSITY HEALTH SCIENCES	Advancing Team Science AND Faculty Recruitment through Novel Research Studios	New faculty members being recruited to an institution are often unfamiliar with potential collaborators who could provide an interdisciplinary approach to their research. Recognizing the need for advancing team science while enhancing recruitment efforts, the Wake Forest CTSI Team Science Program initiated a Research Studio model to introduce potential new faculty to current faculty from different disciplines, but who have an interest in the recruit's research. The 90-minute moderated sessions are offered during the 2nd or 3rd interview visit for the potential faculty member. Working with the Department or Center recruiting the new investigator, the Team Science Program identifies others outside the Department/Center with relevant expertise and interest who are likely to contribute innovative ideas and potential solutions to the recruit's scientific problem. To date, this new service has been used for two faculty recruits and resulted in the formation of a new team focused on food insecurity research.

ID	Hub	Headline	Paragraph
16	YALE UNIVERSITY	African American Churches and Yale Partner to Tackle Depression and Social Determinants of Mental Health	Depression is the leading cause of global disability. Although evidence-based treatments for depression exist, studies suggest that ethnic minorities who are potentially diagnosable with depression neither seek nor receive adequate care. This disparity may be due to problems of access, resource inequalities, and sociocultural factors, including differences in worldview, systemic racial bias, and stigma. A project led by KL2 Scholar Miraj Desai, PhD at Yale is seeking to address this impasse—between persons of color left suffering in the community and systems-level barriers—through an innovative participatory research approach that involves directly partnering with the local African American faith community. Together, they are working to collaboratively identify pathways to the development of depressive symptoms (considering both personal and social determinants), as well as possible junctures for community-based early interventions. This culturally-responsive, team-based research will not only impact community well-being but should also expand options available within the healthcare system itself.
17	MEDICAL UNIVERSITY OF SOUTH CAROLINA	An Interprofessional Student Program for Collaborative Community Engagement and Solving the Healthcare Puzzle	The Dr. Raymond S. Greenberg Presidential Scholars Program is a two-semester extracurricular experience that brings together 50 students from the Medical University of South Carolina Colleges of Dental Medicine, Graduate Studies, Health Professions, Medicine, Nursing, Pharmacy; and Charleston School of Law. Students explore social, political, and human issues to healthcare professionals and biomedical researchers that transcend disciplines and professional boundaries. The program aims to enrich the academic environment through meaningful interprofessional student interactions with the community. Based on the scholars' areas of interest, five teams emerge. Faculty scholars from each College mentor the teams who collaborate with a community partner on a community responsive project related to a universal healthcare-related theme. These projects are the capstone activity of the program in which each team develops, assesses and implements their work with the community partner. Students develop skills to expand their understanding of linkages/relationships with complexities of health and healthcare delivery.
18	MAYO CLINIC ROCHESTER	Area stakeholders build network for promoting health and continuous learning	CTSA-funded researchers initiated a mixed methods participatory research project in 2014 to understand barriers to scaling and clinically integrating evidence-based health promotion programs in the community (e.g. programs for diabetes prevention, chronic disease management, falls prevention, etc). From the outset, clinicians, researchers, patients, community leaders, aging service providers, public health, philanthropy, and a software developer were involved. Insights were translated into a scalable model and accompanying technology for building community capacity to deliver programs and integrate these with healthcare. Initial implementation resulted in an network of >20 community-based organizations providing programs. The network is overseen by a multi-sector steering committee and participates in continuous data collection and research. The technology has facilitated clinical referrals to the programs across multiple health systems. In 2017, >1000 individuals were reached. The model, technology, and accompanying resources are now being used or considered by states, metropolitan areas, and health systems around the country.

ID	Hub	Headline	Paragraph
19	MEDICAL UNIVERSITY OF SOUTH CAROLINA	Assessing the Bite Counter as a Tool for Weight Loss - A Successful South Carolina Statewide, Cross-disciplinary Team Science Story	Cross-disciplinary team members of Clemson University's Human Factors and Ergonomics Research Institute and the Medical University of South Carolina (MUSC) Weight Management Center have worked together to investigate the use of a "Bite Counter" as a tool for weight loss. This is a collaboration that resulted from MUSC's CTSA-held mHealth Technology and Obesity Scientific Retreats, further enhanced by subsequent MUSC CTSA pilot project funding to the team. The goal of the collaborative work is to examine if reducing bite count leads to reduced caloric intake. As a novel tool to objectively monitor intake in humans, the Bite Counter could be an essential element to advance obesity research and clinical care. The Bite Counter may enable new research studies to be undertaken and novel weight loss strategies to be developed. The team has been successful in receiving Small Business Technology Transfer grants to further develop the bite counter device.
20	INDIANA UNIV-PURDUE UNIV AT INDIANAPOLIS	Blazing the trail for community-engaged research and team science	The Trailblazer Award of Community Health Partnerships brings together faculty and community members on projects that target health equity. We support teams financially and with resources leveraging team science. The projects kick-off with an orientation connecting them to Indiana CTSI's regulatory, biostatistics, bioethics and financial programs. Each project team is matched with a "buddy" from the Community Health Partnerships Advisory Board. These buddies are community or university based leaders who serve as mentors to the project teams. As the teams near completion of their projects we host a communication workshop led by visual and service design experts focused on public understanding of science and dissemination of their results. Awardees are offered guidance from the Indiana CTSI's ICReATE program on securing future funding for the next phases of their work together. The Trailblazer Award is a successful program with around 30 applications and 8 project teams completing projects per year.
21	YALE UNIVERSITY	Bringing biomarkers to autism clinical trials	Autism is a prevalent neurodevelopmental disorder affecting as many in 1 in 68 children in the US. Clinical research and practice remain reliant on subjective clinical assessment. The Yale Hub is supporting Dr. James McPartland's development of the first network designed to develop objective biomarkers for application in clinical trials. Yale's hub was instrumental in designing the grant and the application, and its staff serve as the Project Management team overseeing function of the Autism Biomarkers Consortium for Clinical Trials and coordinating activities across five sites, including four other hubs at UCLA, University of Washington, Duke, and Children's Hospital Boston. Yale's hub also provides the Data Coordinating Center to develop and maintain a secure data infrastructure and to monitor adherence to GCP standards. Thanks to the support of Yale's hub this consortium has been highly successful in meeting its scientific objectives.
22	UNIVERSITY OF SOUTHERN CALIFORNIA	Building Bridges: Assessing Team Science Readiness	The SC CTSI retained collaboration expert, Dr. Judith Olson, to guide the SC CTSI in adopting her Collaboration Success Wizard which focuses on understanding distance collaboration. We have successfully implemented Phase One of our multi-stage project which offered the Collaboration Success Wizard to current SC CTSI pilot and team-building awarded teams. The Collaboration Success Wizard was used by 10 research teams to assess factors that have the potential to strengthen or weaken their ability to collaborate effectively in their team-based research. Dr. Olson provided both personal and project-level reports to help the teams launch successful and productive collaborative projects. Based on user feedback from the Phase One participants, SC CTSI and Dr. Olson are working together to tailor the Collaboration Success Wizard for a collaboration assessment tool more directly oriented to translational and clinical research teams for a Phase Two offering.

ID	Hub	Headline	Paragraph
23	UNIVERSITY OF SOUTHERN CALIFORNIA	Building Bridges: facilitating research collaboration	The SC CTSI has launched a team building facilitation service for researchers interested in forming multidisciplinary research teams through our team-building voucher. This facilitation service provides: 1) training for investigators on best practices in team science principles based on NCI's "Collaboration and Team Science Field Guide"; 2) facilitated brainstorming meetings based on NSF funded "BRIDGES" program concept and; 3) recommendations on running future effective brainstorming meetings. Feedback from participants in the facilitated brainstorming meetings were positive. Comments included: "We are guided which helps a lot, at least as a young investigator" and "Was extremely helpful in getting people's thoughts organized and helping generate a 'big picture' view of the research project". Comments from the workshop to postdocs for running effective meetings included: "I loved this workshop! It was so much fun and interactive...... It is a lot of valuable information for trainees. Also I think people enjoyed it."
24	UNIVERSITY OF CINCINNATI	Building Team Skills When You're Not Looking: A Natural Experiment	We offer many workshops and seminars on the application of team science principles. The episodic nature of these experiences requires us to use exercises that "simulate" the natural development of teams and team skills. The learners recognize that what they are seeing and doing is "not the real world." To give learners a "real" experience in team building, we built a natural experiment into a semester course on the science of team science. As part of each class, we inserted a 5-minute "warm-up" question/discussion in which the students disclosed small things about themselves. Over the semester the questions became more personal. Students were initially guarded but became more comfortable over time. On the final exam, half of the questions related to the "disclosures." Students scored 100% on those questions. They admitted that their disclosures at the end of the semester would have been too uncomfortable to discuss initially.
25	COLUMBIA UNIVERSITY HEALTH SCIENCES	Care for Rare: Bringing Together Families, Clinicians and Researchers	During summer 2018, three groups of patients from New York and New Jersey with rare genetic disorders, their families, clinicians, and researchers met for the first time to understand the mutations in the genes HNRNPH2, ASXL3, and CSNK2A1. Facilitated by the Precision Medicine Resource (PMR) at the Irving Institute for Clinical and Translational Research, home to Columbia University's CTSA Program hub, the families were able to share their experiences and learn from each other. Clinicians and scientists shared their knowledge with the families and learned about the clinical variability and natural history of these disorders, leading to a greater appreciation of the families' priorities and potential knowledge gaps. Researchers were able to collect medical records and perform EEGs, neuropsychological evaluations, and collect blood samples and skin biopsies to assess biomarkers and make cell lines to study these genetic conditions. Experiments to understand the molecular bases of these rare diseases will inform future therapeutic strategies.
26	EMORY UNIVERSITY	Celebrate Team Science	In an effort to demonstrate that our institutions value, recognize and reward team science, the Georgia CTSA has established the Presidents' Award of Distinction for Team Science and the Team Science Award of Distinction for Early Stage Research Teams to reward and promote excellence in multi-disciplinary research teams within the Georgia CTSA. The call for nominations will be sent this fall and the PIs will use the criteria established by the CMDTS steering team to select the winner(s). The winning teams will receive a monetary award for continued research and will receive a special invitation to present at the 2019 CTSA Georgia Statewide Conference. The award is conferred by the presidents of the Georgia CTSA institutions.

ID	Hub	Headline	Paragraph
27	EMORY UNIVERSITY	Chat Fast! Meet new collaborators at Speed Networking	Speed Networking consists of an evening of energetic and dynamic networking between researchers across the Georgia CTSA focused on a broad topic. Attendees participate in a series of short one-on-one meetings with potential collaborators from both clinical and basic science areas. Participants have the opportunity to reconnect during a reception that follows. A Speed Networking event on the Opioid Epidemic was held in Spring 2018 at Emory University. The event consisted of a diverse audience, inclusive of 3 Georgia CTSA affiliates, 2 outside academic institutions, and 3 industry contacts. All attendees rated their experience highly and left the event with at least one potential collaborator. During the 3-month follow-up, all respondents reported that their participation had resulted in at least 1 new collaboration and 1/3 anticipate future academic outputs as a result.
28	UNIVERSITY OF WASHINGTON	Collaborative Development of a cohort discovery tool, Leaf	ITHS Biomedical Informatics used a customer-focused, team-based approach in the development of our self-service cohort discovery tool, Leaf. This approach involved weekly sprints, followed by design “huddles” with a group of future users from UW Medicine Cardiology, Infectious Disease, and other disciplines. Each huddle included an extensive idea and design wish input session. Within 5 months we had a product that was available for a larger pilot release within UW Medicine. Because the design focused on user input, the tool spoke to the inherent thought process of the researcher and included the features that they found most valuable. This process required clear communication skills- forcing us out of our “programmer-speak” and forcing the users to be clear in their clinical-speak. Clarifying meaning and intent became a critical part of the process. Leaf was available for use as pilot tool in record time, and has recently become an institutionally-supported production tool.
29	VCU	Collaborative research identifies new potential therapy for heart failure and acute myocardial infarction	During the past 5 years, Antonio Abbate, M.D., Ph.D. (VCU Pauley Heart Center), and Benjamin Van Tassell, Pharm.D., (School of Pharmacy) serve as co-principal investigators on the four clinical trials supported by the National Heart, Lung, and Blood Institute. They have assembled a multidisciplinary team composed of cardiologists, pharmacists, nurses, exercise physiologists, nutritionists and basic science researchers to address the question of whether blocking Interleukin-1 (IL-1), an apical pro-inflammatory cytokine, quenched the systemic inflammatory response associated with heart failure and AMI, resulting in improved outcome. The hypothesis that anakinra would be beneficial derived from preclinical data also generated at VCU. While numerous medications are approved for the treatment of heart failure and AMI, there are no approved treatments that specifically target inflammation. The initial data from the first 2 completed studies showed improved exercise capacity, and reduced adverse clinical events, in patients with systolic or diastolic heart failure.

ID	Hub	Headline	Paragraph
30	MEDICAL UNIVERSITY OF SOUTH CAROLINA	Collaborative Team of Specialists Working Together to Manage Back Pain in Rural Communities via Telehealth	Access to cross-disciplinary specialists to manage pain care in rural communities is poor. Barriers to access include institutional, financial (lack of insurance, underinsurance, inability to afford copays), logistical, poor mobility, geographic isolation and transportation limitations particularly with extended travel distances, and legal concerns. To overcome the barriers, a team of physical therapists, behavioral health and interventional pain management experts, was formed as a result of a South Carolina Clinical and Translational Research Institute held scientific retreat on pain. The team was subsequently funded by the SCTR pilot project program to study the convenience and effectiveness of using video teleconferencing, remote evidence based group supportive therapy program, and patient satisfaction. Patients and their primary care providers should benefit from this team recommendations regardless of they participate in the telehealth program. The team received a successful Duke Endowment Award for 3 years to evaluate a new Pain Rehabilitation Program at Medical University of South Carolina.
31	UNIVERSITY OF FLORIDA	Colleagues you may know: Using social networks to test personalized pilot opportunities	Most of us have used “People you may know” suggestions from Facebook and LinkedIn to reconnect with friends. What if we could use network algorithms like these to strengthen research communities, jump-starting collaborations and new research thrusts? Our CTSI’s first experiment with network-guided pilot awards seeks to do just that. Using publication and grant data, we mapped the university’s collaboration network and developed algorithms to find research communities within the network. Beyond simply describing the network, we developed a pilot intervention to invite and incentivize 15 pairs of scientists to propose a joint project. Each pair included researchers who had not published or received a grant together but belonged to the same network community, and whose collaboration would maximize community cohesion. Three projects were funded. Does it work? Time will tell: Through statistical methods used for randomized trials, we will assess whether the intervention helped accelerate high-impact translational research.
32	COLUMBIA UNIVERSITY HEALTH SCIENCES	Columbia University Scientific Profiles (CUSP): A Novel Team-Science Platform	The Columbia University Scientific Profiles (CUSP) is an innovative expert search platform for facilitating team science. It allows anyone from anywhere to perform name or keyword searches for experts with publications or grants on any research topic at Columbia University Irving Medical Center. It uses modern Semantic Web technologies for investigator-centered information extraction and integration from a variety of databases for human resources, publications, and funded research projects. It provides robust name disambiguation for monthly publication updates using PubMed. CUSP is instrumental to team science efforts at Columbia. Between 11/1/2015 and 8/31/2018, CUSP served >10,000 sessions for 8500 unique visitors, with an average of 255 active users using it on a monthly basis. It is used to support campus-wide collaborative team science initiatives such as a University-wide obesity workshop, the annual (2016-2018) precision medicine symposium, and the 2018 opioid crisis symposium.

ID	Hub	Headline	Paragraph
33	PENNSYLVANIA STATE UNIV HERSHEY MED CTR	Community Engagement Studios: Tackling issues together	Penn State CTSI's Community Engagement Studios are two-hour sessions of eight-to-twelve community experts that give a researcher community input in the early phases of developing a proposal. Dr. Deepa Sekhar used the studio to evaluate her proposal to address rising adolescent depression and suicide rates in partnership with schools. A team that involved adolescents, parents, school staff, the leaders of two mental health and suicide prevention organizations, a behavioral health managed care company representative, and a suicide prevention grant project director worked with the Penn State research team through the studio process to strengthen Sekhar's project. Through this team approach, Sekhar's project successfully received funding from the Health Resources and Services Administration in May 2018 and she hopes to hear about a related opportunity at the end of the summer. The Community Engagement Studio continues to be expanded and fine-tuned to promote a more effective team approach to study design.
34	EMORY UNIVERSITY	Conference Highlights and Celebrates Clinical Science Across the State	This annual clinical science conference showcases the Georgia CTSA and provides opportunities for attendees to network with national leaders and NIH staff in clinical and translational science and education. The conference consists of keynote presentations, oral abstract talks and poster sessions, along with informational sessions and networking opportunities. Over 200 attendees, from academic institutions across the state, had the opportunity to share their research and develop new collaborations at the 2018 conference. Planning is underway for the 2019 conference, which will offer a new meeting format and even more opportunities for collaboration and networking. We hope to expand in 2020 to neighboring hubs.
35	BOSTON UNIVERSITY MEDICAL CAMPUS	Connecting Investigators and Trainees at Boston University (BU) Through Affinity Research Collaboratives and Bridge BUilders	BU CTSI Team Science leader, Dr. Katya Ravid and colleagues developed in 2010 innovative research structures we termed – Affinity Research Collaboratives (ARCs), consisting of 7-15 investigators from different departments and disciplines, with a common interest in developing a biomedical research area that the faculty identified ("bottom up" approach). Importantly, ARCs mandate the inclusion of trainees, providing hands on experience in Team Science. A prominent example includes a Bridge BUilders initiative overseen by Dr. Jonathan Rosen by which Bioengineering students develop technology/devices based on clinical needs identified by biomedical faculty. ARCs are co-funded by the CTSI for up to three years pending yearly reviews based on metrics, including interdisciplinarity and innovation. To date, 12 ARCs have produced 421 co-publications and 123 NIH grants (at a 55% success rate), in addition to developing new university capacity, including new research programs, a regenerative medicine center, and two graduate programs, as published in pubmed/28445220.

ID	Hub	Headline	Paragraph
36	CHILDREN'S RESEARCH INSTITUTE	Creating a Culture of Collaboration at George Washington University (C3@GWU)	C3@GWU convenes a cross-disciplinary community of expert faculty interested in issues related to scientific collaboration and connects them with regional and national partners and experts to explore and address topics that foster collaborative science and generates short and long-term agendas that emphasize the mechanisms of scientific collaboration at GWU: Developing/educating team scientists, Measuring team effectiveness, Technologies for collaboration and scholarship informetrics, Organizational supports for team science and collaboration, Faculty reward and recognition activities, Issues related to crossing cultural, ethnic, and gender boundaries in science. Seminar Objectives: assemble knowledge communities of key contributors and stakeholders from and beyond GWU to foster cross-disciplinary discourse; explore key topical areas critical to scientific collaboration; examine critical areas of academic and organizational collaboration; propose collaborative and team projects to university leadership and decision-makers; disseminate key products and create knowledge-sharing events that will enhance collaborative activity at the GW department, school, and university levels. Subscribe to the program: https://blogs.gwu.edu/collaborativeculture
37	COLUMBIA UNIVERSITY HEALTH SCIENCES	Creating an infrastructure for training research coordinators for high-risk settings	Highly skilled coordinators with regulatory and disease-specific knowledge translates into better research implementation and improved outcomes. The Clinical Research Resource (CRR) at the Irving Institute for Clinical and Translational Research, home to Columbia University's CTSA Program hub, developed a program and scalable model to support research coordinators in offsite high-risk units such as the NICU, PICU, ED and Neuro ICU. With no current consensus on the content of such research coordinator training in high-risk settings, the CRR outpatient unit manager implemented an onboarding and practicum for these coordinators. The program includes monthly hands on training sessions on lab safety, phlebotomy, specimen handling, and EKG performance. Weekly meetings are held to review GCP and identification of protocol implementation issues. In addition, monthly seminars that are open to all Columbia University coordinators highlight available CTSA and hospital resources, as well as skill-building opportunities for professional certification preparation, regulatory and financial knowledge.
38	UNIVERSITY OF CALIFORNIA LOS ANGELES	CTSA Hubs Collaborate to Design, Test and Implement Video/Electronic Informed Consent for Biobanking	Led by UCLA CTSI Associate Director Arash Naeim, this collaborative project with UC San Francisco, Irvine and San Diego produced a scalable video/electronic consent process for remnant bio-specimens and a dedicated precision medicine blood draw that has been used in ambulatory, perio-operative, and inpatient settings with over 10,000 consented. The process consists of viewing a five-minute video that explains patients' rights regarding their samples and how UCLA plans to use donated tissue, followed by completing an electronic consent form. Eighty percent of individuals who participated in the pilot for point-of-care consenting agreed to donate remnant tissue and nearly 50% agreed to also donate a tube of blood. The video, developed with input from a community advisory board, also was piloted through EPIC MyChart in over 1,000 individuals. Next steps include expanding the video from two to seven languages and developing a version for pediatrics.

ID	Hub	Headline	Paragraph
39	WEILL MEDICAL COLL OF CORNELL UNIV	CTSC Seed-Funding Awardee Michelle Bradbury and Colleagues Develop Innovative Approach to Imaging Cancer	An outstanding example of team science is the partnership that formed between Michelle Bradbury at Memorial Sloan Kettering Cancer Center (MSK) and Ulrich Wiesner at Cornell University as a result of a CTSC pilot award in 2008. the team explored Cornell (C) dots - ultrasmall silica nanoparticles with a fluorescent core - to “light up” cancer cells. By attaching radiolabels and targeting molecules to particles, C dots provide readouts of key biological properties of tumors, organs and tissues including tumor accumulation/retention, renal clearance and metastatic disease. The CTSC award allowed the team to obtain two INDs, an NIH R01 grant, and 5 patents. Dr. Bradbury received BioAccelerate NYC and Technology Development awards to commercialize the technology, and completed first-in-human studies. Elucida Oncology Inc. licensed the technology for oncology applications. The team was awarded NIH U54 grant for creation of MSK-Cornell “Center for Translation of Cancer Nanomedicines” for diagnosis and treatment.
40	ICAHN SCHOOL OF MEDICINE AT MOUNT SINAI	CureQuest Videogame: A Case Study in Team Science	Creating a sense of wonderment about the challenging process of drug discovery is no easy task. “CureQuest” rises to the challenge in videogame form. The development group, from diverse academic arenas, levels of training & disciplines (computer science, software engineer, creative writing, art, gaming, biostatistics, drug discovery, clinical trials, education, graphic design and music), gathered around a large conference table to share domains of expertise, experience and exchange ideas. Their challenge was to develop a videogame that captured the imagination of second year medical students while raising the player’s awareness, insight and appreciation for the complexities of drug discovery and development. Learning to speak the same language and investing in each other’s talents, the team created a fun-to-play game that involves a world of magic, where a mysterious condition has affected the land, and it is your job to find a treatment through the discovery and development methods of translational biopharmaceutical research.
41	UNIV OF NORTH CAROLINA CHAPEL HILL	Data Across Disciplines: NCATS' Data Translator is a Model for Team Science	NC TraCS, UNC's CTSA, in collaboration with UNC's Renaissance Computing Institute and Institute for the Environment, is participating in the NCATS-funded Data Translator initiative. The Translator aims to design a system to integrate biomedical datasets and “translate” those data into insights that can accelerate translational research. The datasets come from different disciplines (from 'omics to clinical data), essentially making Translator a giant Team Science experiment! For nearly two years, as part of the larger Translator initiative, clinical informaticians from TraCS have been working with UNC computer scientists, clinicians, and environmental scientists to solve a massive data linkage problem. In that time, we have built successful prototypes that demonstrate the utility of linking clinical data, environmental exposures data, and socioeconomic data to answer translational questions at the patient level. Our success is primarily due to our team's ability to work across disciplines and our willingness to ask questions of each other.

ID	Hub	Headline	Paragraph
42	VCU	DEAP: The Diabetes Engagement and Activation Platform	Only 25% of patients with diabetes receive structured education, which has been shown to significantly improve health outcomes. To address this need, our collaborative, cross-disciplinary team spans medicine, nursing, information technology, biostatistics, research associates and students. We've disseminated our work regionally and nationally, having recently completed a randomized trial of a novel, comprehensive online diabetes self-management program in 359 patients across 24 practices. Delivered through an electronic health record linked patient portal, the intervention uptake reached 74%, far exceeding prior initiatives, and evidencing significant findings including widespread cardiovascular risk reduction knowledge deficits. Several unforeseen challenges strengthened and solidified our team and commitment to a shared passion-empowering practices and improving the lives of patients with diabetes. We are effectively identifying and resolving barriers to patient-centered, accessible diabetes education. The strength and dedication of our team as well as study outcomes will help ensure R01 funding and translation to clinical practice.
43	UNIVERSITY OF WASHINGTON	Developing a University-Wide, Team-Based Approach for Responding to Disasters and Humanitarian Crises	The University of Washington lacks an integrated, systematic approach for faculty to respond to domestic and international disasters and humanitarian crises. This absence was the impetus for the UW's Population Health Initiative to leverage the team science and facilitation expertise of the Institute of Translational Health Sciences to convene an interdisciplinary group of faculty experts in May 2018 to begin formulating a more university-wide approach to this work. Through this convening, invited faculty members developed a thorough roster of the university's response and recovery experts to better understand the UW's diverse array of capabilities in this area. The group was also able to identify the UW's core competencies in education, research, and service in this area, discern cross-cutting areas of strength, and complete an initial brainstorming exercise to identify concrete next steps for all three areas. Work on these next steps continues and is drawing from disciplines across the university.
44	OREGON HEALTH & SCIENCE UNIVERSITY	Development of an efficient, community-based, multicenter, pragmatic clinical trial using CTSA resources	OCTRI worked in close collaboration with a pediatric dermatologist to develop a multi-center, community-based trial with many needs and challenges: •Recruit parent-infant dyads in first 6 weeks of life •Recruit from primary care practices •Geographic representation and diversity •Minimal to no demands on clinic staff, no involvement in research •Minimize impact on parents while maximizing response rate We engaged with parents and primary care practices to develop preferences for family-focused recruitment and data collection methods. We created an executive committee of primary care clinicians and network managers to develop a clinical coordinating center. The data coordinating center was developed in close collaboration with CTSA-based clinical informatics, clinical trialists, regulatory specialists and biostatisticians. The Trial Innovation Network provided support for compliance with consent policies across the 28 clinics in the four networks. This NIAMS-funded trial recruited the first patient in May 2018 and uses e-consent and data collection into REDCap.

ID	Hub	Headline	Paragraph
45	UNIVERSITY OF CINCINNATI	Diagnosing and Fixing Car Trouble – A Metaphoric Workshop on Teams	For most researchers with training in medicine, basic science, or engineering the concepts behind Team Science seem vague and "soft." We recognized that many of our learners can't relate to issues of interpersonal relationships and how high functioning teams depend on like trust and communication. To help them relate, we developed a metaphor-based workshop on diagnosing and repairing team dysfunction issues. The metaphor of the common experience of driving and maintaining a car was used to guide participants through exploration of assessing the size, scope, and seriousness of different team symptoms. Everyone was able to relate to experiencing car problems, assessing problems, and actions needed for resolution. The car problem model helped them understand the relationship between symptoms, causes, when to take action, and matching the "fix" to the problem. The metaphor model helped skeptical participants to engage more easily in learning and to develop personalized take-home messages.
46	UNIVERSITY OF SOUTHERN CALIFORNIA	Effective Collaboration in the Era of Team Science	SC CTSI organized a team science mini-symposium to address the best practices in collaborative research. Panels of experts addressed two topics: 1) developing independent academic research in an era of team science; and 2) distance collaborations. Participants included experts in team-based research with expertise securing multiple center grants, experts in the principles of team science, and administrative leadership experienced in appointments and promotions. Discussion focused on the different ways that individual researchers can demonstrate significant contributions within a team and barriers in using technology to communicate across USC campuses. Representatives from the USC IT group were among the audience who provided feedback during the networking session. This mini-symposium launched our 2019 Team Science Success Seminar Series which will focus on using technology for communication. The USC IT group will partner with us in future seminars to advise in using different technology tools for collaborations.
47	ALBERT EINSTEIN COLLEGE OF MEDICINE, INC	Einstein's Team Science Accelerator—Catalytic Seed Grants	The CTSA pilot project program leverages the CTSA's innovative infrastructure to encourage novel collaborations and build new teams. Our pilot project program features three categories of Catalytic Seed Grants (CSGs) that emphasize team science to drive programmatic objectives in clinical and translational research. One aims at Life Span Research and Studies in Special Populations to promote integration of translational science across the life span to improve health outcomes, with an emphasis on groups of the Bronx and lower Westchester population from disadvantaged racial/ethnic minorities, or those having unique environmental exposures or rare genetic diseases. We require that projects include investigators from at least two disciplines, and a defined unique patient population. For example, one recently granted CSG uses electronic sensors in asthma inhalers to study minority children and adults with severe asthma in a clinical trial of treatment adherence among patients who receive real-time monitoring/feedback vs standard therapy. The project is a team science exemplar with experts in child health, community engagement, pulmonary function, data sciences, and technology innovation.

ID	Hub	Headline	Paragraph
48	UNIVERSITY OF WASHINGTON	Engaging primary care providers to address critical health problems	The WWAMI (Washington, Wyoming, Alaska, Montana and Idaho) region Practice and Research Network (WPRN) is a collaborative group of 60 primary care practices across five states that conduct research to improve health. The WPRN developed and innovative model for engaging primary care clinicians in developing and undertaking research to address critical problems in primary care. A multi-disciplinary team, including an academic researcher and 6 primary care practice champions (3 physicians, 2 pharmacists and 1 behavioral health specialist) met monthly over the course of 1 year, by email and teleconference to develop, plan and implement a network wide study to assess the degree to which primary care practices were providing evidence-based hepatitis C screening services. Through the collaborative research, we found that there was tremendous variability in the prevalence of evidence-based screening and practices used the findings to improve care.
49	UNIVERSITY OF WASHINGTON	Engineering Innovation in Health: Interdisciplinary teams solving unmet health challenges with technical solutions	The UW Engineering Innovation in Health (EIH) program promotes interdisciplinary collaboration between engineering and the health sciences with the goal of developing technical solutions to pressing challenges in health care. Undergraduate and graduate engineering students partner with health care professionals to solve unmet health challenges identified by the clinicians. . EIH follows a need-based design philosophy that begins with an unmet health need and examines stakeholders, market opportunity, intellectual property, FDA regulations, and reimbursement. The program results in patent submissions, data for research papers and proposals, and medtech startups. Students receive degree credits and develop skills working in interdisciplinary teams. UW ITHS Team Science Core is providing tailored team science training to the student-clinician teams with the express goals of improving team dynamics, communication, and program participant satisfaction, as well as increasing the number of program deliverables, such as patent applications, startup formation rate, device clinical evaluations, and research papers.
50	UNIVERSITY OF CINCINNATI	Even Wonder Woman and Superman need a Justice League	Acute care researchers are experts at what they know and do; they use all their talents to heal children and adults in dire situations while problem solving for prevention and cures. They do so by asking experiential questions to research, build value add cases to gain funding, and employ empathetic staff to assist them through all phases of implementation including regulatory considerations for safety of human subjects. Yet why do these wonderwomen and supermen believe they MUST do it all? How can they sustain their clinical hours, mentor junior faculty, lead committees and collaborate with colleagues throughout the Academic Health Center? Their kryptonite is traditional teaching that the lone scientist paves the way to the top. In this customized Team Science Seminar, acute care principal investigators and their senior clinical research pros will learn that their diverse strengths and mindful communication leads to effective teams – their own Justice League!

ID	Hub	Headline	Paragraph
51	ICAHN SCHOOL OF MEDICINE AT MOUNT SINAI	Expedite the Virtuous Circles of Image Labeling with Imaging Research Warehouse for Artificial Intelligence-Assisted Radiological Reporting	Artificial Intelligence-assisted radiological reporting has great potential to improve healthcare. However, the scarcity of clinician-labeled radiological imaging datasets is currently the biggest bottleneck to train machine-learning algorithms. In clinical practice, radiologists are too burdened in narrative radiological reports to producing labeled region of interests (ROIs) for training image-based machine-learning algorithms; while treating clinicians who are used to viewing their patient's radiological images lack the motivation and tool to produce labeled ROIs as a radiologist would do. To meet this challenge, our CTSA is developing multiple ways to incentivize both the radiologists and treating physicians to label salient imaging features during routine radiological image reading; and our CTSA-sponsored Imaging Research Warehouse (IRW) provides a centralized imaging platform to expedite the virtuous circles of image labeling. All image ROIs produced throughout our health system will be archived in our IRW and shared to qualified investigators for future imaging research.
52	MEDICAL UNIVERSITY OF SOUTH CAROLINA	Eye Spy: A Collaboration with Gibbes Museum of Art and Medical University of South Carolina (MUSC) to Enhance Student Affective Skills	Eye Spy is a collaborative and instructional educational program created by the interprofessional faculty from the Gibbes Museum of Art and MUSC with the purpose of enhancing the observation, communication, and perspective taking skills (affective) of students studying across different fields within healthcare. Ten interprofessional students participate in different small and large group activities across eight weeks of instruction within the Gibbes. Patients and/or caregivers join students and faculty for two sessions to ensure that their voices are heard. Using video recordings, surveys, and focus groups, qualitative and quantitative data are captured to measure affective domain behaviors. Early analyses indicate improved nonverbal communication, interprofessional readiness, perspective taking, and observation skills. By improving students’ affective skills, the broader impact of creating compassionate patient and family-centered healthcare providers will be achieved. Based on this preliminary work, MUSC, Gibbes, and Medical University Hospital Authority submitted a grant proposal to the National Endowments for the Arts.
53	NEW YORK UNIVERSITY SCHOOL OF MEDICINE	Finding collaborators through data: the Data Catalog Collaboration Project	We developed the NYU Data Catalog to promote collaboration by helping researchers both make their own data discoverable and locate usable biomedical data that is not readily accessible elsewhere online. This low-barrier approach to data sharing requires only that researchers provide a description of their dataset so that researchers who are not yet ready to upload their data to a public repository can begin the process of data sharing and finding potential collaborators. NYU made the code and metadata for the catalog open source, and six other institutions -- including University of Pittsburgh, Duke, and UNC -- received support from the National Library of Medicine to implement the catalog at their own institutions. These institutions have formed the Data Catalog Collaboration Project, and are working with the National Library of Medicine and NCATS CD2H to make otherwise invisible datasets from these institutions more easily discoverable on a national level.

ID	Hub	Headline	Paragraph
54	UNIVERSITY OF CALIFORNIA, SAN FRANCISCO	Finding the Sweet Spot: Where Community Leadership and Science Overlap Against Sugary Drinks Consumption	UCSF's Community Engagement and Health Policy Program in CTSI has facilitated wins in the fight to reduce consumption of Sugar-Sweetened Beverages (SSB). Beginning in 2010, we've built relationships on trust, shared resources, evidence and community engagement. By building multi-sector partnerships with the local health department, policymakers, community-based organizations, hospitals and utilities, we've pioneered policy, environmental, systems and programmatic change. Our partnerships resulted in three SSB policies passed in 2015—including the first SSB Warning Label policy in the nation—and the "Soda Tax" of 2016. We achieved nearly 100 new water bottle-filling stations citywide, and pioneered lay health worker water promotion and SSB education in low-income communities of color, reaching thousands of people per year. UCSF staff enabled our scientists to leverage expertise in policy development, community-engaged research and recommendations for investment of over 21 million dollars in soda tax revenue to reduce chronic disease disparities in SF.
55	UNIVERSITY OF CINCINNATI	Flipcharts: They're Not Just for Breakfast Anymore	The Center for Improvement Science (CIS) at the Cincinnati CCTST has been the home of team science for at least two years now, offering a variety of training opportunities for local researchers including workshops, consultations, Grand Rounds, and a graduate-level course. Throughout all training events, our development team intentionally included a variety of interactive exercises that gave participants a chance to learn about themselves, share their past experiences working on teams, and practice new skills. In one workshop, small teams worked to build a tower out of uncooked spaghetti, tape, and a marshmallow to learn about team roles and communication. In another, participants moved into groups based on communication style and then learned how to communicate with others from a different communication style. We used apps like Poll Everywhere and Socrative to generate immediate, anonymous participant feedback. Interactivity makes learning more meaningful and applicable to participants. And more fun!
56	ICAHN SCHOOL OF MEDICINE AT MOUNT SINAI	Fostering Cross-disciplinary Research: Lessons Learned from STTEP-UP	The Sinai Team-based Translational Education Program: the URM Propeller (STTEP-UP) is a NCATS funded program through the Icahn School of Medicine at Mount Sinai. Its goal is to facilitate URM post-doctoral trainees becoming innovative leaders in clinical and translational research. Fellows participating in the program complete a team-based research project. This past year, disciplines represented by the fellows included Cardiology, Psychiatry, Neurology, and Pediatrics. Identifying a clinical question and designing an investigation was facilitated by group brainstorming meetings with program mentors. Fellows designed a project identifying unnecessary medical testing, with the goal of exploring whether provider education could reduce these ordering practices. In addition to regular in-person meetings, a free web-based sharing platform was used to foster collaboration. Fellows learned new research strategies and interpersonal skills, while also developing a sense of commonality with their peers. This experience supported the idea that cross-disciplinary research improves the collaboration and education of emerging researchers.

ID	Hub	Headline	Paragraph
57	UNIVERSITY OF WISCONSIN-MADISON	From Research to Practice through the Community-Academic Aging Research Network Story	The Community-Academic Aging Research Network (CAARN) brings together interdisciplinary teams of academic researchers and community partners to conduct clinical and dissemination research related to healthy aging. With the partnership of the Wisconsin Institute for Health Aging (WIHA), CAARN employs team science to fill a crucial evidence-to-implementation gap by helping researchers and community partners make connections, form collaborations, and conduct research to design, test, and then disseminate new community-based interventions to improve the health of adults nationwide. CAARN's community research associates, employed by WIHA, facilitate the research partnerships. To date, CAARN has assisted 21 active research projects and 28 university-community partnerships across 55 counties in Wisconsin. In 2018, CAARN will finish packaging materials for WIHA's 2019 dissemination of two new evidence-based healthy aging programs that have been proven through CAARN research teams: Mind Over Matter: Healthy Bowels, Healthy Bladder (to reduce incontinence) and Tai Chi Prime (to improve balance).
58	UNIV OF NORTH CAROLINA CHAPEL HILL	From Retreat to Research: Strategic Planning for Building a Children's Research Institute	Title: From Retreat to Research Effective Team Science praxis can lead to setting up the infrastructure for research projects, programs, or even institutes. For example, a newly developing University of North Carolina Children's Research Institute sponsored a strategic planning retreat in November 2016 to develop strategies for collaborating with leading scientists who conduct multidisciplinary research that affect children, with the long-term goal of successful pediatric team science endeavors in NC. Attendees were assigned to one of six different pre-identified research areas of strength (ie, themes). Questions governed the conversations to elucidate research and education strengths, and needs for the Institute, which led to a number of significant short-term gains including: an interactive seminar series to promote interdisciplinary collaboration among highly successful pediatric research teams and researchers across the university; a new advisory board; a patient registry/biorepository working group; and an intramural grant program – all in a span of about nine months.
59	SCRIPPS RESEARCH INSTITUTE	From Sequencing to Disease: Intern Collaboration Kickstarts Platform for Faster Diagnosis	Scripps' objective to improve and expedite patient diagnosis by combining metagenomic sequencing with data science was significantly accelerated with the aid of three interns this summer. While working on a Zika related study, researchers in the Andersen lab started a "wouldn't it be cool" list for their analysis of metagenomic sequencing data. When the interns arrived, the list was waiting, and they were quickly put to work developing independent - but interrelated - computational tools, to facilitate data and disease analysis. Ashwath created a database showing pathogen, disease and symptom relationships. James developed a machine learning tool to annotate pathogens, diseases, and symptoms in publications. And Garyk developed a platform to visualize and interpret metagenomic data utilizing the database and tools created by Ashwath and James. Mentoring the 2018 summer interns has proven to be a highly valuable investment by the Andersen lab, sure to pay significant dividends well into the future.

ID	Hub	Headline	Paragraph
60	PENNSYLVANIA STATE UNIV HERSHEY MED CTR	From slushies to smoothies: How Summer Camp Revamped has transformed Boy Scouts summer camps	Boy Scouts of America is one of the largest youth-serving organizations in the US, operating 420 scout camps. Penn State PRO Wellness partnered with 7 camps across 3 states to implement Summer Camp Revamped using the health belief model, in which camps chose from a menu of evidence-based improvements. This included healthy messaging, rearrangement of food displays, menu modifications, and price-point advantages. The goal of this work was to understand implementation fidelity and acceptance to inform policy decisions. Camps made improvements to food offerings in the dining hall and camp store while maintaining costs and satisfaction scores. On average, camps implemented 71% of the menu recommendations provided. Nutrient analysis shows reduction of added sugars, saturated fat, and carbohydrates. Findings from this study resulted in evidence-informed policy incorporating specific language and supporting best practices to further develop more robust nutrition standards for camps nationally, with potential to impact 2 million scouts.
61	PENNSYLVANIA STATE UNIV HERSHEY MED CTR	From slushies to smoothies: Improving nutrition at our nation's Boy Scout camps	Boy Scouts of America is one of the largest youth-serving organizations in the US, operating 420 scout camps. Penn State PRO Wellness partnered with 7 camps across 3 states to implement Summer Camp Revamped, using the health belief model, in which camps choose from a menu of evidence-based improvements. This included healthy messaging, rearrangement of food displays, menu modifications, and price-point advantages. Camps made improvements to food offerings in the dining hall (n=7) and trading post (n=5) while maintaining food costs. On average, camps implemented 71% of the menu recommendations provided. Nutrient analysis of menus (n=3) shows reduction of added sugars (-38%), saturated fat (-24%), and carbohydrates (-19%). Food sales increased in two camps with an average of 33% healthy product inventory in all participating camps (n=5). Feedback scores (0 to 10) from troop leader evaluations were positive regarding menu variety (7.0), portions (7.8), and TP products (8.1).
62	EMORY UNIVERSITY	Georgia CTSA Academic Partners Team Up to support MRSA Studies at Children's and Grady	In MRSA (methicillin resistant Staphylococcus aureus) research,–what is the best evidence-based treatment for patients (especially children). Georgia CTSA-supported research investigator Lilly Immergluck, MD, conducted three clinical trials to better understand the antibiotic resistant bacterium, community-associated methicillin resistant Staphylococcus aureus used multiple Georgia CTSA resources: Clinical Research Sites; funding; collaborative investigators; and collaborative services. The study also benefited from Georgia CTSA's-secured partner reciprocity. Immergluck: "As a pediatrician and pediatric infectious disease specialist, I've seen many children and families impacted by this pathogen over 15 years. MRSA originated from the community, not the hospital. However, its presence has become so prevalent; it is now also a hospital-acquired source of infection. I see families in despair whose child is critically ill from severe infections, those who struggle with recurrent MRSA infections. Their stories which continue to fuel my passion to make some impact on decreasing the circulation of this antibiotic resistant staph."

ID	Hub	Headline	Paragraph
63	EMORY UNIVERSITY	Georgia CTSA investigators in biostatistics team up as `Trio in Biostatistics'	Emory biostatisticians Amita Manatunga, PhD, Limin Peng, PhD, and Ying Guo, PhD, Georgia CTSA - BERD program investigators and experts known for their collaborative research, have been working together for more than a decade. They publish eight to ten papers a year sharing and rotating the role of lead author. Their collaboration led them to try and develop “new statistical methods that could allow them to identify neuroimaging or biologic biomarkers corresponding with different subtypes of disease classes as well as with other traditional clinical scales of mental disorders.” BERD program provides value-added assistance to markedly improve the quality of the translational and clinical research. Highly trained, service oriented BERD personnel from Emory’s Rollins School of Public Health, MSM’s Clinical Research Center & Research Center for Clinical & Translational Research, Georgia Tech’s Stewart School of Industrial and Systems Engineering, and UGA’s College of Public Health are available to assist early career researchers.
64	UNIVERSITY OF PITTSBURGH AT PITTSBURGH	GETTING THE WORD OUT – MAKING RESEARCH MATTER	Community organizations and researchers unite to think of creative ways to disseminate research through WORDOUT grants. Annually, grantees are chosen to work directly with researchers and use the funds – ranging from $2500 to $10,000 - to think outside of the box to craft best practices for research literacy and participation. Ultimately, this was reported to have diversified outreach, and produced equitable data. This year University of Pittsburgh researchers will be matched to community organizations with a non-profit status. The community leaders are tasked with serving as the experts in what issues needs to be addressed, best practices for identifying solutions, and find avenues to disseminate research. The matchmaking opportunity lends to community organizations adopting a researcher and then teaching them about diversity and inclusion, and how to hold meaningful discussions about research with their constituents. The researcher gains a pipeline to the community they seek to embed their study.
65	MAYO CLINIC ROCHESTER	Got IDEA?: Immunology, Data-Science, Epidemiology and Aging – Research Team Tackles Challenges of Childhood Asthma	In an ongoing NIH R01 and Mayo supported study, Mayo Clinic Immunologist (Hirohito Kita), Data Scientist (Hongfang Liu, Co-PI), Epidemiologist (Young Juhn, PI) and Researcher of Aging (Robert Pignolo) tackled two major challenges of childhood asthma: 1) while only a subgroup of asthmatics develop serious asthma-associated infectious and inflammatory comorbidities (AIICs), they are a serious health threat and it is unknown how to identify such children at a population level (clinical). 2) It is also unknown why AIICs occur in some asthmatics and addressing this question may broaden the understanding of asthma from being merely an airway disease (scientific). The Mayo IDEA team addresses these two challenges through: 1) Natural Language Processing-powered computational phenotyping which identifies such subgroup of asthmatics replicated by unsupervised analysis and immunological characterization and 2) immunophenotyping using immunosenescence markers. The renewal R01 study aims to further develop and refine computational phenotyping, and identify biomarkers for AIICs.

ID	Hub	Headline	Paragraph
66	ICAHN SCHOOL OF MEDICINE AT MOUNT SINAI	Hacking our way to Team Science Success	It's 5pm, Friday October 13th 2017. An event space in New York City hosts 87 individuals who have never previously met. Each has a different professional background (including healthcare, biomedical science, computer science, engineering and business), experience and expertise. The one thing they have in common is a desire to innovate and make a difference in the lives of others. They have come together to create the next innovative technology-based solution that could change healthcare forever. The energy in the room is electric. Welcome to the Mount Sinai Health Hackathon! Over the next 60 hours these individuals immersed themselves in multidisciplinary teamwork, learning to communicate across the boundaries of their professional disciplines and fields of inquiry. Pitching to a panel of judges, the winning teams venture to turn their ideas into reality. The final result? Fourteen newly formed transdisciplinary teams who emerged to create innovations beyond our imagination.
67	MAYO CLINIC ROCHESTER	Health research conversations at the garden	In collaboration with community partners, the Mayo Clinic Community Engagement Program developed Garden Cafés, to engage the community with researchers through bi-directional informal dialogues about health research topics. Since September 2015, Garden Cafés have been co-hosted by Mayo staff and community partners at the local community garden. Selection of discussion topics are guided by the Olmsted County community health needs assessment, which identified community health priorities. For example, to address mental health, the Garden Café connected an adolescent depression researcher with the community, and resulted in development of a joint research project. Garden Cafes serve as a viable option to educate community stakeholders on health issues and their roles in the clinical research, which builds understanding and trust, and a pathway to develop community-academic science teams. The impact on community members is seen through making connections with their neighbors and health researchers to make sustainable health improvement. http://www.kimt.com/content/video/490524081.html
68	Other (You'll be asked to specify hub institution below)	Health Sciences Entrepreneurship Boot Camp Stirs Students' Commercial and Translational Aspirations	The Health Sciences Entrepreneurship Boot Camp, a residential training program, has three primary goals: 1) broaden career development activities to prepare students for the biomedical workforce; 2) support educational activities to increase awareness of entrepreneurial activity and the potential to commercialize ideas; and 3) reinforce the practice of team science as a mechanism for health science innovation. The Camp guides students through an immersive, five-day training program in which they learn how to start a company, work with faculty, entrepreneurs and mentors, and become exposed to business processes, regulatory requirements, patents and legal issues. Students form teams, create and refine new venture ideas and interview potential customers. Eligible students are undergraduate, graduate, and medical students. The Camp concludes with a Demo Day during which the teams present their new venture ideas. Since its inception in 2016, the annual Camp has received high scores and has catalyzed four biomedical startup companies.

ID	Hub	Headline	Paragraph
69	WEILL MEDICAL COLL OF CORNELL UNIV	Heart-to-Heart Community Outreach to Underserved NYC Communities – Educating Medical Students and Uplifting NYC	Weill Cornell CTSC's Heart-to-Heart Program (H2H) is a unique collaborative team of volunteer faculty, staff and students (MD, MD/PhD, RD, Nursing, PA and pre-med) from Weill Cornell Medicine, Hunter School of Nursing, Hunter College and NewYork-Presbyterian Hospital. The team travels to underserved communities throughout NYC with the goal of identifying new cases of diabetes for early intervention, heart and eye disease, and intervening early in the disease process. Health screenings include point-of-care blood tests, medical/nutrition consultations, and referrals to follow up care and insurance enrollment. Students learn to work together as a multi-disciplinary/multi-institutional team early in their careers and learn to assess factors that affect healthcare or make lifestyle changes. Since 2009, H2H held 105 events, screened over 5,500 individuals, identified over 1,870 new cases of diabetes/prediabetes, high blood pressure and high cholesterol. H2H continues to educate students and uplift community members through community-based research and team science.
70	UNIVERSITY OF CINCINNATI	High-Functioning Teams Receive Love and a Plaque	The UC College of Medicine presents annual awards for outstanding research. This is a decades-old tradition and, over the years, has recognized research innovations, rising stars, multi-institutional collaborations, and highly impactful projects. The College of Medicine Research Cabinet regularly reviews the types of awards to recognize important changes in the research environment. In 2015, the Cabinet recognized the importance of cross-disciplinary collaboration to translational research. They created the Team Science Award to highlight teams of diverse make-up that had developed new areas of collaborative research and had been successful in using principles of team science to create high-functioning teams. The inaugural award was made to the Cincinnati Interprofessional Care Collaborative (CICC). This group had over 30 members and a dozen different disciplines from all partners of our CTSA and the community. They had a grant success rate of over 85% and had been awarded over $6 millions in external funding.
71	UNIVERSITY OF CINCINNATI	I'm Not a Team Scientist but I Play One on TV	COMING SOON! FREE and OPEN to the PUBLIC! Clinical Translational Research or CTROnline - www.med.uc.edu/ctronline - a library of the BEST online modules and recordings by the Center of Clinical and Translational Science and Training (CCTST) program cores: Translational and Workforce Development and Center of Improvement Sciences, Team Science Training will be offered without a password protected account. (Say what? WOW I can never remember my passwords anymore but I love to learn!) Examples of online classroom Team Science modules are: Assembling Effective Teams, Conflict Management, Five Dysfunctions of a Team, Leading Effective Teams, Personality Colors/Communications Exercise, Team Trust-Building, Team Charters, and Team Effectiveness. All CCTST Grand Rounds topics will be archived here for access. No more excuses for continuous learning; now the classroom has come to you!

ID	Hub	Headline	Paragraph
72	VCU	iCubed: Transdisciplinary Research Improves Health Outcomes and Daily Lives of Richmond Citizens	The iCubed Health and Wellness in Aging Populations Core represents an innovative approach to advancing team science. A key component of this Core is the Richmond Health and Wellness Program, a clinical and teaching collaborative model that is a health promotion and wellness program centered on older adults residing in low-income housing. This program has served as a strong foundation for the transdisciplinary research team that has been formed in the Core to address the needs of low income community dwelling individuals across the lifespan in the Richmond area. Bringing researchers together from different disciplines, including Nursing, Pharmacy, Medicine, Epidemiology, Gerontology, Urban Planning, and Anthropology, provides opportunities to transform and improve the health of the community and daily lives of individuals while advancing science. The unique skills and knowledge of the research team include expertise in clinical care of older adults, pharmacogenomics, alcohol and substance abuse, urban issues, cultural issues, and population-based epidemiology. The Core members have adopted a team science approach to foster the transdisciplinary team culture and team building.
73	CASE WESTERN RESERVE UNIVERSITY	Ideas Moving Parents & Adolescents to Change Together (IMPACT)	Combining clinical, behavioral, and community expertise, IMPACT, a three-year intervention study involving overweight and obese middle-school students from Cleveland, Ohio and their guardians, compared the effects of two family-based behavioral interventions on BMI change over 36-months. The study also assessed the additive value of an enriched school environment through participation in We Run This City, a community-supported, school-based fitness program designed to increase physical activity offered in Cleveland schools (another example of community, school, and academic teams collaborating on interventions). IMPACT was designed with several features to improve its effectiveness over past studies of vulnerable population obesity management, including strong community engagement and testing a novel approach to behavior change (SystemCHANGE). While this study resulted in null effects on BMI, it sparked new research questions on how social determinants and environmental characteristics can influence health and the ability to take advantage of intervention strategies.
74	UNIVERSITY OF ROCHESTER	Implementing Advice from Community Engagement Studios	How: Adding to the Vanderbilt model of Community Engagement Studios which allows investigators to get input from community members at any point during their research - in framing a research question, designing a protocol, recruiting participants and disseminating results. What & When: We collate notes from the 2 hour studio discussion and summarize by giving the researchers 2-3 key recommendations from the studio. After 6 months we follow-up with researchers to see which recommendations they were able to implement and what challenges they faced. Why: Getting researchers and community experts to talk with each other is important and the most crucial part is making sure that those discussions have an impact. By summarizing the discussions with key recommendations and following-up with researchers we're able to track the real impact of studios and address challenges that researchers face in implementing community suggestions.

ID	Hub	Headline	Paragraph
75	STATE UNIVERSITY OF NEW YORK AT BUFFALO	Innovation Labs: Testing a Creative Approach to Forming Transdisciplinary Research Teams	Developing transdisciplinary teams for translational research projects is challenging at any career stage, but especially early in one's career. The University at Buffalo CTSI partnered with the Vanderbilt CTSA to conduct and assess two Innovation Labs to facilitate team formation. Grounded in deliberate creativity methodology, an Innovation Lab is a facilitated 5-day residential event to create research ideas and proposals to address a grand challenge. We randomized groups of ~50 early-stage investigators from wide-ranging disciplines into two groups—one Innovation Lab group and one control group. We hosted two Innovation Labs on pressing national health issues (opioid misuse and obesity interventions) where multiple teams were formed, proposals were designed, specific aims were written and follow-up plans were created. The enthusiasm of participants was extraordinary. We are following the two Innovation Lab groups and corresponding control groups to assess their efficacy in facilitating formation of new teams, collaborations and proposals.
76	UNIVERSITY OF WASHINGTON	Innovations in Practice Transformation: Linking Health Professions Education and Team Science	We highlight an Academic-Practice partnership between the University of Washington (UW) School of Nursing and UW Medicine to develop, implement, and evaluate a team-based, patient-centered model of care for advanced heart failure (AHF) care teams. The partnership formed in 2014 to meet the following goals: 1) collaboratively identify a focal work process that could improve team functioning, and 2) test whether facilitated implementations of work process changes and purposeful team training led to improvements in team-based care. The focal work process chosen was shifting from conference room or hallway rounds to structured interprofessional bedside rounds. Steps taken to improve team-based care and support the work process change included - forming a change management team, facilitating leadership development workshops, conducting team trainings, and utilizing evaluation data to support continuous process improvement. Baseline measures and three annual follow-up surveys demonstrated improvements in team functioning, mutual respect, and satisfaction among staff and patients.
77	UNIVERSITY OF WASHINGTON	Innovative Model to Connect Regional Healthcare Systems to National Multisite Clinical Studies	Translation of scientific discoveries into practice requires collaboration between researchers, clinicians, and healthcare systems where most patients receive care. The ITHS and our clinical partners in Washington, Wyoming, Alaska, Montana and Idaho have developed a model to enable regional healthcare systems to join multisite clinical studies emanating from the national CTSA Trial Innovation Network (TIN). This model, which leverages existing research infrastructure, includes criteria to assess innovation, relevance, and feasibility within regional healthcare settings. Tools have been developed to enable prospective sites to submit comments about and indicate interest in TIN opportunities. A pilot project connected three regional healthcare systems to a study that will evaluate a new diagnostic approach to improve risk stratification in patients with coronary ischemia. Development of the model has extended the reach of the TIN beyond academic centers and can enable access to the TIN by clinicians in nonacademic settings for their own multisite studies.

ID	Hub	Headline	Paragraph
78	UNIVERSITY OF WASHINGTON	Integrating Hands-On Team Science into the University of Washington's ITHS TL1 Program	The goal of the ITHS TL1 program is to create a cross-disciplinary community of emerging researchers and provide them with specific training and career development opportunities. To help trainees develop the skills needed to function effectively within translational science teams, we have partnered with our new ITHS Team Science Core to integrate focused team science training into the program. Guided by the CTSA's Core Competencies in "Translational Teamwork" our year-long program now features readings and interactive content on: 1) identifying your team, 2) professional roles and stereotypes, 3) personal and conflict communication styles, 4) forming multidisciplinary collaborations. Trainees also participate in a translational science "field trip" to the research setting of a fellow trainee from a different disciplinary background (e.g. engineering student visited a pharmacy laboratory). New in our 2018-2019 cohort, teams of trainees are collaborating on a translational research proposal (NIH R21 format) that they pitch to key stakeholders.
79	COLUMBIA UNIVERSITY HEALTH SCIENCES	Integrating Special Populations (ISP) at the Columbia University CTSA Program Hub	At the Irving Institute for Clincial and Translational Research, home to Columbia University's CTSA Program hub, we created a new ISP Resource in 2016 to encourage and support collaborative cutting edge clinical research for diseases across the lifespan in four domains: HIV, geriatric, pediatric, and rare diseases. Our specific goals include addressing changes in the clinical environment reflecting the increasing pediatric presence of formerly adult diseases (e.g., type 2 diabetes) and pediatric-to-adulthood transitions of patients with formerly fatal pediatric diseases (e.g., cystic fibrosis). The ISP facilitates relevant collaboration development, demonstration of broader research considerations, and dissemination of information to communities. Our ISP team consists of 12 members representing community engagement and investigators across the four domains. Annually, we sponsor 6-10 seminars and 3-4 pilot awards for multidisciplinary studies of disease across the lifespan. Each pilot provides $40,000 and must include a young investigator and at least two of the ISP domains.
80	TUFTS UNIVERSITY BOSTON	Investigators Host Community Orientation to Clinical Research	Tufts Clinical and Translational Science Institute believes research is most impactful when it addresses issues important to patients, communities, clinicians, and other stakeholders. To provide targeted opportunities for stakeholders to participate in research, we created a Stakeholder Expert Panel. We recently hosted a Research Orientation for Panel members that included an introduction to the research process, tours of a lab and the Clinical Translational Research Center, and a presentation on comparative effectiveness research. Community members then discussed what they learned, and how they envisioned their involvement in the research process. Participants felt they could recognize the steps involved in conducting translational research, and generated recommendations for future research-related topics. Since then, Panel members have worked on designing a training module to teach non-investigator stakeholders how to review grant proposals, and designing a learning module (with the Integrating Special Populations team) on recruiting older adults to research.

ID	Hub	Headline	Paragraph
81	NEW YORK UNIVERSITY SCHOOL OF MEDICINE	Investing in Research Staff: Strategic Teamwork for Effective Practice - Mentor Development Program (STEP-MDP)	Skillful research staff members are critical to productive translational research teams and yet their ongoing professional development is rarely formally addressed. Through the Strategic Teamwork for Effective Practice-Mentor Development Program (STEP-MDP), we aimed to both create a community of practice for research staff and build the skills needed to enhance research team performance. We selected 32 participants from among the NYU Schools of Medicine, Social Work and Nursing for the first two STEP-MDP cohorts. We delivered three, 2-hour workshops focused on team communication, identifying team areas for improvement, and mentorship/coaching skills. Sessions featured brief didactics, group learning and exercises based on participants' real issues. A variety of active learning techniques such as brainstorming, role-playing, problem solving, and peer coaching were used. On a retrospective-pre/post self-assessment, participants reported significant improvements in each of five domains as a result of the program: Communication, Leadership, Empowerment and Motivation, Coaching, and Community.
82	INDIANA UNIV-PURDUE UNIV AT INDIANAPOLIS	Jam with us! Research needs your expertise.	Research Jam is the Indiana CTSI's Patient Engagement Core. We are a team of human centered designers, visual designers, physician researchers, and research coordinators who work with doctors, patients, families, and community members to ensure that health research, interventions, and communications are as relevant as possible to the community they aim to serve. Research Jam uses a human centered design research approach to engage stakeholders with different backgrounds, and therefore, areas of expertise to explore, create, and test solutions to difficult problems around health, wellness, and research. We have assisted over twenty researchers in addressing issues such as recruitment, retention, defining patient centered outcomes, dissemination, program development, and study acceptability. We have worked in health areas including infant mortality, opioid misuse, diabetes, adolescent behavior, pediatric obesity, surgical decision making process, and cancer. Research Jam has been a part of 14 successful grant applications totaling over one million dollars in funding.
83	UNIVERSITY OF WISCONSIN-MADISON	Laying the groundwork for successful Learning Health Systems	Last fall, Marshfield Clinic Research Institute (MCRI) began implementation of a virtual Research Project Management Office (RPMO) to help connect the growing number of MCRI project managers to each other. Through participation in RPMO bi-monthly workgroup meetings, we've established a platform for PMs from different scientific disciplines within the Institute to exchange ideas and share best practices. The RPMO is establishing centralized resources for PMs to expand their toolkit with new knowledge that can be applied to current projects and imparted to their project team. Additionally, they are finding personal enrichment through career development opportunities, such as PMI conferences and guest speakers, the RPMO promotes. As part of MCRI's Hub and Spoke structure, the RPMO model promotes greater collaboration between MCRI research support groups and its research centers. RPMO also aligns well with the health system's Project Management Office thereby laying the groundwork for successful future Learning Health System endeavors.

ID	Hub	Headline	Paragraph
84	UNIVERSITY OF CINCINNATI	Leaders and Managers Speak Different Languages of Love	Participants in a Team Science workshop completed a pre-work survey where they were asked to think back on a leader they have greatly admired and use a five-point Likert scale to rate them on 20 qualities. During the workshop, participants learned that half of the pre-work questions were about leadership and half were about management and that the two domains had different functions. Leadership was defined as setting direction and motivating the team while management was defined as developing plans for carrying out the work and monitoring the results. Participants learned that leadership and management skills can be either found in one person or split across two people on a team, and that highly functional teams must have both leadership and management. Group discussion explored the challenges teams face when one of these functions is missing and how to make up the deficit.
85	UNIVERSITY OF WASHINGTON	Leading By Example – University of Washington's ITHS Team Science Training BootCamp	The ITHS Team Science Core Team provided training in Teamwork, Communication, Agreements (goal alignment, roles, responsibilities, team processes including lean project management), Social styles, Bias, and Leadership through experiential training. Core ITHS faculty and leadership teams attended a mandatory 1.5 day Team Science Bootcamp in grant year 1. The teams focused on their grant aims as they worked through application of the team competencies. Responses to the training included: "I didn't know anything about Team Science before this boot camp, & the knowledge gained was very helpful." "It was a good use of time. I think it helped the overall ITHS team and our team got some new ideas". 70% of the participants found every activity relevant or very relevant. 69% of the respondents indicated that the Bootcamp positively influenced feelings of psychological safety. Since the Bootcamp, there have been 4 separate requests for similar training for additional grant teams.
86	CHILDREN'S RESEARCH INSTITUTE	Let's Build an App: Encouraging Team Science through a Medical App Hackathon	The CMTS Module at Children's National in Washington DC has sponsored an annual hackathon for each of the past 2 years where teams come together to present ideas for medical apps for mobile phones. For each hackathon we solicit ideas in advance and select the best 10 ideas for presentation at the event, which was attended by 50 people representing many different disciplines. Teams of 4-6 people are then formed to work on the idea at the event and a Powerpoint template is given for each team to complete. The top three presenters as selected by a group of judges were then given a seed fund to develop a prototype app as well as assistance in moving their app forward. From this year's event, which was held in March, there were two finalists that have already developed prototypes apps and are making plans to deploy the app in clinical trials.
87	UNIVERSITY OF CINCINNATI	Low Trust Teams Have No Problem with Conflict	In 2018, the Center for Improvement Science (CIS) within the Cincinnati CCTST will conduct a workshop on Conflict Management and Teams. Low trust teams have no problem with conflict because they ignore it and never resolve it. Conflict is difficult especially on teams. There may be conflict with leaders, between team members, or conflict regarding the tasks you are doing for the team. We will examine conflict and resolution using the Thomas-Kilman categories of competing, collaborating, compromising, avoiding and accommodating. Then we will discuss the Circle of Conflict Adaptation and the categories of conflict around Relationships, Data, Language, Interests, Structure and Values. There will be table discussions as well as role-playing to practice methods of conflict resolution. We will discuss different methods to use based on your position on the team. Participants will discuss methods they have used to deal with conflict and what worked or did not work.

ID	Hub	Headline	Paragraph
88	UNIVERSITY OF KANSAS MEDICAL CENTER	Jargon Police Force: Making Meetings Safe!	Jargon, acronyms and insider-baseball talk is comfortable for some and alienating for others. To reduce training, scientific and linguistic barriers and to encourage everyone feeling welcomed, respected and that they can contribute meaningfully to the discussion, our meetings start with deputizing everyone as a member of the Jargon Police Force. This means that at any time, anyone can (politely) interrupt or intercede quickly to ask for a definition or for what the letters of an acronym mean. Meeting organizers model the behavior (sometimes by interrupting each other!) as jargon inevitably pops up. Not only can this be fun, it encourages participants to be mindful of how they can each contribute to a culture of mutual respect and sharing.
89	UNIVERSITY OF KENTUCKY	KL2 Emulsion: “Hearing and Nurturing” – Growing Team Science from within the CCTS Training Platform at the University of Kentucky	Drs. Tina Studts and Matt Bush met as KL2 scholars within the UK Center for Clinical and Translational Science. Through CCTS career development programming, the scholars in pediatric public health and otolaryngology, respectively, merged their interests to explore behavioral and community-based interventions to improve adjustment and reduce disparities among rural families affected by pediatric hearing loss. This pair engaged other scientists and community partners with overlapping interests and additional skills to create a productive program of collaborative science. From securing initial support from the CCTS Junior Investigators Pilot Grant Program, the pair have gone onto secure a K award (Bush), an R34 (Studts), an implementation science pilot grant (Studts/Bush), an R01 (Studts), and have another R01 (Bush) that has been resubmitted following favorable initial review. Combining the science of hearing and the science of parenting has provided a compelling CCTS team science success story built within the CCTS training platform.
90	UNIVERSITY OF CALIFORNIA LOS ANGELES	Potent drug combinations against e. coli number in the thousands, CTSI researchers find.	Scientists have traditionally believed that combining more than two drugs to fight harmful bacteria would yield diminishing returns. The prevailing theory is that that the incremental benefits of combining three or more drugs would be too small to matter, or that the interactions among the drugs would cause their benefits to cancel one another out. Now, a research team led by KL2 scholar Pamela Yeh has discovered thousands of four- and five-drug combinations of antibiotics that are more effective at killing harmful bacteria than commonly believed. The team's findings, reported in the journal npj Systems Biology and Applications, could be a major step toward protecting public health at a time when pathogens and common infections are increasingly becoming resistant to antibiotics. Yeh is an assistant professor in the College of Letters and Science; her boundary-crossing team includes scientists at the David Geffen School of Medicine.
91	UNIV OF NORTH CAROLINA CHAPEL HILL	Problem-driven collaborations between clinicians and engineers leads to new solutions to unmet clinical needs	In an exciting initiative to create translational research opportunities while seeding new collaborations between clinicians and engineers, members of the Joint Department of Biomedical Engineering at UNC joined clinical faculty in the Department of Neurosurgery to identify and prioritize a broad range of unmet clinical needs, a subset of which were then targeted for further development. This initiative began with presentations of research strengths in engineering with group discussions of immediate clinical problems, resulting in a preliminary set of over thirty neurosurgery needs being identified. Needs spanned such areas as self-diagnosing implants, new simulation and clinician training technologies, to novel ultrasound applications. Iterative and more detailed discussion surrounding these needs have since led to multiple grant submissions along with student engineering team design projects leading to invention disclosures. This problem-oriented team approach shows significant promise in helping to accelerate solutions to real-world problems while fostering team science.

ID	Hub	Headline	Paragraph
92	University of Iowa	Team Science Training Provided to 111 Multidisciplinary Researchers	Based on letters of intent from 81 potential teams applying for pilot research funding, we chose 25 teams to participate in team science training and submit full proposals. Each member of the 25 teams (111 scientists from 58 different university departments and outside institutions) participated in a 2-hour training session. Content focused on benefits and pitfalls of working in teams. Training techniques included short lectures, real-world case scenarios, small group activities, and large group discussions. Key areas of emphasis included the formation of effective teams and the importance of alternating between planning/reflection and task work. Post-session ratings using a 1 (not improved) to 5 (greatly improved) scale revealed ratings of 4 or 5 from 76% of participants concerning improved knowledge about team science processes and 80% concerning improved skills for building an effective team. Five teams chosen to receive funding participated in team facilitation over the ensuing 18 months.
93	UNIVERSITY OF CINCINNATI	Team Science: A 12 Step Program	To provide team science training to a broad audience of investigators, we developed a series of workshops and seminars covering basic and advanced topics including practical tools and resources to support high-functioning cross-disciplinary teams. After 2 years and over a dozen educational activities, we saw an increase in evaluation comments asking for more "science of team science" content. At the time of our 2015 CTSA grant refunding application, we proposed a graduate credit course on the topic: Collaboration and Team Science. We incorporated it into our KL2 program and added as an elective to our Certificate and Master's programs in CTR. At the Committee on Graduate Education review meeting, program directors from around the university indicated that they were interested in incorporating this course into their own programs. After the successful completion of the inaugural offering, several programs have inquired if they could require it in their program curricula.
94	UNIVERSITY OF MICHIGAN AT ANN ARBOR	MCubed Brings Teams Together	MCubed is an innovative team science program, which provides real-time seed funds to new multi-unit faculty trios to explore high-risk approaches to pressing interdisciplinary research challenges that might not immediately attract conventional funding. Formed "cubes" have only two requirements 1) faculty members must come from two or more UM units and 2) 50% of funds must support the next generation of researchers, including undergraduate students, graduate students, or post-doctoral fellows. Since its inception in 2012, MCubed has catalyzed over 450 cubes, involving over 3000 faculty, students, and staff in cutting-edge projects across every UM school and college in Ann Arbor and Dearborn. MCubed teams have secured more than $110M in follow-on funding, 200+ publications, and 75% of cubes include new research collaborations. Now MCubed is expanding its team science efforts to include other Michigan universities including UM Flint, Northern Michigan University, Wayne State, and Michigan State University.

ID	Hub	Headline	Paragraph
95	UNIVERSITY OF MICHIGAN AT ANN ARBOR	Mind Your Ps and Us: Promoting Team-Based Research Through Large-Scale Grant Submissions	The pursuit of large-scale grant funding, including NIH P- and U-series mechanisms, provides an opportunity for faculty to translate their individual research successes into cross-disciplinary team efforts poised to tackle complex research problems. To encourage faculty to leverage collaborative relationships, create integrated research visions and submit competitive large-scale grants, we created a novel plan of support for faculty across our university. The elements of our support plan include: 1) strategic input on conceptualizing the grant and building the team, 2) $100,000 pilot awards, 3) administrative support, 4) proposal management, 5) grant editing, 6) educational resources and 7) team science leadership training. Although still in its infancy, we have assisted with 19 large-scale grants and provided $300,000 in funding to position teams for grant development; we will soon host a team science training for 40 select faculty. We are partnering with campus units to expand our services, funding and target audiences.
96	PENNSYLVANIA STATE UNIV HERSHEY MED CTR	Monday's market: A cross-functional collaboration to address healthy food access	Lebanon County, Pennsylvania, experiences economic hardships and elevated obesity rates. To address obesity and obesity-related behaviors such as diet and exercise as identified by a community health needs assessment, Penn State PRO Wellness established a cross-functional collaborative, Better Together in 2016. We used the social ecological model to identify key stakeholders to participate and brought together over 80 like-minded organizations to discuss needs and priorities in Lebanon County. Action teams led by community co-chairs were established in March 2017 to address healthier food access, physically active communities, and engaging youth and families. Monday's market was established by Better Together, a collaboration among many local non-profits to support an outdoor farmer's market, container gardening, food demonstrations, financial literacy education, meal planning, fitness classes, and more. This market reaches Lebanon residents in downtown Lebanon at the YMCA.
97	ICAHN SCHOOL OF MEDICINE AT MOUNT SINAI	Multidisciplinary Teamwork Modelling for Leadership Training: a Team Science Competency	The LEAD (Leadership Emerging in Academic Departments) Program, (a partnership between ConduITS, UTSW CTSI, Icahn School of Medicine at Mount Sinai's Office of Academic Enrichment & Development (OADE), and Mount Sinai Health System's Talent Development & Learning Department), incorporates curricular content from diverse fields of knowledge to promote active learning and teaming. The program faculty communicates, engages and collaborates with one another across professional (medicine, education, instructional technology, psychology, organizational behavior, law and business) boundaries, to develop educational content and to model collaborative multidisciplinary teamwork for clinical and basic science junior faculty participants from diverse disciplines. Throughout the 12-month LEAD program, faculty role-model interprofessional behavior and competencies, and demonstrate what can be achieved through effective collaboration. This fosters a culture for LEAD program participants themselves to develop a junior faculty Community of Practice with a new regard for teaming, and eventually emerge as leaders in their own right.

ID	Hub	Headline	Paragraph
98	MEDICAL UNIVERSITY OF SOUTH CAROLINA	New Interactive On-Line Gaming Activity Helps Build Teamwork Skills Among Graduate-Level Healthcare and Science Students	SCTR and MUSC have recently developed an interactive team-building game designed to challenge teams with tasks that demand effective communication, leadership, collaboration, and cooperation. This game (“Sloppy Mountain Medical Center”) is similar to “Escape Room” challenges growing in popularity across the nation. In this online experience, teams work together to discharge patients from a disorganized and confusing hospital as quickly as possible. Most of the information and resources needed to discharge patients from each player’s room is contained in other team members’ rooms. This forces teams to work together, communicate clearly, collaborate effectively, and plan efficiently. 800 students (250 teams) participated in this interactive activity in January of 2018 at MUSC. After participation, 95% of students indicated that they were “able to use effective interprofessional team communication approaches and strategies” and 96% indicated that they were “able to identify communication strategies that work well when collaborating as a team.”
99	MEDICAL UNIVERSITY OF SOUTH CAROLINA	Novel Bench-to-Bedside Collaborative Teamwork in Developing a Stroke Rehabilitation Game	This story shows the direct impact of South Carolina Clinical and Translational Institute (SCTR) has in promoting cross-disciplinary translational research team collaborations across the T1-T4 translational spectrum. SCTR held a scientific retreat on ‘stroke rehabilitation, recovery and regeneration’ related research where an occupational therapist at the Medical University of South Carolina met a computer engineer at Clemson University and began a collaboration that resulted in a new stroke rehabilitation game, Duck Duck Punch, a low cost computer game; intellectual property; won 2nd place worldwide in the Microsoft Imagine Cup International Kinect Challenge (500 applicants); received funding from the Delaware Clinical and Translational Research Institutions (ACCEL) to transition the technology out of the lab to clinical practice; formation of a company; commercialization of the technology and a Phase II Small Business Innovation Research to further test whether it provides superior benefit to an off-the-shelf video game.
100	OREGON HEALTH & SCIENCE UNIVERSITY	OCTRI’s Biomedical Innovation Program fosters innovation and improves human health through a commercializati on-focused grant program	OCTRI developed the Biomedical Innovation Program (BIP) to spur innovation by providing early stage funding ($40K), mentoring, project management, and access to entrepreneurial education for faculty inventors in the health sciences. The BIP has two application cycles each year – one for software, device, and diagnostics and another for drug discovery. OCTRI works closely with OHSU’s technology transfer office to vet proposals and protect IP. The overarching goal of the BIP is to accelerate the delivery of healthcare technologies from academia to the marketplace and, thereby, to improve human health. Vital to the program’s success has been the ability to leverage the knowledge and experience of Oregon and Southwest Washington’s bioscience community. The program has attracted participation, and in some instances, financial support from state-funded agencies and industry alike. Since 2013, twenty-eight awarded projects have resulted in over $12M in follow-on funding, 6 start-up companies, and 21 active patent applications.

ID	Hub	Headline	Paragraph
101	OREGON HEALTH & SCIENCE UNIVERSITY	OCTRI'S CTRC & OHSU faculty partner with State of Alaska to uncover rare gene variant	In 2003, the State of Alaska Newborn Screening Program started using a more sensitive mass spectroscopy method, which revealed a spike in the frequency of a rare variant in the CPT1A gene among native communities in north Alaska. CPT1A codes for an enzyme essential in fatty acid metabolism. OHSU's team discovered that the variant has a 70% penetrance in the Alaskan native population, and may be associated with increased child mortality and infections. In 2014, five homozygous children and their families underwent multi-day inpatient testing in the OHSU CTRC during medically supervised fasts to determine the physiologic mechanisms of this variant. The children had blunted ketone production during fasting, and two became hypoglycemic. OHSU subsequently worked with the State of Alaska to institute universal DNA-based screening of newborns and an education program for affected families to help these children avoid hypoglycemia when ill.
102	EMORY UNIVERSITY	Out of the Blue (Sky Group): Collaborative Discussions Bringing Big Results	Blue Sky Groups are convened to bring together diverse researchers to discuss common themes and identify potential areas of collaboration. These groups meet once to discuss a broad topic, and then future meetings or online forums are organized based on interests and potential outcomes. Health Service Research Day, the Health Services Research Course and the Georgia Center for Diabetes Translation Research all came out of a Blue Sky Group. The first CTSA Blue Sky Group on Software Solutions to Clinical Problems was held in June 2018 and hosted thirty clinicians and researchers from three of four Georgia CTSA institutions. We had a lively discussion around the capabilities of FHIR and current unsolved clinical problems. Conversations have continued over a listserv and group members are now serving as mentors for the Georgia Tech health informatics course, where students solve real-life clinical problems. We're excited to see what results from future sessions!
103	SCRIPPS RESEARCH INSTITUTE	Peeling back the curtain for successful team science	Erica OllmannSaphire (@eosaphire) of Scripps Research shares successful team approach to Ebola research with the twitterverse: https://twitter.com/EOSaphire/status/1027639828429656065 She shares research findings in an open forum while highlighting the value of team science. Remarkably, this work brought together "43 (many previously competing) labs across 5 continents, academia, government and industry." Highlights: "Thanks to the institutes who shared samples, analysis, time & resources @scrippsresearch @ragoninstitute @PHAC_GC @USAMRIID @utmbhealth @EmoryUniversity @VUMC_Vaccines @astar_research @Inserm @AECOM @Uwvetmed @LosAlamosNatLab @UniofOxford @UnivOfTexas @IcahnMountSinai @Regeneron" "Fun interactive tools from the Ebola mAb VIC study from @gkay92 and @K_G_Andersen are here: http://apps.vhfimmunotherapy.org/ Interactive data explorer and correlation network to explore links among mAb features and to in vivo protection @CellCellPress @cellhostmicrobe #VIC" "More to come from this unique collaboration to understand antiviral antibodies as therapeutics and from vaccines: engineering, other viruses, different features, novel mAbs, different animal models. Join us! #VIC" Check out the full thread!

ID	Hub	Headline	Paragraph
104	UNIVERSITY OF PITTSBURGH AT PITTSBURGH	Pitt Innovation Challenge (PInCh): Challenging creative minds to tackle difficult health issues	The Clinical and Translational Science Institute at the University of Pittsburgh created PInCh in order to stimulate the translation of novel problem-focused research into the community by giving researchers a venue to be creative, develop new ideas, and work with people beyond their usual sphere of collaborators. PInCh is an annual multi-round competition that starts with submitting a video describing the idea and culminates in a pitch to judges in front of a live audience. Teams consist of at least one Pitt faculty member but can otherwise be made up of individuals from other institutions, community groups, or businesses. Each winning team receives an award for directs costs and project management support to execute a 12 month project to take the solution one step further along the path of development. Since 2014, PInCh has engaged 1153 innovators resulting in 43 translational projects with over $5M in post-award investment.
105	NEW YORK UNIVERSITY SCHOOL OF MEDICINE	Planting the seeds: research networking for KL2/TL1 scholars and alumni	The NYU-H+H Clinical and Translational Science Institute (CTSI) has hosted a networking event for the past two years to foster collaboration across the New York City CTSA hubs, bringing together KL2/TL1 trainees and alumni from NYU, Icahn School of Medicine at Mt Sinai, Rockefeller University, Albert Einstein School of Medicine, Weill Cornell University and Columbia University. Each institution was asked to suggest current trainees to give 15-minute presentations of their research to kick off the event, with three selected each year. This was followed by an hour of “speed research networking” in which the participants were paired up and given five minutes to discuss their research before moving on to the next pairing. The evenings concluded with further opportunities to make connections over wine and cheese. Over the two years, more than 60 trainees and alumni participated in this event and follow-up surveys indicated that over 40% met potential collaborators and over 90% were interested in future networking events.
106	UNIVERSITY OF WASHINGTON	Promoting “Scientific Success” Across Clinical Research Settings	Successful conduct of high-quality research involves evaluation of scientific merit, clinical relevance, feasibility, and resources. Contextual factors within clinical research settings may impact study execution, leading to variations in work practices and challenges to measuring success across research settings. Members of the Northwest Participant and Clinical Interaction (NW PCI) Network, a collaborative group of 12 clinical and translational research centers, affiliated with hospitals, healthcare systems, clinics, and universities across Washington, Wyoming, Alaska, Montana and Idaho, are building “scientific success” review into their study start-up processes. Three NW PCI sites collaborated in an assessment of the types of review performed at each organization and identified key differences in processes (e.g., centralized versus decentralized), as well as gaps (e.g. feasibility assessment, biostatistical review). Together, these sites have established best practices, based on CTSA Consensus Working Group recommendations, and developed standardized processes supported by a workflow template for adoption across NW PCI sites.

ID	Hub	Headline	Paragraph
107	WEILL MEDICAL COLL OF CORNELL UNIV	Promoting Team Science Through Domain-Specific Research Data Repositories	Research teams require access to domain-specific data for retrospective studies. The CTSC hypothesized that no single data repository could meet the needs of diverse research teams. We proposed the concept of customized research data repositories (RDRs), leveraging i2b2, REDCap, and custom data marts to support investigator groups sharing similar research interests. From 2014-2018, the CTSC deployed RDRs in anesthesiology, digestive health, epilepsy, myeloproliferative neoplasms, neurological surgery, ophthalmology, pulmonary and critical care, and stroke. Each RDR integrates data from disparate clinical and research applications, including electronic health records and next-generation sequence systems, and supports cohort discovery using i2b2, collection of novel measures with REDCap, and analysis of row-and-column data sets using data marts. To date, RDRs have supported studies of chemotherapy response, diabetic macular edema, colon cancer surveillance, and cirrhosis, among others. RDRs encourage collaboration and the formation of new research teams by providing rich data marts in well-defined research areas.
108	STATE UNIVERSITY OF NEW YORK AT BUFFALO	Promotion and tenure for Team Science Faculty: A Case Report	I chair a department of Biomedical Informatics with 34 faculty. One faculty member who I recently took through promotion and tenure was a team science faculty. Here is what i learned: A) There are certain characteristics of team science faculty 1. They do not have large grants as a PI. However they have many more grants where they are a Co-I. 2. They have fewer first and last author publications. However they have more high impact middle author publications. 3. They may or may not have teaching experience. B) Things I learned that helped me lead to this promotion and tenure: 1. For every grant - Have the proponent define their contribution to the project. Including anything that they developed (code, ontology, models etc.) and who went on to use that material in future science. 2. For every middle author publication - Define the role on the project and again as above anything developed and who has used those artifacts. 3. Have the letters speak to the value of what was produced by the faculty member in the work on their grants and manuscripts.
109	UNIVERSITY OF CALIFORNIA-IRVINE	Reconstructing communication: Unlocking integrative capacity and creative potential in interdisciplinary medical research teams	Little is known of the types of individual-level cognitive processes that lay a foundation for team-level integration. Each member's reaction—automatic or intentional (see Kahneman, 2013)—to new information offered by teammates shapes the extent to which individuals modify their own task-relevant interpretive schemas to coordinate more effectively—a process that is an essential precursor to team knowledge integration. We refer to this as "perspective integration capability" (PIC)—an individual-level precondition to team knowledge processing that enables expertise exchange and integration. In a field experiment of 30 multidisciplinary biomedical faculty research teams from CTSAs around the nation, we compared the outcomes of teams in the experimental condition, exposed to an intervention to enhance PIC and team performance, compared to a control condition. Preliminary results suggest that teams who received the communication training intervention engaged significantly more in perspective integration over time, which in turn facilitated improved team creative performance.

ID	Hub	Headline	Paragraph
110	UNIVERSITY OF ROCHESTER	Research Methods Forum: An interdisciplinary team approach to help investigators develop successful research proposals	The Research Methods Forum at University of Rochester provides an interactive setting for investigators to present new and developing research ideas to multidisciplinary experts in clinical research methods and potential collaborators, who will provide recommendations. The core team consists of experts in biostatistics, epidemiology, clinical trials, and recruitment and retention. This team is supplemented on an ad hoc basis with experts in areas of relevance to a particular research idea being discussed. Written feedback is provided to presenters after the forum, summarizing the key points discussed. Since the inception of the forum in the spring of 2016, 49 presentations have been hosted. Twenty-four grant proposals have been submitted; 12 proposals have been funded and 3 received a competitive impact score. Presenters represented 28 different academic departments. Forum participants were drawn from 27 different departments within the University of Rochester, and several presentations were attended by experts from outside the university.
111	WAKE FOREST UNIVERSITY HEALTH SCIENCES	Research Teams Set Charters for Success!	The Wake Forest CTSI's Team Science Program hosted a 3-hour team development workshop for research teams facilitated by team science experts from our associated business school. Research teams registered for the seminar which required at least half of the team to attend. Within 48 hours of announcement, the workshop was fully subscribed and a waiting list developed (a repeat performance is scheduled for Fall). Teams sat together at tables and worked on trust building, team science best practices and building their team charters. They left with homework and enthusiasm to engage in the process. Surveys reported 82% were satisfied or very satisfied with the workshop. The most common comments from participants were that they wanted more time and additional workshops like this one (A follow-up with the same teams is planned for the Fall). Six of 7 teams report that their teams are working together better since the workshop.
112	UNIVERSITY OF CINCINNATI	Researchers Learn Their Colors for Better Communication	Our Basic Team Science workshop includes a communication style assessment taken from the Whole Brain Business Book written by Ned Herrmann. This 10-question assessment allowed researchers to better understand their preference for communicating in terms of four major domains: The bottom-line, down in the details, interpersonal relationships, or the big picture. Participants learn strategies for communicating with colleagues from all four domains. Teams typically have eureka! moments after taking the assessment and reflecting on problems they've experienced. Some researchers were enlightened about communication styles different from their own and they better understood why some team members seemed to be in constant conflict-and how to fix it! Teams also came to recognize the value of having diverse communication styles among their members. This tool was freely shared with workshop participants and is becoming widely used across the Academic Health Center to improve team function.

ID	Hub	Headline	Paragraph
113	ROCKEFELLER UNIVERSITY	RU String Quartet Leadership Program Inaugurates Team Science Leadership Initiative at Rockefeller University CTSA	To inaugurate the new team leadership initiative for KL2 Clinical Scholars (https://www.rockefeller.edu/education-and-training/clinical-scholars/team-science-initiative/), we invited Mr. Benjamin Wolff and his string quartet colleagues to describe the dynamic and fluid nature of team leadership in the quartet (https://www.bwolff.com). The program was highlighted by a musical performance during which the Scholars observed the seamless transfer of leadership throughout the work as different instruments were featured. The musicians then discussed the importance of trust and appropriate recognition of each member's contributions. The program stimulated a broader discussion by the Scholars, members of the quartet, and the members of the Senior Staff of the Rockefeller University CTSA comparing leadership of a clinical research protocol team with leadership in a string quartet. Feedback from the Scholars after the program emphasized how focusing on teamwork and team leadership in another field stimulated them to think about the unique features of translational research team leadership.
114	MEDICAL UNIVERSITY OF SOUTH CAROLINA	Scientific Retreats to Stimulate Cross-disciplinary Translational Research Team Collaborations	To stimulate the formation of new cross-disciplinary translational research team collaborations and innovative pilot projects, the Medical University of South Carolina's CTSA, South Carolina Clinical & Translational Research Institute (SCTR), has initiated scientific retreats often with “speed dating” style networking sessions. The themes of the retreats are cross-disciplinary, address unmet medical needs in South Carolina, and are anticipated to generate pilot project and extramural grant applications related to novel translational research discoveries. The retreat format is designed to stimulate new research ideas and exchange in small groups to develop new research projects. The retreats are complemented by the SCTR Pilot Project Program funds to support innovative pilot projects that emanate from the retreats. We have sponsored 18 retreats to date, with 1400 attendees from the statewide universities, hospital and state agencies, and community groups. Outcomes include 65 new teams formed to successfully address stroke, obesity, and tobacco related health issues and resulted extramural grants resulting $42:1 return on investment.
115	SCRIPPS RESEARCH INSTITUTE	Searching for Answers through Molecular Autopsy	Medical Examiners must often tell grieving families that the cause of a previously healthy loved one's death is unknown even after an extensive autopsy. This news can be frightening given that they may themselves be at risk for a catastrophic event. While many genetic causes of sudden unexpected death (SUD) are known, government agencies rarely have the resources for expansive genomic analysis. Scripps Research has built a diverse team of physicians, geneticists, bioinformaticists, and statisticians, combined with regional medical examiners to apply genomic sequencing to cases of SUD. The study provides Scripps researchers a unique and meaningful opportunity to share research findings with relatives of the deceased and has evolved based on feedback from participating families. The program now accepts cases from across the country as we seek to identify novel genetic explanations for SUD, evaluate the implementation of a molecular autopsy program, and link participating families with medical resources.

ID	Hub	Headline	Paragraph
116	UNIVERSITY OF SOUTHERN CALIFORNIA	Shaping Teams of the Future: Incentivizing Team Science	SC CTSI offers a $5,000 team building voucher to provide rapid funding for activities that promote the goal of assembling new multidisciplinary teams focused on clinical and community research, particularly those grounded in science of team science principles. Examples include supporting conferences, workshops and/or seminars, new research partnerships across disciplines, and exploring virtual platforms. Currently, 10 teams have been funded and have launched their activities. SC CTSI administrators participate as observers or facilitators to understand the needs and the barriers to research team formation across diverse activities. Surveys and in-person interviews with core team leaders are conducted after the funding period to further probe success and barriers to research team formation. Two areas identified as barriers include: 1) organizing/leading effective meetings; and 2) remaining engaged between meetings. These topics will be addressed in future Team Science Success seminars to foster good practices in team science.
117	DUKE UNIVERSITY	Show Us the Money: A Team Science Approach to Supporting Faculty in Search of Funding	Our team developed a Funding Opportunities application (FO) that recommends both internal and external funding opportunities to users based on their research profile and interaction with the displayed content. Currently being piloted by faculty volunteers, FO was inspired, designed, and developed with a 'Team Science' mentality. Through series of needs assessments and focus groups, faculty prioritized the need for support finding funding in a fractured and overwhelming landscape. The initial concept and design underwent five rounds of iterations with guidance from faculty in 23 different disciplines. We were advised by data scientists, librarians, and research development staff for the machine learning, database, and curation components respectively. Additionally, FO connects with existing institutional systems to improve collaboration and reduce user burden. Finally, our pilot users come from every department in the School of Medicine and are given the opportunity to provide critical feedback to ensure that FO is flexible and functional.
118	MEDICAL UNIVERSITY OF SOUTH CAROLINA	Smartphone App Improves Communication and Teamwork in Trauma Care	Coordination and communication in trauma care is improved through shared access to patient and contextual information for preparation and planning prior to patient arrival and during the first phase of care. Observational and interview studies of trauma teamwork identified the need for an app to (i) provide patient and injury details prior to arrival in the ED (ii) allow secure text and picture messages (iii) integrate with clinical workflow, reduces disruptive and unreliable phone calls from the trauma team, to anesthesia, imaging, operating rooms and intensive care personnel. The app was used at MUSC in 367 traumas during a 3 month trial period. In comparison to baseline (no app), teamwork scores significantly improved, flow disruptions were significantly reduced, and patients spent significantly less time in the Emergency Department. User-centered design of communication technology leads to improvements in teamwork, process, efficiency, and may yield better patient outcomes.

ID	Hub	Headline	Paragraph
119	COLUMBIA UNIVERSITY HEALTH SCIENCES	Stimulating New Team Science Collaborations and University-Wide Partnerships	The Irving Institute for Clinical and Translational Research, home to Columbia University's CTSA Program hub, brings together various and disparate groups across our institution, partner organizations, other CTSA Program hubs, local and regional governmental entities, and key community stakeholders around significant research areas and initiatives. In collaboration with the Data Science Institute, a workshop held in 2017 focused on bringing together experts from four domains (phenotypic characterization, environment, data science, and genomics) to advance complex traits research through data science-enabled precision medicine/health approaches. More recently, using a similar approach to address the opioid crisis, an initial environmental scan of institutional activities culminated in development of four working groups, a brainstorm session, a steering committee, and a larger symposium to stimulate collaborations among researchers, industry partners, and the community. Both efforts led to synergizing new partnerships, developing key areas for further research, and creating targeted pilot funding opportunities to facilitate this research.
120	University of Miami Miller School of Medicine	Stop, drop and roll: Firefighters and Scientists Team Up to Put Out Cancer Risks	In 2015, firefighters, government officials, and scientists teamed up to launch the Firefighter Cancer Initiative (FCI) to address the excess burden of cancer among Florida firefighters. A multidisciplinary team of biomedical, communications, and education and human development investigators collaborate with key stakeholders to study firefighters' exposure to carcinogens, their risks for developing cancer, and methods of education for prevention, screening, and early detection. Projects span all translational phases, including studying and tracking environmental exposures, developing mouse models, collecting and analyzing bio-specimens, surveying active and retired firefighters, developing a cancer registry, providing cancer screening, and developing educational tools. Since launching, over 3,000 firefighters have participated in FCI. Investigators have published seven papers addressing cancer in the fire service, risk reduction education is being incorporated in firefighter training curricula, fire departments are adopting risk reduction practices, and over 4,000 post-fire on-scene decontamination kits have been distributed to 405 fire departments across Florida.
121	ROCKEFELLER UNIVERSITY	String Quartet Leadership Program Inaugurates Team Science Leadership Initiative at Rockefeller University CTSA	To inaugurate the new team leadership initiative for KL2 Clinical Scholars, we invited Mr. Benjamin Wolff and his string quartet colleagues to describe the dynamic and fluid nature of team leadership in the quartet (https://www.bwolff.com). The program was highlighted by a musical performance during which the Scholars observed the seamless transfer of leadership throughout the work as different instruments were featured. The musicians then discussed the importance of trust and appropriate recognition of each member's contributions. The program stimulated a broader discussion quartet by the Scholars, members of the quartet, and the members of the Senior Staff of the Rockefeller University CTSA comparing leadership of a clinical research protocol team with leadership in a string quartet. Feedback from the Scholars after the program emphasized how focusing on teamwork and team leadership in another field stimulated them to think about the unique features of translational research team leadership.

ID	Hub	Headline	Paragraph
122	UNIV OF NORTH CAROLINA CHAPEL HILL	Successful Research Collaborations Follow “Engineering Solutions to Health Problems: A Workshop	On April 17, 2015, the NC TraCS Team Science Resource successfully connected engineers and clinicians in a workshop titled “Engineering Solutions to Health Problems” to build research relationships and durable, feasible projects for the future. During the workshop, groups of clinicians and engineers worked together on one of four broad categories – Mobile Health, Clinical devices, Rehabilitation, and a Wild-Card category. The workshop attendees produced “need statements” that were then discussed in working groups throughout multiple sessions and molded into feasible research projects. Because of the enthusiasm displayed from workshop attendees, a shorter workshop called “Engineering a New Wave of Nursing Science” was held at the UNC School of Nursing. This one-hour workshop expanded on “need statements” and created well-articulated project ideas. Out of the 20 project ideas created during this workshop, 10 are being actively pursued as NC TraCS Team Science works to connect workshop attendees with engineer collaborators.
123	UNIVERSITY OF CINCINNATI	Surprise! Chit Chat Not a Waste of Time	In Spring 2018, the Center for Improvement Science (CIS) within the Cincinnati CCTST offered a workshop on the 5 Dysfunctions of a Team. We spent a lot of time in the workshop talking about the importance of building team trust, which serves as a bedrock for high functioning teams. A lot of researchers believe a high functioning team meets task goals and demonstrates productivity by meeting deadlines and performance metrics like grants or publications. Our training team contended that getting to know team mates on a more personal level and taking time to enjoy each other builds trust and, in effect, can help prevent other dysfunctions like fear of conflict and lack of commitment. Maintaining positive attitudes, team curiosity, zest, and playfulness are all ways to improve team dynamics. After all, a team that plays well together, works well together!
124	UNIVERSITY OF CINCINNATI	Surprise! Team Members Have No Idea What Their Job Duties Are	In a variety of workshops, team consults, and a graduate course, we have introduced team charters to attendees. A team charter is a living document that team members write together and sign. Ideally, a charter should be developed early in the cycle of team formation as it sets team direction, defines team member roles, and establishes rules of engagement for team members. Team charters commonly include sections such as Team Purpose, Team Duration, Scope of Work, Timeline, Members, Team Goals/End Result, Team Resources, Reporting or Communication Plan, Team Interactions and Behavioral Norms, and Deliverables. Few participants have previously heard of team charters, but they are regularly mentioned as one of the most useful tools participants leave with after a training session. Don’t underestimate the power of a shared mental model! By working together to spell out expectations, team members can potentially avoid a lot of heartache and dysfunction.
125	UNIV OF NORTH CAROLINA CHAPEL HILL	Symposium Gathers Teams of Researchers to Address Opioid Addiction and Overdose	On Thursday, May 25, 2017, NC TraCS held a five-hour symposium called “Combating Opioid Addiction and Overdose: Advancing Science and Policy,” on the UNC campus. The event gathered over 100 interdisciplinary researchers to discuss different ways to address the epidemic. After an introduction by funders, attendees divided into two tracks. In one track, the audience learned more about the history and current approaches to the opioid crisis. In a separate track, they worked in teams to develop research ideas in the following areas: the importance of big data; community-driven approaches in urban and rural populations; pre-clinical research; clinical research; and the intersection between the opioid epidemic and HIV/hepatitis C. At the end of the meeting, representatives from each group presented a summary to the entire gathering of the strengths, weaknesses, opportunities, threats, and needs they identified in their specific theme area and potential projects they would like to pursue.

ID	Hub	Headline	Paragraph
126	UNIVERSITY OF CALIFORNIA, SAN FRANCISCO	Tackling Cancer in San Francisco with a Community, Academic and Health Institution Team Partnership	Cancer is the leading cause of death in San Francisco and a major population health burden. To tackle this challenge a determined inter-disciplinary team that includes the SF Dept. of Public Health (SFDPH), University of California San Francisco as the organizing partner, Kaiser Permanente, other health systems, the American Cancer Society, community organizations and advocacy groups launched the San Francisco Cancer Initiative (SF CAN) in 2016. SF CAN is a long-term, public-private team partnership aimed at reducing cancer and its debilitating consequences by utilizing an integrated, transdisciplinary approach to implement evidence-based interventions tailored for diverse populations across the city. The initial focus is on the five most prevalent cancers with proven prevention interventions: breast, prostate, colorectal, liver and lung and other tobacco-linked cancers. Uniquely, SF CAN takes an integrated team approach across multiple systems and multiple cancers to translate science into applications to improve population health.
127	DUKE UNIVERSITY	Teaching Biostatistics to Medical Investigators through In-Person Workshops and Online Training Materials	A key aspect to facilitating team science involves educating junior investigators regarding the need for methodologists on their teams. Our Education Team addresses this by creating in-person workshops to introduce investigators to basic statistical methods stressing the benefits of team science and collaborating with methodologists early in the research process. In these workshops, methodologists give one-hour introductions through relevant case-studies, followed by one-on-one sessions with attendees to discuss their specific research problems, initiating new collaborations with our Biostatistics, Epidemiology, and Research Design Core. So far, 18 workshops have been given to 10 departments and more are continually being requested. Some of the case-study sessions have been turned into online materials that researchers can conveniently access at any time from our website. Additionally, we organize at least twelve applied biostatistics seminars annually that are accessible to all researchers across Duke, and provide a repository of all educational materials developed by methodologists.
128	UNIVERSITY OF WISCONSIN-MADISON	Team Approach to Funding Decisions (Community and Academic Reviewers)	Our CTSA T2-4 Pilot Awards Program (PGP) provides incentives for community-partnered, collaborative & interdisciplinary research. Reviews are conducted by a team of academic and community reviewers. After rigorous scientific review by academic experts a select group of meritorious applications are then reviewed by the External Community Review Committee (ECRC). The ECRC leverages the expertise of the community to address the challenge of translating research to real-world settings and communities. The ECRC re-ranks applications using community engagement criteria. Per this publication, the ECRC changed funding decisions 21% of the time, such that projects were “rescued” for funding because of the ECRC review. Rescued projects provided a substantial return on investment (grants and pubs). This innovative, team approach to review blends the perspectives and expertise of both academic and community reviewers resulting in the best selections for funding and achieving truly transformational outcomes.

ID	Hub	Headline	Paragraph
129	UNIVERSITY OF CINCINNATI	Team Astonished by How Dysfunctional They Are	–In 2016-2018, the Center for Improvement Science (CIS) within the Cincinnati CCTST hosted “Team Effectiveness”. Workshop attendees completed the Team Assessment Inventory responding based on a current team’s dynamics. They received a copy of their assessment responses with answers correlated with The Five Dysfunctions of a Team by Patrick Lencioni. We reviewed each dysfunction followed by a table discussion of the positive of each: building trust, mastering conflict, achieving commitment, embracing accountability and focusing on results. As a large group, we then discussed one possible intervention, setting a common goal for your team. The team should discuss and agree on several objectives needed to achieve the goal, create a timeline for expected deliverables and who is responsible. Finally set up a dashboard to track your progress and update the team regularly or develop a team scorecard and rewards for behaviors that achieve results.
130	UNIVERSITY OF CINCINNATI	Team Collaboration: A View from the Marshmallow Tower	What can 1 yard of tape, 1 yard of string, 20 pieces of uncooked spaghetti, and one marshmallow teach you on a beautiful fall day at the University of Cincinnati? That no matter how much education you have, Kindergarteners are smarter than team science gurus when it comes to jockeying for power. This is an extraordinary lesson on collaboration, innovation and creativity. The goal of this challenge is to construct a tower out of the elements that you have in front of you. The key is it has to stand up! Here is the problem, we want to manage and delegate and find a solution to execute. The kids spend no time on trying to become the boss, start with the marshmallow, and build potential towers from there. We incorporate this, and other, team-building exercises in all our team science workshops. The focus is on active learning with practical lessons.
131	UNIVERSITY OF CINCINNATI	Team Dysfunction Pyramid: Mummy Told Me Not to Come	The University of Cincinnati CCTST Center for Improvement Science (CIS) regularly conducts a popular workshop on the 5 Dysfunctions of a Team. Patrick Lencioni’s Team Dysfunction Pyramid is presented as one model for understanding how teams address challenges to becoming “high functioning.” We use Lencioni’s team functioning assessment tool to help learners understand how their teams are addressing these challenges. Using active learning strategies, participants climb each level of the pyramid, with opportunities for small group learning activities and contextualization of learning by asking participants to reference previous experiences they might have as members of dysfunctional teams. Learners come to recognize that team dysfunction is a lived experience shared by all! The combination of insight and practical tools for addressing team dysfunction has resulted in teams developing specific plans for improving their level of function. We plan long-term follow-up of teams to assess changes in their assessment scores.

ID	Hub	Headline	Paragraph
132	UNIVERSITY OF WISCONSIN-MADISON	Team Science and Human Factors Engineering Research	By building on the work system model of Smith and Carayon (1989) the CTSA-supported Systems Engineering Initiative for Patient Safety (Carayon etal, 2006) provides a framework in which team science is readily applied by promoting various disciplines to jointly analyze and improve aspects of the system. For example, in a recent AHRQ-funded grant addressing the sociotechnical design of systems for prevention and management of venous thromboembolism (VTE), transdisciplinary participation by physicians, nurses, pharmacists, statisticians, human factors engineers and cognitive psychologists produced multiple tangible outcomes. These included identification of sociotechnical design requirements for VTE prophylaxis systems and a clinical decision support application within an existing electronic health record designed to facilitate the appropriate clinical pathway for patients presenting with symptoms of pulmonary embolism. User input through interviews and focus groups, surveys, observation and data analysis combined with research expertise offers significant promise for successful implementation of both research- and team-driven outputs.
133	MEDICAL UNIVERSITY OF SOUTH CAROLINA	Team Science Approach to Study the Pain and Fatigue Self-management	The Symptom Self-Management Center for Pain and Fatigue in the College of Nursing at the Medical University of South Carolina was established in 2015 with P20 funds awarded to a nurse scientist and a biobehavioral scientist to establish infrastructure, mentorship, collaborations and resources to support pilot projects that build research teams led by nurse scientists studying pain and fatigue self-management. It relies on three cores of the South Carolina Translational Research Institute: the Biomedical Informatics Center, the Technology Application Center for Healthful Lifestyles, and the Centers for Community Health Partnerships. Four nursing faculty lead self-management teams: exploring an app-based approach to HIV-associated fatigue; developing a pain-tracking app for children with sickle cell disease; devising a breathing device and training app to strengthen COPD-affected respiratory muscles; and creating a home-based telehealth yogic breathing program for age-related pain and fatigue.
134	UNIVERSITY OF TEXAS MED BR GALVESTON	Team Science Competency Model Established to Provide Evidence-Based Foundational Skills to the CTSA Consortium	At the 2017 Science of Team Science Conference, a group of nationally prominent team scientists led by UTMB researchers presented an evidence-based competency model for team science training to address a universally acknowledged need. The model was developed to address known issues in team science and is applicable to nascent and advanced teams. Three competency sets and nine specific competencies have been proposed consisting of Awareness and Exchange (Sharing Unique Information/Promotive Voice, Inquiry/Probing, Reflecting and Integrating), Psychological Safety (Perspective Seeking, Acknowledging and Inclusion, Addressing Issues and Resolving Conflict), and Adaptation and Correction (Monitoring/Debriefing, Reflecting/Analyzing, Creating Change/Development Planning), with twenty-seven specific behavioral markers. Using NCAT's funding at UTMB, this competency model is now in development as a web-based training program called TeamMAPPS (Team Methods to Advance Processes and Performance in Science), which will be field tested by six CTSAs and made available to the CTSA consortium by the fall of 2019.

ID	Hub	Headline	Paragraph
135	UNIVERSITY OF CINCINNATI	Team Science Denier Adopts Team Charter	In Fall 2016, the Center for Improvement Science (CIS) within the Cincinnati CCTST hosted its first series of three Team Science workshops: Creating & Assembling Teams, Team Effectiveness, and Effective Team Leadership. Attendees were primarily teams of researchers who had received internal grant funding. These teams were mandated to attend the workshops as a condition of their research funding, leaving many attendees feeling disgruntled about being forced to sit through three two-hour workshops about topics they felt they already understood. However, the CIS training team was able to win many attendees over by presenting evidence-based, practical tools for improving transdisciplinary team effectiveness. One team even adopted a Team Charter and shared with the larger workshop audience how they did it and what made it useful! Lesson learned: don't REQUIRE faculty to attend team skills training or if you do, use their expertise to demonstrate practical application of team science principles.
136	YALE UNIVERSITY	Team Science Education: Interpersonal Relationships and Leadership in the Workplace	This two-year course is part of the Yale National Clinician Scholars Program, an interprofessional fellowship program, partly supported by the CTSA TL1 training grant, which prepares physician and nurse clinician leaders to improve health and health care through scholarship and action at the national, state, and local levels. Daryn David, PhD, a clinical psychologist and leadership coach, teaches the course. Upon completion, fellows understand the complexities of personal presentation, interpersonal and organizational dynamics, and workplace leadership; gain actionable techniques for communicating effectively and working harmoniously on teams; and develop their capacity to exercise leadership skills and qualities. Instruction is both didactic and experiential. Class time is used for lectures, group discussion of important scholarly readings, and for reflective discussions where fellows discuss hands-on application of skills and techniques and explore challenges and successes in becoming leaders in their fields. The course consistently earns the highest ranking within the program curriculum.
137	VANDERBILT UNIVERSITY MEDICAL CENTER	Team Science Enables a True Learning Healthcare System	A Learning Healthcare System (LHS) can bridge between knowledge generation and healthcare delivery, promoting research-informed practice, and practice-informed research. To succeed requires synergy across clinical and research enterprises, which we achieve with a VICTR-enabled, team science-based platform. The LHS Team consists of a transdisciplinary and cross functional group of faculty, staff, and advisors with expertise in clinical operations, trials, pragmatic research, implementation science, health communication, medical education, community, informatics, ethics, regulatory, and biostatistics. Collaboration with healthcare administration completes the cycle, closing the evidence-practice gap. While there are dozens of trials in the portfolio, team impact is demonstrated by two recent studies published together in the NEJM that both showed, compared with saline, balanced crystalloids resulted in a lower rate of death from any cause. Our medical educators are now studying which teaching platforms change fluid utilization so we do what we learn, as well as learning from what we do.

ID	Hub	Headline	Paragraph
138	UNIVERSITY OF CALIFORNIA LOS ANGELES	Team Science for Integrative Graduate Training in Health Delivery Science	The Cedars-Sinai Master's Degree in Health Delivery Science (MHDS) is an accredited program designed to train tomorrow's healthcare leaders. Students are embedded within teams operating in Cedars-Sinai. To be prepared for a successful career in emerging health care fields and to be equipped to improve health care delivery requires students gain multidisciplinary expertise and enhance their ability to work in teams. We tackle this challenge by creating a training ecosystem for our students comprised of leaders, clinicians, and scholars at both Cedar-Sinai and UCLA; all bound together by expertise in the emerging Science of Team Science. Students in the Master's program receive a 6-week intensive course in team science covering topics ranging from building to leading an effective team. Students are also required to integrate team science best practices into their healthcare delivery capstone projects. This comprehensive traineeship model is innovative, evidence-based, and aligned with changing workforce and healthcare needs.
139	UNIVERSITY OF WASHINGTON	Team Science makes a difference in rural areas facing the opioid epidemic	A collaboration between the ITHS, the Kaiser Permanente Washington Health Research Institute, and six rural-serving primary care clinic organizations across Washington and Idaho successfully addressed long-term use of opioids for chronic pain with significant reductions in use of high-dose opioids and the total number of patients using opioids for their chronic pain.
140	YALE UNIVERSITY	Team Science Makes Dream Work – It Paves the Way for Scientific Success	"The whole is greater than the sum of its parts" is what team science is about. As part of the computational biology and bioinformatics (CBB) Ph.D. program at Yale, we offer a graduate course (CBB750): "Core Topics in Biomedical Informatics and Data Science", which covers a range of foundational data science topics including database, ontology, natural language processing, machine learning and high-performance computing. The students are required to work together in team projects. In previous semesters, we had each team partner with an CTSA-affiliated informatics postdoctoral fellow who provided a set of data-driven research questions. The students divided their labor and applied what they had learned throughout the course to integrate and analyze the datasets related to these research questions. Finally, each team gave a written report, presentation, and shared the code through GitHub. Not only did the students complete the projects successfully, but they also enjoyed the team spirit!
141	MEDICAL UNIVERSITY OF SOUTH CAROLINA	Team Science to Integrate Primary Care and Behavioral Health for Trauma-Impacted Youth	An interdisciplinary/interprofessional (IP/ID) team was formed leveraging the expertise of biobehavioral health, primary care specialists and implementation science researchers to address a critical gap in research and quality of healthcare for children and families who have experienced trauma-related behavioral health problems. The team examined barriers and facilitators to screening for trauma exposure and referring for specialized trauma-focused interventions in pediatric primary care; development of an intervention and implementation support package to increase appropriate screening, referral and follow-up activities; and a pilot study with 50 pediatric primary care providers to examine feasibility of the study design. Study findings indicate the importance of the ID/IP team collaboration to promote increased trauma screening and referral for trauma-exposed youth. The team was successful in obtaining extramural grant funds as a result, and plans to develop an evidence based training and implementation curriculum. This team was funded by the South Carolina Clinical and Translational Research Institute.

ID	Hub	Headline	Paragraph
142	UNIVERSITY OF ILLINOIS AT CHICAGO	Team Science: A University-Community Collaboration Promoting Positive Parenting in High Poverty Urban Communities	We had a problem. We were working with social service agencies to support children's mental health in impoverished Chicago elementary schools. Community providers were running school-based parenting groups that were poorly attended. We asked: could we modify the program to respond to parent needs and maximize community provider skills and talents? Over six months, agency supervisors and university researchers co-developed a service model --via weekly meetings, regular on-site visits, and feedback from providers and parents-- that integrated positive parenting principles into everyday contacts between community providers and parents. Throughout this, services remained ongoing in 16 schools to over 600 families. The new program was delivered to families across a school year and parenting skills were discussed in 69% of contacts. Additionally, the project resulted in one dissertation, one masters thesis, 7 publications, and an expanded understanding of who can deliver services and how they are delivered in underserved communities.
143	MEDICAL UNIVERSITY OF SOUTH CAROLINA	Team Work to Develop Novel Titanium Clip Metal Detector to Aid Breast Localization Surgery	Most breast biopsies today are of nonpalpable lesions identified by mammography. These lesions must be localized before excision by inserting a wire into the abnormal tissue under mammographic guidance. Wire localization requires a second invasive procedure and can cause logistical difficulties in the OR. A breast cancer surgeon at the Medical University of South Carolina (MUSC) wondered whether a metal detector could be used to detect a titanium chip directly, avoiding the need for localization surgery. To see if this was feasible, the surgeon contacted a bioelectrical engineer at Clemson University that she had met at a MUSC CTSA-sponsored retreat. The bioelectrical engineer challenged her design class to draw up plans for the detector, which they did, and, within a matter of months, the breast surgeon had a prototype she could test in specimens of breast tissue. The next step is to produce a prototype suitable for clinical trial.
144	UNIVERSITY OF TEXAS MED BR GALVESTON	Team-Centered Informatics: Unleashing the Power of Big Multiomics Data	Biomedical researchers are struggling to integrate many different types of human data such as genes, proteins, social interactions, and our environment to treat complex diseases like Alzheimer's and asthma. However, most informatics tools are designed to analyze just a few of these types of data in isolation by individual researchers. We therefore developed a visual representation (http://www.skbhavnani.com/DIVA/team-centered-informatics.html) that integrated many different types of human data in a single model, and observed how a multidisciplinary team used it to analyze a complex multiomics dataset of severe asthma patients. The visual representation acted as a "boundary object" which was meaningful to individual experts such as the biologist analyzing human proteins, but also provided an integrated understanding of all data types leading the team to precision medicine and translational insights for treating severe asthma. This research led to 6 multidisciplinary publications, 2 grants, and 2 research awards demonstrating the potential of team-centered informatics.

ID	Hub	Headline	Paragraph
145	MEDICAL UNIVERSITY OF SOUTH CAROLINA	Teamwork Evaluation Training and Intervention Improves Student Education, Patient Safety, Outcomes and Quality	At MUSC, 137 students from six health profession colleges underwent TeamSTEPPS® training and completed a standardized program for demonstrating teamwork rating competency. Teams of students observed real-time team behaviors of a hospital unit staff before and after the unit staff attended a teamwork-training workshop. Student ratings of unit teamwork behaviors (778 unique observations) increased significantly pre- to post- the unit-level intervention across the domains of team structure (p<.001), communication (p<.001), leadership (p<.001), situation monitoring (p<.001) and mutual support (p<.001). Significant decreases were observed in mean length of stay (LOS; 5.95% decrease; p=.05), mean number of LOS outliers (20.76% decrease; p=.03), and the mortality index (15.25% decrease; p=.03) after the unit-level teamwork training. Findings from this study suggest that student teamwork-rating-teams can gather important information to help guide teamwork interventions, and that teamwork training can improve care quality and safety.
146	UNIVERSITY OF WASHINGTON	The “I” in Team Science: Appointment, Promotion & Tenure (APT) in Interdisciplinary Research	APT criteria and exemplars highlight the success of individuals not teams; however, the literature demonstrates that interdisciplinary research teams are more successful in creating innovative approaches to addressing unmet needs than single author studies. Faculty who participate in team science are being evaluated by committees that may value this type research in theory but may not have experience in reviewing portfolios of interdisciplinary researchers. ITHS Team Science identified significant barriers to team science within 6 health science schools and the College of Engineering. In Fall 2018, ITHS Team Science will do the following: assess the attitudes of faculty reviewing APT portfolios through an online survey developed by ITHS Team Science with input from UW APT leads and CTSA members; conduct interviews with administrators about infrastructure and policies to facilitate team science; share survey tools with other CTSA members; establish exemplars supporting team science; and publish the results.
147	UNIVERSITY OF CINCINNATI	The Academic Tower of Babel: Team Science Bridges Across Cultures	Our Center for Improvement Science (Home for Team Science) has taken on the audacious task of bridging the 14 university colleges, a major pediatric care and research center, an academic health system, and large VA center in order to promote cross-disciplinary collaboration. We held two CTSA-wide retreats that included over 50 key stakeholders. Using a Polarity Thinking model to support research transformation we were able to identify a critical list of values and concerns that exist in the different organizational cultures. We identified the need for a common vocabulary and cultural “guide” and we have used these tools to create and support collaborative teams across colleges and institutions. We are currently fostering active collaborations across 10 colleges, the University Hospital, the pediatric center, and community partners. Investigators attend team science workshops and seminars to help support their new collaborations. Many teams have multiple external grants and publications.

ID	Hub	Headline	Paragraph
148	UNIVERSITY OF WISCONSIN-MADISON	The Evidence-to-Implementation Award: A model for accelerating outcomes of clinical and translational research	The Dissemination and Implementation (D & I) team at the University of Wisconsin's CTSA created the Evidence-to-Implementation (E2I) Award to encourage investigators to commercialize proven healthcare interventions developed at the university. To allocate funding towards the highest potential interventions, D&I team members from systems engineering, marketing, entrepreneurship, and medicine adapted a private-sector tool that accurately predicts the commercial viability of ventures based on evaluation of factors such as target customers, demand, and competition. Research teams worked collaboratively with D&I staff to develop business plans and marketing materials for their ventures. In March 2018, a trans-disciplinary panel composed of healthcare and business leaders evaluated seven proposals, assessing them against business concepts investigators used to develop their applications. Two funded awards (focused on surgical coaching and learning Tai Chi for falls prevention) are now using the E2I funding model to conduct market research, identify business strategies, and identify commercial partners for dissemination.
149	UNIVERSITY OF PITTSBURGH AT PITTSBURGH	The Pitt+Me® Research Recruitment Program is an Innovative Way to Engage Participants and Increase Enrollment	The Pitt+Me® Research Recruitment Program is a free service provided by the University of Pittsburgh CTSI that includes a participant registry, online advertising, social media, news stories, and videos. Since our Pitt+Me Registry launched in 2008, we have enrolled over 173,000 participants, made over 100,000 participant referrals, and helped more than 2,000 research projects meet their recruitment goals. For each study using Pitt+Me, our content team creates a custom online advertisement that explains the study in plain language, and social media posts for our Facebook and Twitter pages. Research results are disseminated through stories and videos on our Pitt+Me News site. To match Pitt+Me Registry participants with studies, our novel software incorporates participants' diagnosis codes, demographics, and heath preferences. Mailings alert participants when a study is available, and participants are prescreened online or through our call center. Potentially eligible participants are referred to study teams through an online portal.
150	UNIVERSITY OF WISCONSIN-MADISON	The Power of Teamwork: Helping Women Make Informed Decisions About Breast Cancer Screening	A multi-disciplinary team of researchers at UW-Madison recently spent 18 months working in close collaboration with the Wisconsin Network for Research Support (WINRS) and a Patient Advisory Committee (PAC). The project goals were to 1) generate patient feedback on a shared decision-making tool to improve breast cancer screening decisions and 2) use that feedback to strengthen the tool. The diverse team of clinician scientists brought knowledge about breast cancer screening options, information about available tools to support patient/clinician decision-making and nuanced understanding of patients' confusion with evolving mammography guidelines. WINRS contributed expertise on how to successfully recruit/orient patient panels and how to plan effective stakeholder meetings. The PAC provided critical patient-centered advice over the course of 5 highly interactive PAC meetings. Using a "Team Science" approach, this cohesive, multi-disciplinary group produced actionable outcomes, including patient-centered educational resources and improved decision aids to support clinician-patient communication and informed healthcare decisions.

ID	Hub	Headline	Paragraph
151	HARVARD MEDICAL SCHOOL	The Thai that binds: TL1-MD/PhD Grand Rounds at Harvard Medical School	For nearly a decade, graduate students in the Harvard Medical School TL1 program have worked in partnership with MD-PhD students at HMS to present clinical case studies that make connections to translational science. Our monthly TL1-MD/PhD Grand Rounds program engages leading clinicians and physician-scientist trainees at Brigham and Women's Hospital. The trainees work together to develop a clinical case that is discussed over dinner by Dr. Marshall Wolf, the legendary internist and former BWH residency program director. Following the clinical discussion, a selected student presents a synopsis of their PhD dissertation research and discusses its relevance to the clinical case just presented. All this transpires over a delicious Thai dinner. Student surveys have established that this interactive series involving graduate students (supported by the TL1), MD-PhD students (supported by the MSTP T32), clinicians, and physician-scientists improves communication and facilitates team-building between these key constituencies in the translational medicine workforce.
152	COLUMBIA UNIVERSITY HEALTH SCIENCES	TRaining And Nurturing Scientists FOr Research that is Multidisciplinary (TRANSFORM)	Team science is one of the main tenets of many programs within TRANSFORM, the education and career development resource at the Irving Institute for Clinical and Translational Research, home to Columbia University's CTSA Program hub. Over the past two grant cycles, we have further developed our multidisciplinary components within our KL2, TL1, and Masters of Science in Patient Oriented Research (MS-POR) programs. Beyond requiring all KL2 scholars and TL1 trainees to have interdisciplinary mentor teams, we meet with these mentor teams at least once a year to help develop Individual Development Plans (IDPs). Additionally, our KL2 scholars and MS-POR students are expected to take a one-semester course entitled, Building Interdisciplinary Research Models, in which attendees not only learn about developing meaningful interdisciplinary collaborations, but also apply concepts within their own research teams.
153	DUKE UNIVERSITY	Training program propels students ahead of their peers – real-world collaborative experience provides an obvious advantage.	The goal of the Biostatistics Core Training and Internship Program (BCTIP) is to prepare master's students to excel beyond their coursework through hands-on team-science exposure, and to be well-qualified biostatisticians immediately after graduation. Students are recruited twice per year through a competitive application and interview process. Interns undergo 6 weeks of training and 12-18 months of real collaborative experience that includes working directly with staff/faulty biostatisticians and clinicians in designing and analyzing research studies. BCTIP coordinators provide ongoing oversight and mentorship by tracking intern progress, collecting feedback from supervisors, and assessing advancement through progress reports and presentations. BCTIP has successfully trained >60 students with 100% job placement or PhD program enrollment. Additionally, BCTIP is working to create a larger-scale training program spanning many quantitative areas. For example, the Health Data Science Internship program has adopted the BCTIP model and now engages with BCTIP to share resources and enhance both programs.

ID	Hub	Headline	Paragraph
154	UNIVERSITY OF FLORIDA	Training the Translational Researchers of Tomorrow in Teams Today	The “Science of Team Science” tells us a lot about how established research teams work together, but how can we prepare trainees for interdisciplinary team science? To complement NIH training grants designed for individual trainees, our TL1 program is pioneering an innovative model for team-based training: PhD and combined-degree students apply for and receive support as TL1 Teams. Predoctoral trainees from different colleges develop team-specific aims to expand their PhD dissertation research, and undertake collaborative research to accomplish those aims. Through didactic training and coaching, a new Team Science course introduces students to evidence-based best practices for collaboration, which the students immediately apply to their projects. A second new course allows students to explore an area of common interest across all phases of translational research. Early assessment results reveal that trainees recognize advantages of engaging in interdisciplinary interactions, and overall level of confidence in conducting clinical research improves significantly.
155	WAKE FOREST UNIVERSITY HEALTH SCIENCES	Translational Research Scholars Learn to Form, Storm, Norm and Perform!	The Wake Forest CTSI’s Team Science Program collaborates with the KL2 Career Development Program by leading three 75-minute seminars as part of the Translational Scholars Academy curriculum where scholars learn team development norms and best practices and develop skills to lead teams more effectively. Seminars are delivered by team science faculty experts from our associated business school. Seminar topics were created in response to results from a needs survey from 42 research scholars accompanied by eight individual interviews. In response to felt needs, training revolves around a popular classical model of team development (Tuckman, 1965) involving stages of forming, storming, norming and performing. Participants engage in case analyses, problem solving, lectures and class discussions about the value and process of developing team charters, engaging all members in voice on the team, resolving conflict, planning and managing effective meetings, and follow-through. Seminar participants reported 93% satisfaction and a desire for more!
156	TUFTS UNIVERSITY BOSTON	Tufts CTSI Clinical Research Network Expands Trial Access for Network Partner Sites and Patients	Tufts Clinical and Translational Science Institute (CTSI) established a Clinical Research Network (CRN) comprised of an urban academic health center, regional community-based hospitals in urban, suburban, and rural areas, and other community hospitals and practices that reflect the diversity of hospitals across the US. The CRN is organized into cross-institutional infrastructure workgroups to develop an interactive network of functional teams working to reduce the time from receipt of a regulatory document set to site initiation. Thematic Expert Panels of experienced investigators rapidly review and distribute multi-site trial opportunities that originate from the Trial Innovation Network (TIN), individual investigators, or industry sponsors. Recently, two CRN partners were chosen to undergo formal site selection for a study offered via the TIN. Without the CRN, these sites would not have had the opportunity to participate in the trial, and their patients would not have the benefit of local access to clinical research.

ID	Hub	Headline	Paragraph
157	UNIVERSITY OF CALIFORNIA LOS ANGELES	UCLA CTSI Community Partnership Supports Expanded Eye Screenings for Underserved Patients with Diabetes.	Through a community partnership between the the UCLA CTSI and the Los Angeles County Department of Health Services, researchers discovered that offering diabetic retinopathy screening in primary care settings reduced examination waiting periods and increased the number of patients served. Implemented at 15 local primary care centers serving primarily low-income Latino communities, the screenings are maximizing access for this underserved population. The study, published in JAMA Internal Medicine, found that by conducting screenings in primary care, and transmitting retinal images to optometrists, the Los Angeles Department of Health Services eliminated the need for more than 14,000 visits to specialty care professionals, increased annual rates of screening for diabetic retinopathy by 16%, and reduced wait times for screenings by 89%. The study was the first to evaluate the effect of a system-level intervention on improving access to eye care and definitive treatment for diabetic retinopathy in an urban safety net population.
158	UNIVERSITY OF CALIFORNIA LOS ANGELES	UCLA CTSI-Supported Researchers Partner with Barbershops to Lower Blood Pressure	Black men are more likely to die from complications of high blood pressure than any other group in the U.S. To tackle this disparity, CTSI researchers from Cedars-Sinai and UCLA paired pharmacists with barbershops to offer high blood pressure care for customers. The results, published in the New England Journal of Medicine, showed the approach can dramatically lower blood pressure. The research team tracked blood pressure in the control and test groups at 52 barbershops for six months. Men in the control group, who were encouraged to see their doctor, continued to have high blood pressure after six months. But men in the test group, who met with a pharmacist at a barbershop, had large drops in their systolic blood pressure, from an average of 153 mmHg at the beginning of the study to 126 mmHg after six months. Normal systolic blood pressure is less than 120 mmHg.
159	UNIVERSITY OF CALIFORNIA, SAN FRANCISCO	UCSF CTSI K Scholar Team Science Awards - Promoting Innovative Multidisciplinary Research Collaborations	The UCSF CTSI K Scholars Program introduced a new Team Science competition in 2017. The goal is to stimulate research innovation and creativity by connecting K scholars with diverse training backgrounds and expertise to design and implement a scholarly project. Teams may propose pilot studies, clinical and/or translational methodologic innovations, or other research projects that demonstrate the strength of multidisciplinary collaborations. Team members are two or more K scholars that must have training, expertise, and a research portfolio in different disciplines. Proposals must demonstrate how linking two or more disciplines or content areas provides new insights and discoveries that advance clinical and translational science. Scholars submit formal applications that are reviewed by K Scholar Program faculty. Semi-finalists then present their projects to K Scholar colleagues who vote to select the final awardees. Teams receive up to $10,000 in funding and are required to provide periodic progress reports. The 2017 Awardees were “Improving Family Planning Care for Liver Transplant Recipients” and “Assessing the Validity of a Culturally Tailored Beverage Recall for Latino Children.” 2018 Awardees will be selected in late October.

ID	Hub	Headline	Paragraph
160	UNIVERSITY OF CALIFORNIA, SAN FRANCISCO	UCSF's Team Science Program: Bringing Diverse Researchers Together to Tackle Complex Problems	The Team Science Program (TSP) was launched as a new program within the UCSF Research Development Office in 2013. The goal is to foster collaborative research and support research teams, both intra- and inter-institutional, toward increasing institutional funding competitiveness and research innovation, all in support of the UCSF mission of advancing health worldwide. The Program offers a menu of services aimed at stimulating multi-investigator, multi-disciplinary efforts, from producing team-building events to supporting administrative needs of new teams. To date, the TSP has hosted 26 events, facilitated over 750 new introductions between faculty members, and sparked 300-plus ideas for collaboration. 83% of respondents to after-event surveys report continuing conversation with people they met at an event. Within one year of TSP events, 31% of respondents report initiating collaboration, 33% report developing a collaborative proposal, and 24% report receiving follow-on funding as a result of conversations initiated at a TSP event.
161	UNIVERSITY OF NEW MEXICO HEALTH SCIS CTR	University of New Mexico CTSC Hosts Hackathon – Innovation Teams "Hack" Solutions to Improve Health	UNM CTSC hosted the inaugural UNM Health Hackathon, May 18-20, 2018. This 3-day event employed a team science approach to stimulate innovation in health care, bringing together over 75 clinicians, engineers, entrepreneurs, programmers, scientists, and students to form 11 innovation teams. Many novel solutions or "hacks" to overcome health care challenges were created, along with a business plan to bring their "hack" to market. Mentors were drawn from the UNM faculty and community to assist teams throughout the event. 3D printers and engineering faculty consultation facilitated rapid prototyping. Teams and their "health hacks" were judged by local entrepreneurs. HSC Faculty on the two winning teams received UNM CTSC pilot awards up to $10,000 to prototype their products: VisiStik improved cane for the visually impaired; and FlexTrāch steerable endotracheal tube to improve intubations. Visual impairment affects 285 million people worldwide, and the improved endotracheal tube will decrease complications on surgical patients.
162	UNIVERSITY OF NEW MEXICO HEALTH SCIS CTR	UNM CTSC BioVenture Partnership Event	Over 125 people from the New Mexico academic and business community gathered for the UNM CTSC BioVenture Partnership to advance bioscience economic development. This networking event brought together teams of UNM faculty and local entrepreneurs to share stories of research that successfully translated laboratory technologies to the marketplace. Their stories stemmed from activities supported by the CTSC, which ranged from technologies developed from targeted RFAs - Intellicyt, direct CTSC PCIR support - VisionQuest Biomedical LLC, and even pilot funding - Agilvax. The event was part social gathering, an effort to encourage community engagement, and part competition. Following presentations by HSC faculty who have successfully led lab to market technology transfers, the event transitioned to a "shark tank" style pitch contest. Local entrepreneurs judged the pitches, and the CTSC was proud to see a KL2 scholar win. As a result, the event's coordinators hope for future commercialization in NM Bioventure industry.

ID	Hub	Headline	Paragraph
163	UNIVERSITY OF WISCONSIN-MADISON	UW as catalyst for qualitative team science -- a nationwide Health Experiences Research Network	The national Health Experiences Research Network (HERN) node at UW advances research on patients' experiences with health and health care using team science. Collaborative approaches are essential for knowledge generation, dissemination, and successful growth. For each qualitative study, multi-disciplinary teams of social scientists, clinicians, patient partners and staff from around the country collaborate on design; implementation; data analysis; interpretation of findings. Teams meet weekly throughout each project, providing the diverse perspectives essential for high-quality research products. Dissemination of HERN's web-based modules is also a team effort: communication specialists, media specialists, researchers, and patient experience ambassadors develop innovative strategies. HERN's national steering committee engages representatives of consumer groups, academics from multiple disciplines, policy experts, and evidence-based medicine leaders in order to nurture new multidisciplinary teams; assure on-going quality; and adapt international team-based approaches for the U.S. context. Successes: nine studies and three dissemination projects funded; learning network; 4,600 visits/month to website.
164	VCU	VCU Researchers and CrossOver Healthcare Ministry Collaborate to Improve Oral Health in Local Immigrant Population	The Wright Center for Clinical and Translational Research is pleased to announce funding for a pilot project from two faculty researchers who are part of Virginia Commonwealth University's iCubed Oral Health Initiative. Dr. Sarah Raskin, Assistant Professor, Wilder School, and Dr. Aderonke Akinkugbe, School of Dentistry, were awarded $130,000 for their interdisciplinary, research collaboration with CrossOver HealthCare Ministry, a local free clinic serving a largely low-income, immigrant population. In this project, Drs. Raskin and Akinkugbe will work with CrossOver clinicians to identify modifiable individual, clinical, cultural, and health services delivery-related factors predictive of dental care and evaluate implications of dental service underutilization in this population. Quantitative and qualitative data will be collected, analyzed, and disseminated in order to improve dental utilization in the short-term, and, in the long-term, benefit underserved patients' oral health and reduce dental disparities.
165	VCU	Vitamin C Is Curing Sepsis: Launching a Multi-Center Team to Treat Sepsis with IV Vitamin C	Led by Dr. Alpha Fowler, with resources from VCU's Wright Center, a multi-center research team was developed from 7 academic medical centers to study patients with severe sepsis and acute respiratory distress syndrome, a lethal combination that kills 45 – 50 % of patients. This team science example was funded through a NHLBI multi-million dollar grant. The team goal was to determine if an around the clock, 4-day intravenous infusion of vitamin C would save lives from a disease that has no direct therapy and kills a patient in the U.S. every two minutes. The teams enrolled 167 patients: 83 patients received sugar water and 84 patients received IV vitamin C. All patients received ICU standard of care. Vitamin C significantly improved survival in vitamin C infused patients. This example of team science has been used as an illustration and case study of how team science can improve human health.

ID	Hub	Headline	Paragraph
166	OREGON HEALTH & SCIENCE UNIVERSITY	We want to make Oregon healthy – Will you join us?	The Healthy Oregon Project (HOP) aims to establish a cohort of at least 100,000 Oregonians to study the influence of genetics, environment, and behavior on the risk for chronic diseases, including cancer. The first HOP project is funded by the Knight Cancer Institute's Cancer Early Detection Advanced Research Center and will provide population-wide screening for clinically actionable, heritable cancers. For Phase 1 in fall 2017, project leaders leveraged the OHSU Community Research Hub's extensive network to engage 200+ community members in 35 focus groups in 24 counties within a 6 week period to learn about local attitudes toward genetic screening and willingness to participate in research. Results were used to secure funding for Phase 2, which includes scaling up project structure and recruiting additional community partners and scientific experts to give input on study design, sample collection, and human subjects' protection. This collaborative project highlights the importance of integrating community members on our research teams to create mutually beneficial outcomes.
167	INDIANA UNIV-PURDUE UNIV AT INDIANAPOLIS	WeCare INDIANA: An Academic-Community-Government Partnership Works Together to Reduce Risk Factors contributing to Infant Mortality	Beginning in 2016, evidence-based practices to reduce risk factors for infant mortality are being implemented in several high-risk zip code regions. Community health workers (CHWs) armed with clinical decision support and mHealth technology tools are actively coaching over 1500 pregnant or post-partum women and connecting them with community resources. Weekly guided practice sessions are held with the CHWs, employing the University of New Mexico's ECHO videoconferencing methods, allowing CHWs working in their respective communities to participate using the zoom platform. Guest participants including community organization leaders, community members, medical personnel, first responders, and policy makers join the interdisciplinary WeCare panel to provide on-going education, guidance and support for CHW who provide front line coaching to enrolled women. To date, improvement in all key risk factors (smoking, mental health, food insecurity, substance use, safe sleep practices, and breastfeeding) has been documented. Over 97% of births have had normal birth weights.
168	OREGON HEALTH & SCIENCE UNIVERSITY	Welch-Allyn Engineering Rounds at Oregon Health & Science University Sparks Medical Innovation	A strong partnership between OCTRI, OHSU Technology Transfer & Business Development, and Welch-Allyn helped establish regular engineering rounds, allowing medical equipment design engineers the opportunity to engage doctors and patients. The long-term goal of this arrangement is to translate unmet medical needs into innovations and products that improve patient outcomes. The chance to observe, ask questions, and receive feedback is essential to engineers in any industry, but is typically difficult in the medical industry. By allowing engineers to ask questions and connecting them with physicians who are willing to immerse them in a patient care environment, it opens the door to a wide variety of opportunities to improve medical care. One project, a collaboration between Welch-Allyn engineers and two ED doctors aims to reduce suicide attempts in young adults by creating a wearable device that monitors certain physiological parameters. The prototype is currently being used in an ongoing clinical trial.

ID	Hub	Headline	Paragraph
169	UNIVERSITY OF KENTUCKY	Western States Consortium study on OUD in pregnancy.	In Oct 2017, members of the Western States Consortium (Arkansas, Kansas, Kentucky, New Mexico, Utah) gathered for its annual summit, which included discussions of mutual interest including the opioid epidemic, which is a major health problem for each institution. From this summit came a commitment for joint pilot studies which resulted in an RFA and the submission of 18 pilot grants involving 2 or more of the partner institutions. After peer review, a study was jointly funded by Arkansas, Kentucky, New Mexico and Utah entitled “Models of care for pregnant women with opioid use disorder, and infants with opioid exposure”. This best practices study involves PIs at each institution, all with expertise in OUD in the perinatal period, from different disciplines (Primary Care, Nursing, Ob/Gyn and Psychiatry). By developing a best practice of care in 4 institutions, it is hoped that this can be developed into a clinical trial that will impact care broadly in the US.
170	MEDICAL UNIVERSITY OF SOUTH CAROLINA	Wide Spectrum Investigation of Stroke Outcome Disparities on Multiple Levels (WISSDOM)	Wide Spectrum Investigation of Stroke Outcome Disparities on Multiple Levels (WISSDOM) A team of cross-disciplinary scientists (basic scientists, neurologists, nurse scientists and population scientists) at the Medical University of South Carolina was formed to address disparities in patient outcomes following stroke. The WISSDOM program spans the translational research spectrum. With no animal model for stroke, the team took up the challenge to develop a novel rodent model with multiple pre-existing stroke risk factors similar to population characteristics of African American (AA) stroke patients (Basic research); evaluate brain structural features in patients with Stroke (Clinical Project); and designed a population project to improve stroke outcomes with a Community Intervention Nurse Guided approach (Implementation). The team hosted a “Disparities Summit” to disseminate the outcomes and look for opportunities to train the Fellows. The team obtained two cooperative projects to further basic and clinical aspects of the program. This is a case example of a real ‘Team’ approach that can be shared to implement with other teams.
