# Supplementary material for: What does team science look like across the CTSA consortium? A qualitative analysis of the Great CTSA Team Science Contest submissions
Source: J Clin Transl Sci. 2021 Jul 12;5(1):e154. doi: 10.1017/cts.2021.812 (PMC8411266; doi:10.1017/cts.2021.812)
Supplement: Supplementary file 1 [file S2059866121008128sup.zip › S2059866121008128sup002.pdf]

Close Preview | Restart Survey | Draft | Place Bookmark

THE GREAT CTSA TEAM SCIENCE CONTEST

Welcome to The Great CTSA Team Science Contest!

The purpose of this contest is to find the best ideas in all of CTSA-land for encouraging better team science. Any person associated with a CTSA hub is eligible. It can be the hub PI, program director, staff member, trainee, student, a participating researcher, a community member et al.

INSTRUCTIONS
Think of something that you have seen or done at your hub's institution(s) that you think has helped to advance team science. It could be a program, a support service, an event, or anything else. Here are some examples: approaches to facilitate research collaborations; academic-community team partnerships; teaching and training in team science; incentives for team work; evaluation measures of team science; lessons learnt. Then, provide the information requested below. You can submit more than one submission per hub.

If you need more information or have any general questions about the contest, please contact either Deborah DiazGranados, Ph.D. of Virginia Commonwealth University (deborah.diazgranados@vcuhealth.org) or Rebecca Moen, MBA of Duke University (rebbecca.moen@duke.edu). For technical issues related to this website, contact William Trochim, Ph.D. (wmt1@cornell.edu).

The Judging. We have assembled a crack team of the best team science researchers and managers in all of CTSA-land. Multiple judges will be asked to read each contest submission (without seeing what person or hub it's associated with) and rate it for: Importance, Innovation and Impact. The highest-rated submissions will be declared the gold, silver and bronze award winners.

The Prize. The winners will receive absolutely no prize money, no additional grant funds and, in fact, nothing of any monetary value whatsoever! They won't get a medal or a trophy. Instead, they'll get something every CTSA desires and finds so hard to achieve – ***bragging rights***! The winning hubs will be announced at a major CTSA event and the results forever recorded on the world-wide internet somewhere.

We think this approach can give us a good picture of the kinds of innovations in team science that the CTSAs are involved with. Just the summaries of all the stories submitted and their classification into types of innovations will be useful evaluation information. The results can point us in the direction of examples that we could follow -up in greater detail. As a result, longer stories about the winning projects will be written up in the CTSA newsletter.

The Headline
In 15 words or less, concoct a short headline for an imaginary newspaper article about the team science innovation at your hub (for example, a headline might be something like "Come Together: A Program for Launching Biomedical Research Teams").

The Paragraph
(150 words max!). Provide the bare essentials that summarize the "story" of your team science innovation. This should include the basic "five-W's": Who, What, Where, When, and Why, and as much of the 'How' as you can fit. Also, tell us briefly how you know it worked or was successful. (Please Note: You can resize the text box to make it larger if you wish).

Close Preview Restart Survey Draft Place Bookmark

hub it's associated with) and rate it for: Importance, Innovation and Impact. The highest-rated submissions will be declared the gold, silver and bronze award winners.

The Prize. The winners will receive absolutely no prize money, no additional grant funds and, in fact, nothing of any monetary value whatsoever! They won't get a medal or a trophy. Instead, they'll get something every CTSA desires and finds so hard to achieve – ***bragging rights***! The winning hubs will be announced at a major CTSA event and the results forever recorded on the world-wide internet somewhere.

We think this approach can give us a good picture of the kinds of innovations in team science that the CTSAs are involved with. Just the summaries of all the stories submitted and their classification into types of innovations will be useful evaluation information. The results can point us in the direction of examples that we could follow -up in greater detail. As a result, longer stories about the winning projects will be written up in the CTSA newsletter.

The Headline

In 15 words or less, concoct a short headline for an imaginary newspaper article about the team science innovation at your hub (for example, a headline might be something like "Come Together: A Program for Launching Biomedical Research Teams").

The Paragraph

(150 words max!). Provide the bare essentials that summarize the "story" of your team science innovation. This should include the basic "five-W's": Who, What, Where, When, and Why, and as much of the 'How' as you can fit. Also, tell us briefly how you know it worked or was successful. (Please Note: You can resize the text box to make it larger if you wish).

The Hub

Select the hub you are associated with:

→

Close Preview | Restart Survey | Draft | Place Bookmark

THE GREAT CTSA TEAM SCIENCE CONTEST

Your Name:

Your contact e-mail address (just email address - no names or spaces):

→

Powered by Qualtrics

Close Preview | Restart Survey | Draft | Place Bookmark

THE GREAT CTSA TEAM SCIENCE CONTEST

Please review the following story:

Submission Date:

Headline:

Paragraph:

Please rate this story for its **Importance, Innovation and Impact as listed below.** Please use a scoring scale of 1-10 when you rate, where a score of 1 as the 'Relatively Unimportant' and a score of 10 as the 'Extremely Important'.)

Importance (i.e. Does the story address an important area/problem or a critical barrier to progress in the field of team science?)

Relatively Unimportant				Moderately Important				ExtremelyImportant
1	2	3	4	5	6	7	8	9

Importance

Innovation: (Does the submission challenge and seek to shift current practice by utilizing a novel approach/concept/intervention?)

Relatively Uninnovative				Moderately Innovative				Extremely Innovative
1	2	3	4	5	6	7	8	9

Innovation

Impact (Likelihood of having a sustainable approach, potential for developing best practices and implementation)

No Impact				Moderate Impact				Exemplary Impact
1	2	3	4	5	6	7	8	9

Impact

Close Preview Restart Survey Draft Place Bookmark

of team science?)

Relatively Unimportant				Moderately Important			ExtremelyImportant	
1	2	3	4	5	6	7	8	9

Importance

Innovation: (Does the submission challenge and seek to shift current practice by utilizing a novel approach/concept/intervention?)

Relatively Uninnovative				Moderately Innovative			Extremely Innovative	
1	2	3	4	5	6	7	8	9

Innovation

Impact (Likelihood of having a sustainable approach, potential for developing best practices and implementation)

No Impact				Moderate Impact			Exemplary Impact	
1	2	3	4	5	6	7	8	9

Impact

Please select one or more categories into which you would classify this story:

- ☐ Workforce Development. The translational science workforce has the skills and knowledge necessary to advance translation of discoveries.
- ☐ Collaboration/Engagement. Stakeholders are engaged in collaborations to advance translation.
- ☐ Integration. Translational science is integrated across its multiple phases and disciplines within complex populations and across the individual lifespan.
- ☐ Methods/Processes. The scientific study of the process of conducting translational science itself.
- ☐ Informatics. Innovative informatics solutions are used to advance translational research, train the CTSA workforce, disseminate best practices, engage communities of the stakeholders, and integrate clinical and basic research data.
- ☐ Other. Please specify in a few words:

→

Powered by Qualtrics

Close Preview | Restart Survey | Draft | Place Bookmark

THE GREAT CTSA TEAM SCIENCE CONTEST

Do you have any conflicts of interest (COI) that would make it difficult for you to review this story without bias (For purposes of this review, a COI can be described as a reviewer who is directly collaborating with any member of the story team or is at the same hub from which the story originated)?

- Yes, I have a Conflict of Interest and cannot review this story
- No, I don't have a Conflict of Interest and I can review this story

→

Powered by Qualtrics
