# Supplementary material for: What does team science look like across the CTSA consortium? A qualitative analysis of the Great CTSA Team Science Contest submissions
Source: J Clin Transl Sci. 2021 Jul 12;5(1):e154. doi: 10.1017/cts.2021.812 (PMC8411266; doi:10.1017/cts.2021.812)
Supplement: Supplementary file 1 [file S2059866121008128sup.zip › S2059866121008128sup003.pdf]

Table A2. Codebook for Qualitative Data Analysis. Qualitative data analysis coding structure of the GTSC. These are the category and sub-category names and definitions that we explored in the GTSC submissions.

Name	Description
1. Category -Type	Submission falls into a category for Workforce Development, Methods & Process, Collaboration/Engagement, Informatics, Integration, Other
Community & Collaboration	Community Engagement is to engage communities in a meaningful way around specific projects and to advance the science of community engaged research. Collaboration and Multi-Disciplinary Team Science is to facilitate approaches to solving problems that cut across disciplinary boundaries, with the goal of advancing clinical research and translation. Faculty and KL2 training are included, but does not include Pilot awards, which go in Translational Endeavors.
Hub Research Capacity	Hub Research Capacity (HRC) includes Integrating Special Populations (ISP), to ensure that breakthroughs are quickly translated to the benefit of society and all subpopulations within. HRC also includes Participant and Clinical Interactions (PCI) to ensure that high quality human subjects research is conducted at their institutions and that scientifically or ethically flawed studies are identified, disapproved, and used as an educational example at an individual and institutional level.
Informatics	Informatics: development, demonstration, and dissemination of informatics and IT innovations that accelerate both the science and operations of clinical translation.
Network Capacity	Network Capacity includes the Hub Liaison Team (HLT) and the Trial Innovation Network (TIN). Network Capacity will function as an interface between the CTSA hubs and the national collaborative activities of the CTSA Program, including TIN, RIC, TIC, CTSA CLIC and CD2H.
Research Methods	Research Methods includes the components of Biostatistics, Epidemiology and Research Design and Regulatory Knowledge Services.

Name	Description
Translational Endeavors	Includes Translational Workforce Development (TWD)-development of a multidisciplinary translational workforce at the CTSA Program hub. This needs to be well integrated with the Career Development and Training components. Pilot Translational and Clinical Studies Program (PTC)-provide funding to support translational research projects of limited scope, duration, and budget. Includes other research staff like clinical coordinators, program managers, students training how to be researchers and TS.
CTSA component was not explicitly specified within the submission	There is no explicit evidence that indicates which core it belongs to.
2. Who - Team	Who is the team working on this project? Researchers, Multi-disciplinary teams, Multi-CTSA Hubs, CTSA core, KL2, Unknown
Community	Community members or community organizations are part of the team sponsoring this team science activity.
CTSA Core	One or more of the of the CTSA cores are part of the team sponsoring this team science activity.
KL2	KL2 Scholar(s) is/are part of the team sponsoring this team science activity.
Multi-CTSA hubs	More than one CTSA hub are part of the team sponsoring this team science activity.
Multi-disciplinary teams	Multi-disciplinary teams that do not fall in the other categories are part of the team sponsoring this team science activity.
Team was not explicitly specified within the submission	Who is sponsoring this team science activity is not explicitly specified within the submission.

Name	Description
3. Who - Target	WHO – is the Target in terms of impact? What group(s) are the intended beneficiaries of this team science activity?
Team scientists	The study is intended to impact team science or members of multidisciplinary teams and can include training at any level.
Community - Public Health	The community will be impacted through community interventions or through improvements in public health.
Patients included in study design	Study will impact patients in a trial or in a hospital or health care setting
Students	Can be pre-doctoral or doctoral or post-doctoral students
Undergraduate and graduate students	Can be pre-doctoral or doctoral
K Scholars	K Scholars are intended to benefit.
4. Intended Outcome	WHY – What was the intended outcome of the team science submission?
Team science skills and process	
Training in team science competencies	Training in team science competencies covered 25 evidence-based competencies involving the seven dimensions of leadership, communication styles and adjusting for different styles, and workshops on leading multidisciplinary teams.
Training in communication skills	Training in communication was prominently represented and took many forms. Communication skills workshops were designed to promote smoother team functioning.

Name	Description
Providing pilot funding or specific awards to promote existing team science	Providing pilot funding and special awards for promising teams is a frequently used strategy to promote team science.
Training undergraduates, graduates and post-doctoral students in team science	Training undergraduate-, graduate- and post-doctoral-students improves the team science pipeline.
Developing a team charter or engaging in team building	Team building and developing team charters are important aspects of team science process which detail many aspects of team function including direction, roles and how team members should interact.
Leadership or mentorship training	Leadership or mentorship training was one component of multi-faceted programs that also addressed innovation, adaptation, project management and communication.
Training in technology ventures or entrepreneurship	Developing technology or entrepreneurial skills focused on developing technological solutions for specific unmet healthcare needs.
Community- Getting the community involved in translational research	Getting the community involved in translational science is a team science outcome that addresses community health needs.
Other team science activities	If the desired outcome does not fit in the other nodes.
Fostering new multidisciplinary collaboration (e.g. retreats, new pilot grants, hackathons)	Fostering new multidisciplinary collaborations included bringing together researchers from different disciplines to brainstorm solutions to a specific challenge.

Name	Description
Multidisciplinary development of new tools in Informatics (e.g. for collaborator discovery, funding discovery, team function)	These were often collaborations between bioinformatics researchers, computational biologists or biostatisticians who were helping researchers solve specific problems by developing devices or apps. The submissions fell into six categories: 1) Medical apps developed for portable devices to manage chronic conditions; 2) Informatics solutions for data discovery such as finding collaborators or biomarkers or developing algorithms (e.g. train AI devices to read radiological images); 3) Solutions to combine data of different types into repositories and link those data to patients' electronic health records; 4) Medical device development or improvement (e.g. a cane for the visually impaired or a device to make breast surgery less invasive or an endotracheal tube with significantly fewer complications); 5) Videogames used for skill training the next generation of healthcare workers; 6) Programs to train informatics students to work in multidisciplinary teams or to help teams of disparate health professionals with workflow in the emergency department.
Workforce Development	Workforce Development with research personnel included training health equity researchers, health system leaders and community health workers in programs that integrate transdisciplinary research, community activation, education, and policy translation.
Expanding translational science (TS) capacity	Expanding capacity to conduct translational science (TS) consisted of developing new systems or relationships to increase TS.
Advancing promotion & tenure (P&T) for team science	The P&T outcomes had to do with helping chairs understand what it takes for a researcher to successfully get P&T as a team scientist and doing extensive studies of the faculty, chairs, leadership and climate at an institution so as to understand the barriers that much be overcome.
Example of multidisciplinary collaborative teams doing disease-specific or wellness interventions	Specific successful examples of multidisciplinary collaborative teams doing disease-specific or wellness interventions. These covered areas of wellness/prevention, chronic disease, cardiovascular disease, mental/behavioral health, addiction, genetics, infectious disease, reducing health disparities, cancer and others. These generally did not detail strategies to promote team science. If an example described a team science intervention with an intended outcome, then it was moved to the relevant intended outcome category.

Name	Description
5. How - Method	HOW (Process/methods) – was the intervention brought about? What team science method or strategy did they use?
Team science research, intervention, resource	Study deals with studying the science of team science, is an intervention to improve team science or teams, or is a resource for team science.
Training program for team science	Team science training and research activities, including leadership research and training for K-scholars and trainees, symposia to discuss best practices and collaborative research
Developing NEW Teams (e.g. with pilot funding)	Team science interventions or resources to foster new teams. Submitters organized symposia and workshops around specific topics of interest to bring researchers together to develop ideas for new proposals. Funding opportunities developed for multidisciplinary teams took the form of awards honoring specific scientists and competitive award offerings.
Community collaboration with teams	Team science interventions that specifically focused on getting community members or community organizations involved in team science or training.
Other	A team science intervention or method that is NOT a community collaboration, NOT developing new teams with pilot funding and NOT an explicit training "program".
Example of team science	Unique example of a specific team science project.
6. TS Spectrum - Stage	Classify as Basic Research, Pre-clinical research, Clinical, Clinical Implementation, Public Health (related to translational spectrum)
Basic Research	Scientific exploration that can reveal fundamental mechanisms of biology, disease or behavior.

Name	Description
Clinical Implementation	The clinical implementation stage of translation involves the adoption of interventions that have been demonstrated to be useful in a research environment into routine clinical care for the general population. This stage also includes implementation research to evaluate the results of clinical trials and to identify new clinical questions and gaps in care.
Clinical Research (Trials)	Clinical research includes studies to better understand a disease in humans and relate this knowledge to findings in cell or animal models; testing and refinement of new technologies in people; testing of interventions for safety and effectiveness in those with or without disease; behavioral and observational studies; and outcomes and health services research. The goal of many clinical trials is to obtain data to support regulatory approval for an intervention.
Other	Does not fit into the other TS Spectrum stages
Pre-clinical research	Pre-clinical research connects the basic science of disease with human medicine. During this stage, scientists develop model interventions to further understand the basis of a disease or disorder and find ways to treat it. Testing is carried out using cell or animal models of disease; samples of human or animal tissues; or computer-assisted simulations of drug, device or diagnostic interactions within living systems.
Public health, Policy, Prevention	In this stage of translation, researchers study health outcomes at the population level to determine the effects of diseases and efforts to prevent, diagnose and treat them. Findings help guide scientists working to assess the effects of current interventions and to develop new ones.
SciTS = Science of Team Science	SciTS examines the processes by which large and small scientific teams, research centers, and institutes organize, communicate, and conduct research. It is concerned with understanding and managing circumstances that facilitate or hinder the effectiveness of collaborative research, including translational research. This includes understanding how teams connect and collaborate to achieve scientific breakthroughs that would not be attainable by either individual or simply additive efforts.

Name	Description
7. NCATS strategic objectives	How are hubs using team science to address the NCATS strategic objectives?
1) Engage patients, community members and nonprofit organizations in the translational process	Multidisciplinary team science initiatives that employed team science strategies directly involving patients included funding multidisciplinary research for developing translational diagnostic, treatment, and disease self-management approaches. Other initiatives linked primary care service providers and researchers for clinical trials and care. Team Science strategies involving communities included a variety of research and implementation projects: researchers and community members in joint initiatives to reduce health disparities, reduce high blood pressure, opioid abuse, improve oral health among migrants, combat cancer and rare genetic disorders. Submissions described partnerships that addressed healthy eating and obesity. In communities and state governments, academics and communities joined in programs to reduce risk factors of infant mortality, identify a rare genetic variant in newborns, promote positive parenting and screen and identify victims of child abuse. Other collaborative partnerships focused on health promotion and wellness programs for older adults, including those in low-income housing.
2) Share resources through collaborative research with other NIH Institutes and Centers	Sharing resources and expertise across the federal government through collaborative research with other NIH institutes and Centers.
3) Form innovative collaborations with multidisciplinary scientists at other institutions	Multidisciplinary submissions involved multi-state consortia as well as multi-CTSA hub consortia around pressing health problems.
4) Develop new collaborative structures with the private sector	Developing collaborative structures with the private sector, most of the submissions that involved the private sector concerned helping programs to scale inventions to help develop commercial health products or public health interventions.

Name	Description
8. Where - Geographic location-US Region	Which USA region was the CTSA hub located in? Northeast, Southeast, Midwest, West, Southwest. For multi-hub collaborations, count every hub.
Midwest	OH, IN, MI, IL, MO, IA, MN, WI, ND, SD, NE, KS
Multi-region Multi-CTSA	Involves a collaboration between 2 or more CTSA hubs
Northeast	ME, VT, MA, NY, PA, RI, CT, NJ, NH
Southeast	MD, DE, DC, WV, VA, KY, TN, NC, SC, GA, AL, MS, LA, AR, FL
Southwest	AZ, NM, TX, OK,
West	WA, OR, ID, MT, WY, CO, UT, NV, CA, AK, HI
